# MNT suppresses T cell apoptosis via BIM and is critical for T lymphomagenesis

**DOI:** 10.1038/s41418-023-01119-y

**Published:** 2023-02-08

**Authors:** Hai Vu Nguyen, Cassandra J. Vandenberg, Mikara R. Robati, Ashley P. Ng, Suzanne Cory

**Affiliations:** 1 The Walter and Eliza Hall Institute of Medical Research, Melbourne, VIC Australia; 2 Department of Medical Biology, University of Melbourne, Melbourne, VIC Australia

**Keywords:** Molecular biology, Experimental models of disease

## Abstract

The importance of c-MYC in regulating lymphopoiesis and promoting lymphomagenesis is well-established. Far less appreciated is the vital supporting role of MYC’s relative MNT. Using *Rag1Cr*e-mediated *Mnt* deletion in lymphoid progenitor cells, we show here that, during normal T cell development, MNT loss enhances apoptosis, at least in part by elevating expression of the pro-apoptotic BH3-only protein BIM. Moreover, using T lymphoma-prone VavP-*MYC* transgenic mice, we show that *Mnt* deletion reduces the pool of pre-malignant MYC-driven T lymphoid cells and abrogates thymic T lymphomagenesis. In addition, we establish that *Mnt* deletion prevents T lymphoma development in γ-irradiated mice, most likely by enhancing apoptosis of T lymphoid cells repopulating the depleted thymus. Taken together with our recent demonstration that MNT is vital for the survival of MYC-driven pre-malignant and malignant B lymphoid cells, these results suggest that MNT represents an important new drug target for both T and B lymphoid malignancies.

## Introduction

The transcription factor c-MYC (hereafter MYC) controls expression of numerous genes involved in the proliferation, growth, metabolism and DNA damage responses of normal cells in adult tissues [1, 2] and its deregulated over-expression is a major driver of human cancer [3, 4]. However, stressed cells expressing elevated MYC are prone to apoptosis [5–7], which serves as a critical restraint on neoplastic transformation. Hence, genetic defects that impede apoptosis boost MYC’s oncogenic potential, as first revealed by the seminal demonstration that anti-apoptotic BCL-2 synergises with MYC in lymphomagenesis [8, 9].

To activate transcription, MYC heterodimerises with MAX, another ubiquitously expressed basic Helix-Loop-Helix Leucine Zipper (bHLHLZ) protein, and together they bind E-box motifs (CACGTG) in target genes [1]. However, MAX also binds several MYC-related transcriptional repressors containing bHLHLZ domains [1, 10]. MNT, an important member of this MXD (MAX Dimerization) family of c-MYC antagonists [11–13] is essential for embryonic development [14] and widely expressed in mammalian tissues.

We recently established that MNT is critical for lymphomagenesis in *Eμ-Myc* transgenic mice [15], which model human Burkitt’s lymphoma [16], and showed that MNT aids MYC by suppressing apoptosis in both pre-malignant and fully malignant B lymphoid cells.

Many human T cell neoplasms are associated with poor prognosis and new therapeutic approaches are sorely needed [17, 18]. Here, building on earlier studies [19–21], we investigate how MNT loss, mediated by a *Rag1Cre* transgene in lymphoid progenitor cells [22], impacts normal T cell development and T lymphomagenesis. We confirm that MNT loss promotes T cell apoptosis and provide new genetic and biochemical evidence regarding the underlying mechanism. We establish, for the first time, that MNT loss reduces the competitive fitness of T (and B) lymphopoiesis. We show that MNT loss enhances apoptosis of developing T lymphoid cells and abrogates T lymphoma development in vavP-*MYC* transgenic mice and γ-irradiated C57BL/6 mice. Our studies encourage investigation of MNT as an important new therapeutic target.

## Materials and methods

### Mice

Mice used were *Mnt*^fl/fl^ [23], *Rag1Cre* [22], *MYC10*^*hom*^ [24] and *Bim*^−/−^del339 [25], all on a C57BL/6 background. Note that homozygous *Mnt* deletion, initially reported as perinatal lethal [14], is fatal at ~E10 in C57BL/6 mice bred in our facility [15]. Details of breeding, genotyping, immunophenotype analysis, OP9-DL1 co-culture, CFSE labelling and CRISPR/Cas9 genome editing are provided in Supplementary Materials and Methods and figure legends.

### Competitive bone marrow reconstitution assays

C57BL/6 mice (Ly5.1^+^) (3 per test) were lethally irradiated (2 × 5.5 Gy) and reconstituted with 10^6^ bone marrow cells from Ly5.1^+^ C57BL/6 mice (competitor cells) and 10^6^ Ly5.2^+^ bone marrow cells harvested from either *Mnt*^*+/+*^ *Rag1Cre* (3 F and 1 M) or *Mnt*^*f*l/fl^ *Rag1Cre* (2 F, 3 M) mice (test cells). Ly5.1^+^ competitor and Ly5.2^+^ test cells of the same sex were injected into Ly5.1^+^ recipient mice of the same sex. Twelve weeks after transplantation, the relative proportions of test cell-derived lymphoid and myeloid cells (Ly5.2^+^) and competitor cell derived lymphoid and myeloid cells (Ly5.1^+^) of the indicated cell subsets in the thymus, spleen and bone marrow were determined by flow cytometry, using Ly5.1 and Ly5.2 antibodies to distinguish the two types of competing cells (see Supplementary Fig. 1).

### Radiation induction of T lymphomas [26]

33 day-old C57BL/6 mice (WT, *Mnt*^*+/+*^ *Rag1Cre* and *Mnt*^*fl/fl*^ *Rag1Cre*) were exposed weekly, 4 times, to 1.5 Gy γ-irradiation from a 60^Co^ source (Theratron Phoenix, Theratronics) and monitored until 350 days old. Sick mice (evidenced by breathing difficulties, enlarged thymus and/or enlarged spleen) were euthanised, autopsied and tumours characterised by immunophenotyping and immunoblot expression analysis.

### Statistical analysis

Statistical comparisons were made using unpaired two-tailed Student’s *t-*test with Prism v8.0 software (GraphPad, San Diego, CA, USA). Data are shown as means ± SEM with *P* ≤ 0.05 considered statistically significant. Mouse survival analysis was carried out using GraphPad Prism (Version 8.0) and significance determined using log-rank (Mantel–Cox) test.

## Results

### MNT loss in lymphoid cells reduces competitive fitness

**Fig. 1:**
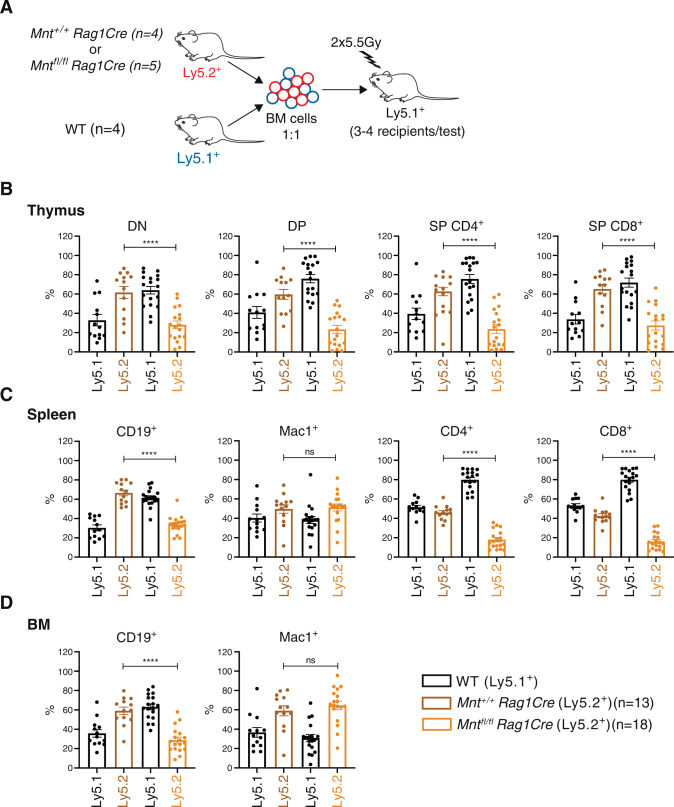
Bone marrow reconstitution experiments reveal competitive disadvantage of MNT-deficient lymphopoiesis. **A** Protocol for competitive reconstitution. C57BL/6 mice (Ly5.1^+^) (3–4 per test) were lethally irradiated (2 × 5.5 Gy) and injected with a 1:1 mixture of bone marrow cells (10^6^ cells per genotype) from Ly5.1^+^ C57BL/6 mice (competitor cells) and either *Mnt*^*+/+*^*Rag1Cre* or *Mnt*^*fl/fl*^*Rag1Cre* mice (Ly5.2^+^ test cells). **B**–**D** Relative proportions of reconstituted test cells (Ly5.2^+^) and competitor cells (Ly5.1^+^) of the indicated cell types in the thymus (**B**), spleen (**C**) and bone marrow (**D**), 12 weeks post-transplantation. Analysis was performed by flow cytometry (see Supplementary Fig. S1). Data shown are from four independent reconstitution experiments; genotypes are WT (Ly5.1^+^, black), *Mnt*^*+/+*^*Rag1Cre* (Ly5.2^+^, brown) and *Mnt*^*fl/fl*^*Rag1Cre* (Ly5.2^+^, gold). Dots indicate individual reconstituted mice; bars show mean ± SEM; statistical significance is shown only for *Mnt*^*+/+*^*Rag1Cre* versus *Mnt*^*fl/fl*^*Rag1Cre* cells; ****P ≤ 0.0001, ns not significant.

To avoid embryonic lethality conferred by MNT loss [15], we deployed the *Rag1Cre* transgene, which is expressed only in early lymphoid progenitors [22]. This strategy enabled us to compare MNT-deficient *vs* normal lymphopoiesis in adult mice, using competitive bone marrow reconstitution. Lethally irradiated C57BL/6-Ly5.1^+^ mice were injected with a 50:50 mixture of Ly5.1^+^ WT cells and test Ly5.2^+^ *Mnt*^*fl/fl*^*Rag1Cre* or *Mnt*^*+/+*^*Rag1Cre* bone marrow cells (Fig. 1A). Analysis by flow cytometry after 12 weeks (Supplementary Fig. S1) revealed that the bone marrow cells from *Mnt*^*fl/fl*^*Rag1Cre* mice had competed poorly against WT cells in regenerating lymphoid populations compared to those from *Mnt*^*+/+*^*Rag1Cre* mice. Thymi displayed a significantly lower proportion of Ly5.2^+^ *Mnt*^*fl/fl*^*Rag1Cre* cells (gold bars) than Ly5.2^+^ *Mnt*^*+/+*^*Rag1Cre* cells (brown bars) in all major thymic sub-populations (Fig. 1B). Similarly, the spleen of reconstituted mice contained significantly fewer *Mnt*^*fl/fl*^*Rag1Cre* than *Mnt*^*+/+*^*Rag1Cre* CD4^+^ or CD8^+^ T cells (Fig. 1C). Comparable outcomes were noted for B lineage cells (CD19^+^) in the spleen and bone marrow (Fig. 1C, D). In contrast, as anticipated, Ly5.2^+^ *Mnt*^*+/+*^*Rag1Cre* and Ly5.2^+^ *Mnt*^*fl/fl*^*Rag1Cre* myeloid cells (Mac1^+^), were present in comparable numbers in the spleen and bone marrow in competitively reconstituted mice. We conclude that MNT loss puts both T and B lymphopoiesis at a significant competitive disadvantage.

### Impact of MNT loss on T lymphopoiesis

**Fig. 2:**
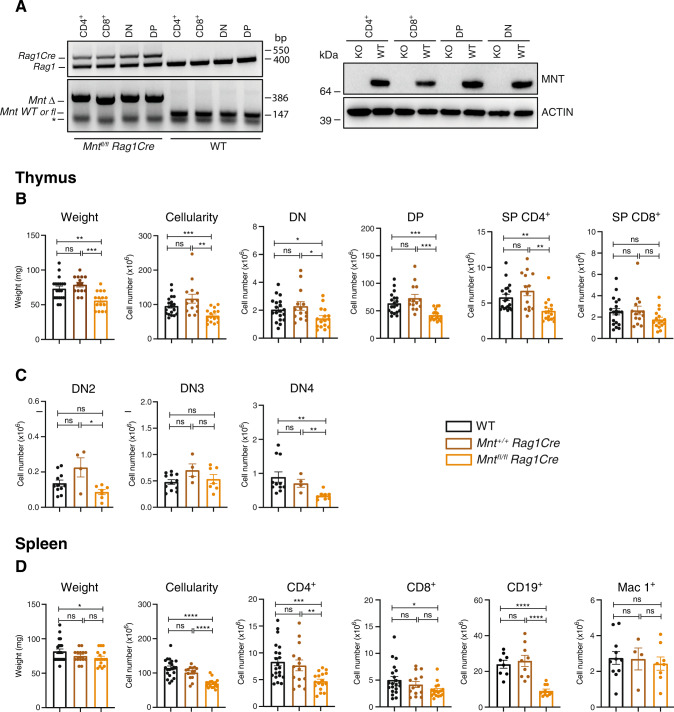
MNT loss impairs normal T cell development. **A** *Mnt* deletion mediated by *Rag1Cre* gene in lymphoid progenitors is highly efficacious. Typical PCR (left) and western blot (right) analysis of the indicated T cell sub-populations sorted from thymi of 6–7 wk-old WT and *Mnt*^*fl/fl*^ *Rag1Cre* (KO) mice. In left panel, *indicates primer dimer. **B** *Mnt*^*fl/fl*^ *Rag1Cre* mice exhibit modest T cell deficit. (L to R) Weight, cellularity and flow cytometric quantification of DN, DP, SP CD4 + and SP CD8^+^ T cells in thymi of 6–7 wk-old mice of indicated genotypes. **C** DN sub-population analysis (see Supplementary Fig. 1) reveals significant reduction of DN4 (CD4^-^CD8^-^CD25^-^CD44^-^) cells in *Mnt*^*fl/fl*^ *Rag1Cre* mice. **D** Splenic T and B cells, but not myeloid cells, are reduced in *Mnt*^*fl/fl*^ *Rag1Cre* mice. Cell preparations from thymi and spleens of 6–7 wk-old mice were analysed by flow cytometry (*n* = 4 to 21); genotypes shown are WT (black), *Mnt*^*+/+*^*Rag1Cre* (brown) and *Mnt*^*fl/fl*^*Rag1Cre* (gold); dots indicate individual mice; bars show mean ± SEM; *P ≤ 0.05, **P ≤ 0.01; ***P ≤ 0.001; ****P ≤ 0.0001, ns not significant.

We have previously investigated the impact of MNT loss in B lymphopoiesis [15]. To investigate how MNT loss affects T lymphopoiesis, we analysed the thymus and spleen in 6–7 wk-old *Mnt*^*fl/fl*^*Rag1Cre*, *Mnt*^+/+^*Rag1Cre* and WT mice. *Mnt* deletion mediated via the *Rag1Cre* transgene was very efficient, as shown by PCR and Western blot analysis (Fig. 2A).

Thymic weight and cellularity were reduced to ~65% of normal in *Mnt*^*fl/fl*^*Rag1Cre* mice, primarily due to fewer DP T cells (*P* ≤ 0.001), although DN and SP CD4^+^ populations were also significantly reduced (Fig. 2B). DN4 (CD25^-^CD44^-^) cells were more affected than DN2 (CD25^+^CD44^+^) or DN3 (CD25^+^CD44^-^) cells (Fig. 2C and Supplementary Fig. S2A). This deficit was not due to a failure of *Τcrβ* gene rearrangement because intracellular TCRβ protein was readily detectable in *Mnt*^*fl/fl*^*Rag1Cre* DN4 cells (Supplementary Fig. S2B).

Spleen cellularity was also reduced in young *Mnt*^*fl/fl*^*Rag1Cre* mice (Fig. 2D), primarily due to decreased B lymphoid cells (~37%; *p* < 0.0001), as reported previously [15]. In addition, T cells were reduced, particularly CD4^+^ T cells (~60%; *p* < 0.001), but the relative proportions of naïve, memory and effector T cells were equivalent between WT and *Mnt*-deficient T cells (Supplementary Fig. S2C, D). Myeloid (Mac1^+^) cellularity was unaffected (Fig. 2D), as expected from the lack of *Rag1Cre* expression in myeloid cells [22].

### MNT loss increases T cell apoptosis

**Fig. 3:**
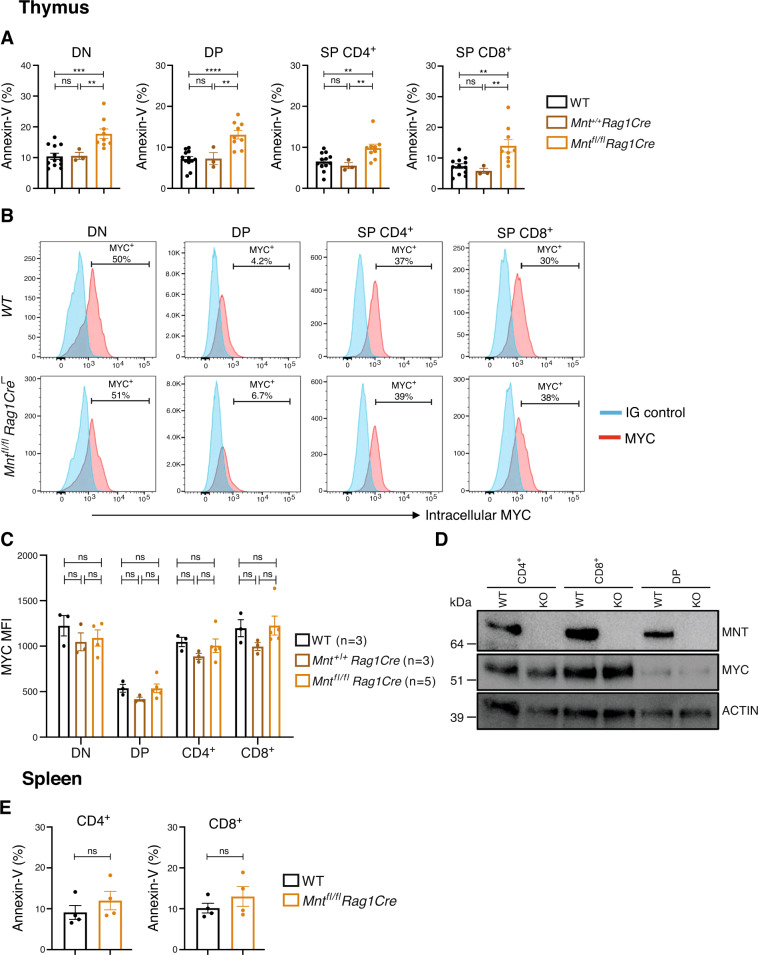
MNT loss enhances T cell apoptosis but does not alter MYC level. **A** MNT loss increases apoptosis in all major thymic T cell sub-populations. **B**–**D** MNT loss does not alter MYC levels in thymic T cell sub-populations. **B** Typical intracellular MYC staining. Thymocytes were stained with antibodies against CD4 and CD8 and then fixed, permeabilised and stained with an antibody against MYC (pink) or Ig isotype-matched control antibody (blue). **C** Mean fluorescence intensity (MFI) for intracellular MYC ± SEM for the indicated thymocyte sub-populations. **D** Typical western blot showing MNT, MYC and ACTIN (protein loading control) protein levels in the indicated thymocyte sub-populations. Note the lower MYC levels in DP cells, a well-documented feature of this predominantly quiescent T lymphoid cell population [57]. **E** MNT loss has minor impact on apoptosis of splenic CD4^+^ and CD8^+^ T cells. Apoptosis was quantified by flow cytometry after staining cells with AnnexinV-FITC. Genotypes analysed were WT (black), *Mnt*^*+/+*^*Rag1Cre* (brown) and *Mnt*^*fl/fl*^*Rag1Cre* (gold). Dots indicate individual mice; bars show mean ± SEM; **P ≤ 0.01; ***P ≤ 0.001; ****P ≤ 0.0001; ns not significant.

The T cell deficit in *Mnt*^*fl/fl*^*Rag1Cre* mice seemed likely to reflect increased apoptosis and/or reduced MYC levels. All four major thymic sub-populations in *Mnt*^*fl/fl*^*Rag1Cre* mice displayed a significantly increased proportion of annexin V-positive cells compared to their WT or *Mnt*^*+*/+^*Rag1Cre* control counterparts (Fig. 3A, Supplementary Fig. S3) and there was a similar trend for CD4^+^ and CD8^+^ T cells in the spleen (Fig. 3E). However, MNT loss did not alter endogenous MYC protein levels in any of these cell populations, as shown by flow cytometric and immunoblot analysis (Fig. 3B–D).

**Fig. 4:**
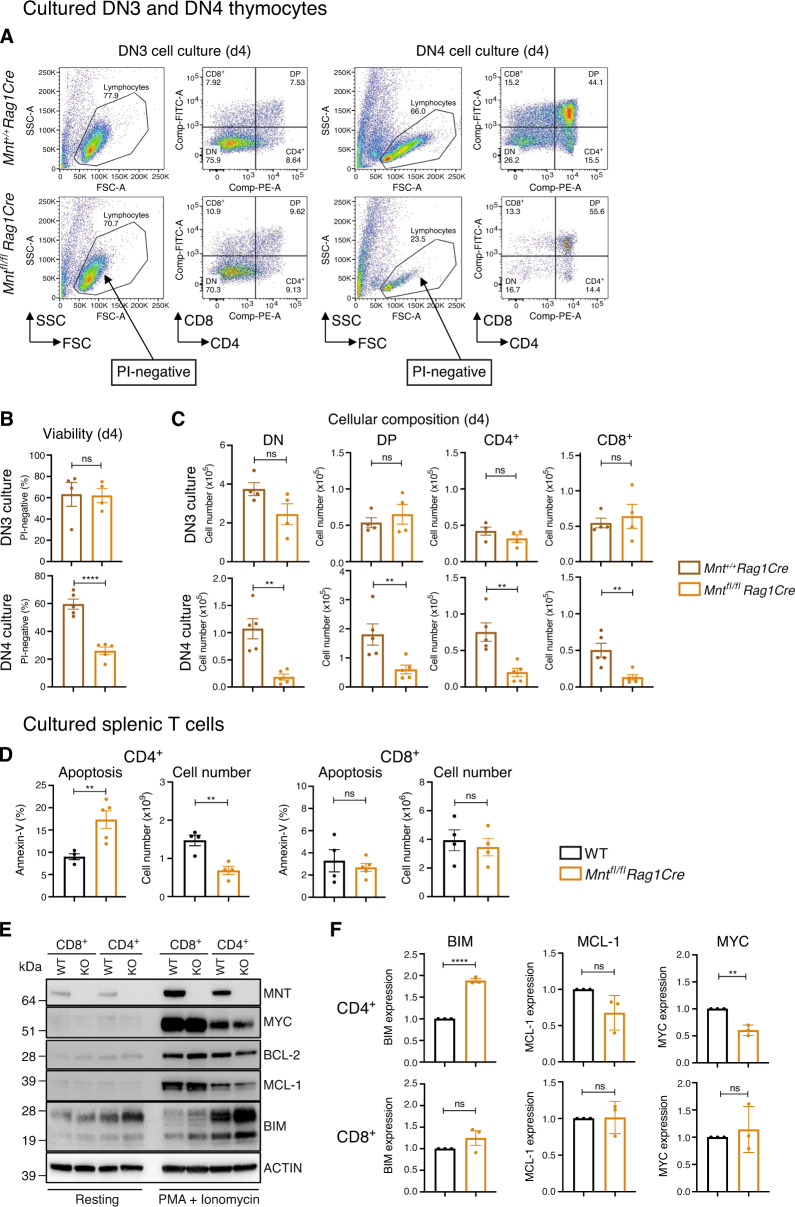
MNT loss increases apoptosis of thymic DN4 and splenic CD4^+^ cells in vitro. **A**–**C** MNT loss enhances predisposition of DN4 but not DN3 progenitor T cells to undergo apoptosis in vitro. DN3 and DN4 cells sorted by FACS from thymi of *Mnt*^*fl/fl*^*Rag1Cre* (gold) and control *Mnt*^*+/+*^*Rag1Cre* (brown) mice were cultured in IL-7 on OP9-DL1 stromal cells and (**A**) analysed on d4 by flow cytometry for (**B**) viability and (**C**) differentiation. **D**–**F** MNT loss enhances apoptosis of stimulated CD4^+^ but not CD8^+^ splenic T cells. CD4^+^ and CD8^+^ T cells sorted from WT and *Mnt*^*fl/fl*^*Rag1Cre* spleens were cultured in 2 mL medium containing 20 ng/mL PMA and 1 μg/mL ionomycin for 72 h and were then analysed by flow cytometry and immunoblot analysis. **D** Annexin V-staining (left panels) and viable cell number (right panels). **E** Typical western blot of MNT, MYC, BCL-2, MCL-1 and BIM protein before and after stimulation, with ACTIN serving as a loading control. **F** Quantification of BIM, MCL-1 and MYC protein in 3 western blots, relative to ACTIN in the same blot, in T lymphoid cells after stimulation. Values for *Mnt*^*fl/fl*^*Rag1Cre* cells (gold) were normalised to those for WT cells (black). Dots indicate individual mice; bars show mean ± SEM; *P ≤ 0.05, **P ≤ 0.01, ****P ≤ 0.0001; ns not significant.

Enhanced apoptosis probably also explains the reduced DN4 population in *Mnt*^*fl/fl*^*Rag1Cre* mice (Fig. 2C). When sorted DN3 and DN4 cells were cultured on OP9-DL1 stromal cells with IL-7, conditions which are permissive for T lymphoid cell proliferation and differentiation (Fig. 4A), MYC levels and proliferation were unaffected (Supplementary Fig. S4A, B). However, the *Mnt* KO DN4 cells produced considerably fewer viable cells, of all differentiation stages, than *Mnt* WT DN4 cells (Fig. 4B, C), while the DN3 cell cultures showed no major differences. Thus, MNT loss apparently renders DN4 cells, but not DN3 cells, more vulnerable to apoptosis during culture.

MNT loss increased apoptosis of splenic CD4^+^ T cells activated in vitro by PMA and ionomycin. The proportion of annexin-V-positive cells was ~2-fold higher in the *Mnt* KO than the *Mnt*^+/+^ CD4^+^ T cell population and there were fewer viable cells (Fig. 4D, Supplementary Fig. S4C). In contrast, MNT loss had little consequence for CD8^+^ T cells under these conditions.

Taken together, these observations suggest that increased apoptosis is the major determinant of the T cell deficit in *Mnt*^*fl/fl*^*Rag1Cre* mice.

### BIM is a critical mediator of apoptosis in MNT-null T cells

Cellular stress causes cell death via the mitochondrial apoptosis pathway, which is regulated by opposing factions of the BCL-2 family [27, 28] and extensive genetic studies have identified BIM (BCL2L11), a pro-apoptotic BH3-only protein, as a key trigger of lymphocyte death [29–32]. We therefore hypothesised that BIM contributed to the enhanced apoptosis of MNT-deficient T cells.

**Fig. 5:**
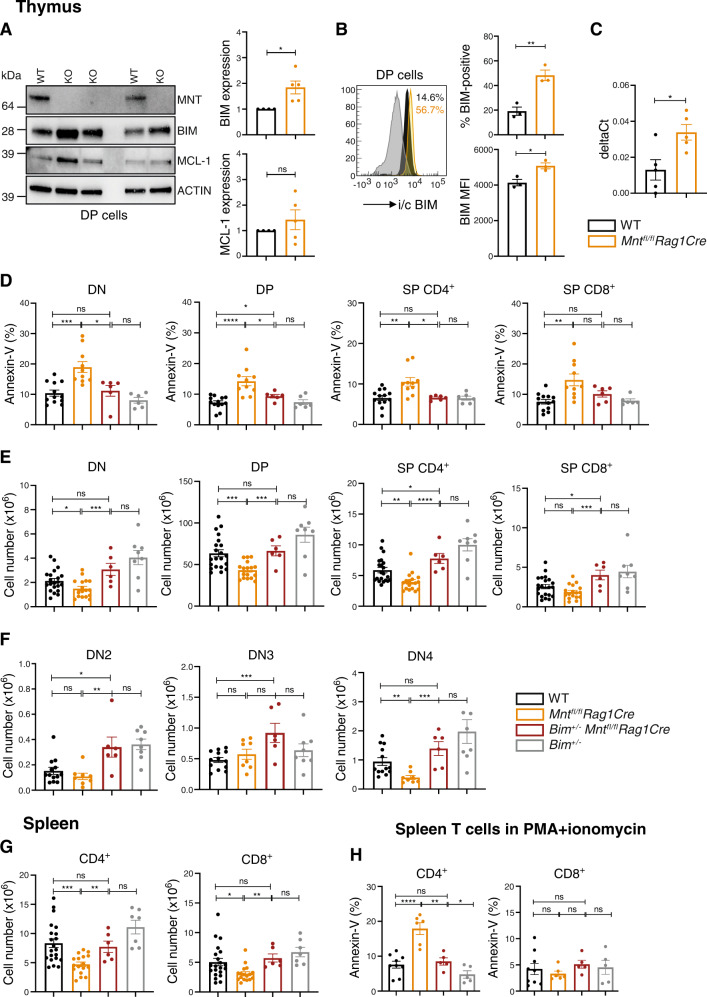
Elevated BIM promotes apoptosis in *Mnt* KO T cells. **A**, **B** BIM protein is elevated in *Mnt* KO DP thymocytes. DP T cells were sorted from thymi of 3 WT and 5 *Mnt*^*fl/fl*^ *Rag1Cre* mice and BIM levels were quantified by immunoblot and intracellular FACS analysis. **A** Left panel shows one of two western blots and right panel quantifies BIM and MCL-1 levels for both blots relative to ACTIN (protein loading control) and normalised to that in WT cells. **B** Left panel shows a typical FACS profile, comparing BIM level in DP thymocytes from *Mnt*^*fl/fl*^*Rag1Cre* (gold), WT (black) and, as a negative control, *Bim*^*−*^^/−^ mice (grey). Right panels show % BIM-positive cells and Mean Fluorescence Intensity (MFI) for BIM in three independent experiments ± SEM. **C** *Bim* transcription is elevated in *Mnt* KO DP thymocytes. Quantitative RT-PCR of *Bim* transcripts in *Mnt*^*fl/fl*^ *Rag1Cre* (gold) compared to WT (black) DP thymocytes. Values were calculated relative to *Gapdh* control. **D** *Bim* heterozygosity reduces apoptosis of the major thymic T cell sub-populations in *Mnt*^*fl/fl*^*Rag1Cre* mice. **E** *Bim* heterozygosity restores cellularity of major thymic T cell sub-populations in *Mnt*^*fl/fl*^*Rag1Cre* mice. **F** *Bim* heterozygosity increases the cellularity of DN2, DN3 and DN4 progenitor cell populations in the thymus of *Mnt*^*fl/fl*^*Rag1Cre* mice. **G** *Bim* heterozygosity increases CD4^+^ and CD8^+^ T cells in the spleen of *Mnt*^*fl/fl*^ *Rag1Cre* mice. **H** *Bim* heterozygosity prevents increased apoptosis in activated CD4^+^ and CD8^+^ splenic T cells caused by MNT loss. Splenic CD4^+^ and CD8^+^ T cells from mice of the indicated genotypes were cultured in PMA + ionomycin for 72 h. Apoptosis was quantified by flow cytometry after staining cells with Annexin-V-FITC. Genotypes analysed include WT (black), *Mnt*^*fl/fl*^*Rag1Cre* (gold), *Bim*^+/−^*Mnt*^*fl/fl*^ *Rag1Cre* (rust) and *Bim*^+/−^ (grey). Dots indicate individual mice; bars show mean ± SEM; *P ≤ 0.05, **P ≤ 0.01; ***P ≤ 0.001; ****P ≤ 0.0001; ns not significant. Data include mice in Fig. 2 plus additional mice.

Notably, western blot analysis and intracellular flow cytometry revealed increased BIM protein in *Mnt* KO DP thymocytes compared to WT DP thymocytes but no significant change in anti-apoptotic MCL-1, an important regulator of T cell survival [33] (Fig. 5A, B). A modest increase in *Bim* transcription in MNT-deficient T cells (Fig. 5C) may partly account for the increased BIM protein.

BIM protein was also notably higher in mitogen-activated *Mnt* KO CD4^+^ splenic T cells, but not in activated CD8^+^ T cells (see Fig. 4E, F), paralleling their apoptosis susceptibility under these conditions (Fig. 4D). These results suggest that MNT suppresses *Bim* expression in T lymphoid cells, as we previously proposed for B lymphoid cells [15].

To directly test the importance of BIM in the apoptosis of MNT-deficient T cells, we bred *Bim*^+/−^ *Mnt*^*fl/fl*^*Rag1Cre* mice (*Bim* is functionally haplo-insufficient [34]). Indeed, apoptosis in thymocyte populations from *Bim*^+/−^ *Mnt*^*fl/fl*^*Rag1Cre* mice (rust bars) was significantly less than in those from *Mnt*^*fl/fl*^ *Rag1Cre* mice (gold bars), and comparable to that in WT mice (black bars) (Fig. 5D). Notably, the cellularity of the major thymic sub-populations was restored (Fig. 5E), as was that of the DN4 sub-population (Fig. 5F). Splenic T cell cellularity was also restored to normal in the *Bim*^+/−^ *Mnt*^*fl/fl*^*Rag1Cre* mice (Fig. 5G). Furthermore, loss of one *Bim* allele prevented the enhanced apoptosis of *Mnt*^*fl/fl*^*Rag1Cre* CD4^+^ splenic T cells stimulated in vitro by PMA + ionomycin (Fig. 5H). In summary, MNT loss upregulates BIM, thereby enhancing the vulnerability of T cells to apoptosis during normal T lymphopoiesis.

MNT also constrains BIM levels in other cell types. Thus, using CRISPR/Cas9, we found that human *MNT* KO HEK 293 T and HeLa cells, and *Mnt* KO *Bax*^−/−^*Bak*^−/−^ mouse embryonic fibroblasts (MEFs) express more BIM protein than their parental cells (Supplementary Fig. S5). Since apoptosis is blocked in *Bax*^−/−^*Bak*^−/−^ MEFs, MNT regulation of BIM levels must occur upstream of mitochondrial permeability changes. Of note, the mechanism is reversible, because when the MEFs (*Mnt*^+/+^ or *Mnt*^−/−^) cells were infected with *MntERT2* retrovirus and treated with 4-OHT to activate the exogenous MNTERT2 protein [35], BIM levels were again reduced (Supplementary Fig. S5I).

### *Mnt* deletion prevents T lymphoma development in *MYC10*^*hom*^ transgenic mice

To examine the impact of MNT loss on MYC-driven T lymphomagenesis, we utilised our *MYC10*^*hom*^ mice [24, 36], which are homozygous for a transgene expressing human *MYC* cDNA via the pan-haemopoietic *VavP* transgenic vector [37]. In these mice, expression of transgenic MYC protein in T lymphoid cells is significantly higher than in B lymphoid and myeloid cells, and thymic T lymphoma is the major cause of morbidity, although these mice can also develop disseminated histiocytic myeloid (monocyte/macrophage) (Mac1^+^F4/80^+^Gr1^-^) tumours affecting the spleen and other organs [36].

**Fig. 6:**
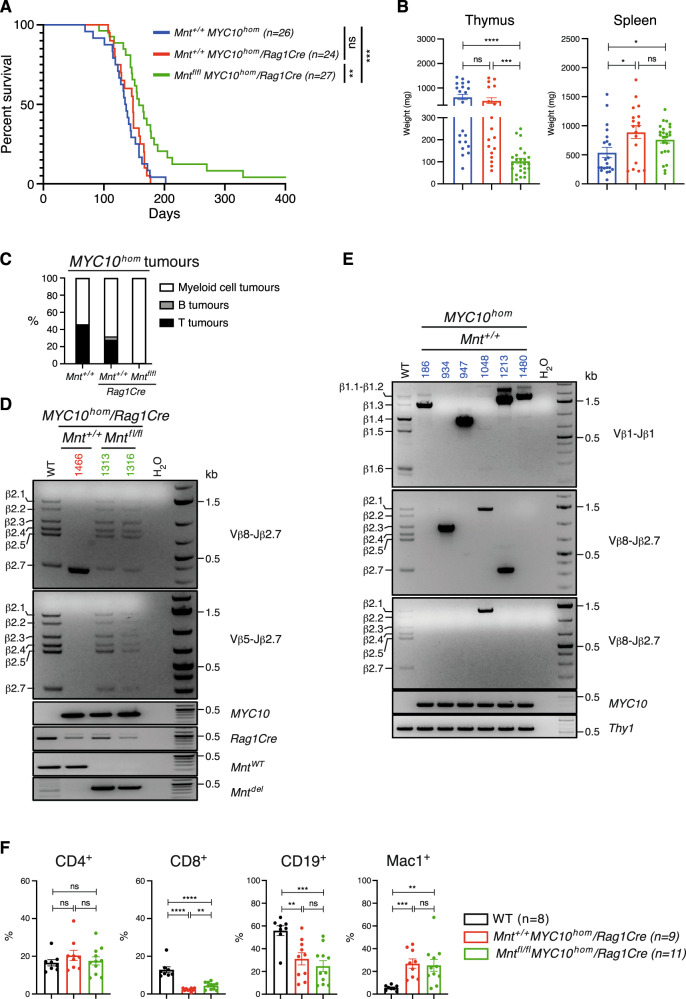
Lymphoid-specific MNT loss prevents thymic lymphoma development in *Mnt*^*fl/fl*^*MYC10*^*hom*^*/Rag1Cre* mice. **A** Kaplan–Meier survival curves showing significantly delayed morbidity for *Mnt*^*fl/fl*^*MYC10*^*hom*^*/Rag1Cre* mice (green curve; median survival 165 d; *n* = 27: 16 F, 11 M) compared to control *Mnt*^*+/+*^*MYC10*^*hom*^ (blue curve; median survival 134 d; *n* = 26: 17 F, 9 M) and *Mnt*^*+/+*^*MYC10*^*hom*^*/Rag1Cre* (red curve; median survival 148 d; *n* = 24: 12 F, 12 M) mice. **B** MNT loss prevents thymus but not spleen enlargement. Sick mice were euthanised and the weights of spleens and thymi measured. Bars show mean thymus and spleen weights ± SEM, and dots indicate values for individual euthanised mice. **C** MNT loss prevents T lymphoma development. Proportion of T (black) and B (grey) thymic lymphomas and myeloid tumours (white) in euthanised *Mnt*^*+/+*^*MYC10*^*hom*^, *Mnt*^*+/+*^*MYC10*^*hom*^*/Rag1Cre* and *Mnt*^*fl/fl*^*MYC10*^*hom*^*/Rag1Cre* mice. (See text and Tables 1–3). **D** Polyclonality of thymic T cells in *Mnt*^*fl/fl*^*MYC10*^*hom*^*/Rag1Cre* mice. PCR analysis of *Tcrβ* gene rearrangements was performed on DNA isolated from DP thymic cells from *Mnt*^*fl/fl*^*MYC10*^*hom*^*/Rag1Cre* mice # 1313, #1316 (green) and, as a control, a clonal T lymphoma *Mnt*^*+/+*^*MYC10*^*hom/*^*Rag1Cre* mouse #1466 (red). Lower 4 panels show PCR genotype analysis. **E** Clonality of thymic T lymphomas from *Mnt*^*+/+*^*MYC10*^*hom*^ mice. DNA was isolated from individual T lymphomas (blue) and genomic PCR analysis performed for indicated *Tcrβ* gene rearrangements, *MYC10* transgene and, as a loading control, *Thy1*. **F** Enlarged spleens from both *Mnt*^*+/+*^ and *Mnt*^*fl/fl*^ *MYC10*^*hom*^*/Rag1Cre* mice have elevated proportion of Mac1^+^ myeloid cells. Mouse genotypes analysed included WT (black), *Mnt*^*+/+*^*MYC10*^*hom*^*/Rag1Cre* (red) and *Mnt*^*fl/fl*^*MYC10*^*hom*^*/Rag1Cre* mice (green). Dots indicate individual mice; bars show mean % cellularity ± SEM; *P ≤ 0.05, **P ≤ 0.01; ***P ≤ 0.001; ****P ≤ 0.0001; ns not significant.

*Mnt*^*fl/fl*^*MYC10*^*hom*^/*Rag1Cre* mice survived significantly longer than the control *Mnt*^*+/+*^*MYC10*^*hom*^ and *Mnt*^*+/+*^*MYC10*^*hom*^/*Rag1Cre* mice (median of 158 d compared to 136 d and 148 d; *p* ≤ 0.001, *p* ≤ 0.01, respectively) (Fig. 6A) and autopsy of euthanised sick mice revealed a major difference in pathology. Whereas the control mice presented with massively enlarged thymi and/or splenomegaly, *Mnt*^*fl/fl*^*MYC10*^*hom*^/*Rag1Cre* mice presented with splenomegaly but not thymic enlargement (Fig. 6B).

Importantly, *Rag1Cre*-mediated *Mnt* deletion specifically prevented T lymphoma development in *MYC10*^*hom*^ mice (Fig. 6C). None of the 26 mice in the *Mnt*^*fl/fl*^ *MYC10*^*hom*^*/Rag1Cre* cohort developed thymic T lymphomas (Supplementary Table S3) and, where analysed, their thymic T cells had polyclonal rather than monoclonal TCR gene rearrangement, consistent with not being transformed (eg # 1313 and #1316 in Fig. 6D). In contrast, 12/26 *Mnt*^+/+^*MYC10*^*hom*^ and 7/24 *Mnt*^*+/+*^*MYC10*^*hom*^*/Rag1Cre* control mice developed massive thymi (up to 1440 mg) and 14/15 of those immunophenotyped were CD4^+^CD8^+^ T lymphomas (the other being a CD19^+^ B lymphoma) (Supplementary Fig. S6A–C and Tables S1, S2). Seven of these thymic T lymphomas analysed by PCR showed 1 or 2 dominant *Tcrβ* gene rearrangements, indicative of mono- or bi- clonality (Fig. 6D, E). Curiously, the T lymphomas were also Mac-1-positive (Supplementary Fig. S6A, B), which may be due to high MYC expression, because activated CD8^+^ T cells express Mac1 [38].

The grossly enlarged spleens arising in either *Mnt*^+/+^ or *Mnt* KO *MYC10*^hom^ mice contained a high proportion of transplantable Mac-1^+^ myeloid cells (Fig. 6F, Supplementary Table S4). Histological review revealed invasion of many other tissues by these tumour cells, as described previously [36]. Although the splenic CD4^+^ T cells in these mice were clearly activated (CD44^+^CD62L^-^) (Supplementary Fig. S6D), they were not transplantable (Supplementary Table S4).

In summary, lymphoid cell-specific *Mnt* deletion prevented the development of MYC-driven thymic T lymphomas in *MYC10*^*hom*^ transgenic mice but not their myelomonocytic tumours. Whether the MYC-driven myeloid tumorigenesis requires MNT is not addressed by these studies as *Rag1Cre* is expressed only in lymphoid progenitors [39].

### MNT loss impairs T cell development in *MYC10*^*hom*^ transgenic mice

**Fig. 7:**
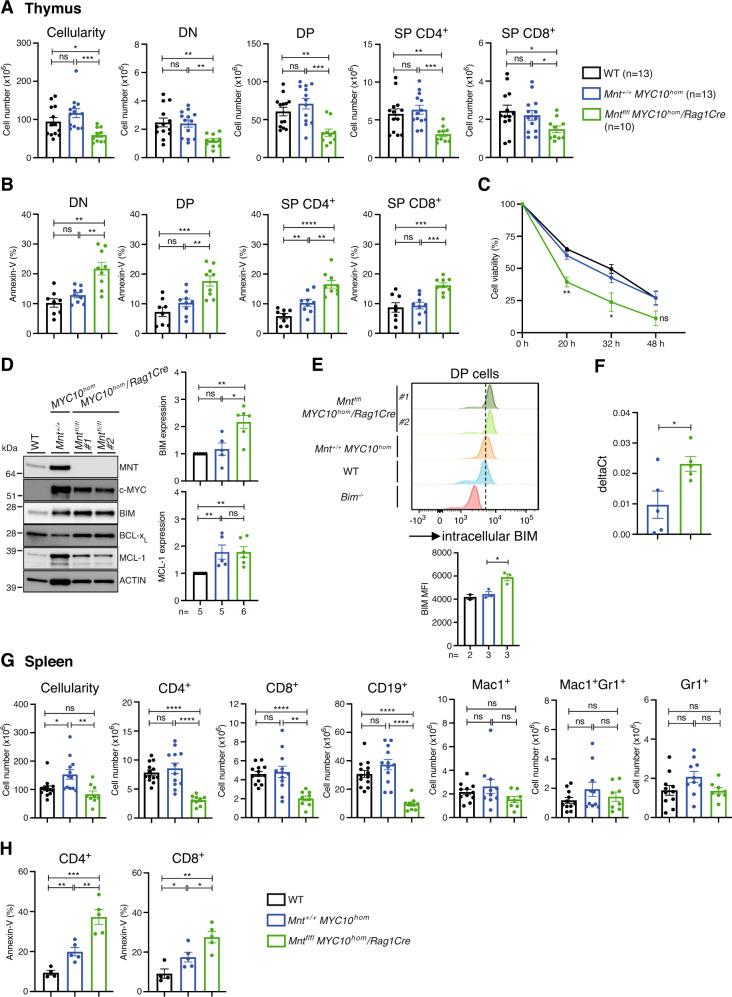
MNT loss impairs T cell development in pre-malignant *MYC10*^*h*om^ transgenic mice. **A**–**E** Thymus: **A** Reduction in cellularity of all major thymic T cell sub-populations in 8 wk-old premalignant *Mnt*^*fl/fl*^*MYC10*^*hom*^*/Rag1Cre* mice (green; *n* = 10: 3 F, 7 M) *vs* premalignant *Mnt*^*+/+*^*MYC10*^*hom*^ (blue; *n* = 13: 8 F, 5 M) transgenic mice and control WT (black; *n* = 13: 7 F, 6 M) mice. Cell number was determined by flow cytometry after immunostaining. **B**, **C** Elevated apoptosis of thymic T cells in premalignant *MYC10*^*h*om^ transgenic mice was further enhanced by MNT loss. **B** Quantification of AnnexinV-positive cells in the four major thymic T cell sub-populations from WT (black; *n* = 8: 3 F, 5 M), *Mnt*^*+/+*^*MYC10*^*hom*^ (blue; *n* = 9: 6 F, 3 M) and *Mnt*^*fl/fl*^*MYC10*^*hom*^*/Rag1Cre* (green; *n* = 9: 3 F, 6 M) mice. **C** MNT loss enhances death of premalignant *MYC10*^*hom*^ DP thymic T cells in vitro. Sorted DP T cells were cultured at 37 ^o^C in OptiMEM medium + 10% FCS and viability determined by PI staining at 0, 20, 32 and 48 h. Values are expressed relative to viability at t = 0 h. **D**–**F** MNT loss is associated with elevated BIM expression. **D** Left panel. Typical Western blot showing elevated BIM levels in sorted DP thymic T cells from 2 independent *Mnt*^*fl/fl*^*MYC10*^*hom*^*/Rag1Cre* mice vs control *Mnt*^*+/+*^*MYC10*^*hom*^ or WT mice. Right panel shows quantification of BIM and MCL-1 protein in 2 independent western blots, normalised to ACTIN (protein loading control) first and then normalised to expression in WT cells (see Supplementary Fig. S7A for MNT, MYC and BCL-X_L_ quantification). **E** Elevated intracellular BIM staining in DP thymic T cells from *Mnt*^*fl/fl*^*MYC10*^*hom*^*/Rag1Cre vs* control *Mnt*^*+/+*^*MYC10*^*hom*^ mice. Upper panel shows a typical FACS analysis of intracellular BIM expression and lower panel presents BIM Mean Fluorescence Intensity (MFI) ± SEM for DP thymic T cells from 2 independent WT and 3 independent *Mnt*^*+/+*^*MYC10*^*hom*^ and *Mnt*^*fl/fl*^*MYC10*^*hom*^*/Rag1Cre* mice. DP thymic T cells from *Bim*^*−/−*^ mice served as a negative control. **F** Elevated *Bim* transcripts in DP thymic T cells from *Mnt*^*fl/fl*^*MYC10*^*hom*^*/Rag1Cre vs* control *Mnt*^*+/+*^*MYC10*^*hom*^ mice revealed by quantitative RT-PCR. Values were calculated relative to *Gapdh* mRNA used as a loading control. **G**, **H** Spleen: **G** Marked reduction of T and B lymphoid but not myeloid cells in spleens of 8 wk-old premalignant MNT-null *MYC10*^*h*om^ transgenic mice. **H** Elevated apoptosis of splenic CD4^+^ and CD8^+^ T cells in MNT-null *MYC10*^*h*om^ transgenic mice, quantified by Annexin-V staining. Dots indicate individual mice. Mice used for **G**, **H** were those analysed in **A** and **B** above. Bars show mean ± SEM; *P ≤ 0.05, **P ≤ 0.01; ***P ≤ 0.001, ****P ≤ 0.0001, ns not significant.

To clarify why T lymphomagenesis was abrogated in *Mnt*^*fl/fl*^*MYC10*^*hom*^*/Rag1Cre* mice, we analysed healthy young ie premalignant (8 wk-old) mice. PCR and western blot analysis of DP thymocytes confirmed efficient *Mnt* deletion (not shown**)**. Of note, thymic cellularity was reduced ~50% in *Mnt*^*fl/fl*^*MYC10*^*hom*^/*Rag1Cre* mice (green) compared to control *Mnt*^*+*/+^*MYC10*^*hom*^ mice (blue) (*p* ≤ 0.001), and all thymocyte sub-populations were reduced around two-fold (Fig. 7A).

As reported previously [36], the level of MYC protein in thymocytes of *MYC10*^*hom*^ transgenic mice greatly exceeds endogenous MYC levels (compare first 2 tracks in Fig. 7D). Concomitantly, MNT levels are also elevated 3-fold (Fig. 7D, Supplementary Fig. S7A).

MNT loss did not affect MYC protein level or cell size in pre-malignant *MYC10*^hom^ T cells (Supplementary Fig. S7B–D). However, the proportion of annexin-V-positive cells was significantly higher in *Mnt* KO *MYC10*^hom^ than *Mnt*^+/+^ *MYC10*^hom^ thymocyte sub-populations (compare green to blue bars), which in turn tended to be higher than comparable WT sub-populations (compare blue to black bars; Fig. 7B). Furthermore, when cultured in vitro, *Mnt* KO *MYC10*^hom^ DP thymocytes died faster than their *Mnt*^+/+^*MYC10*^hom^ or WT counterparts (Fig. 7C). Thus, an overt consequence of MNT loss was increased apoptosis.

The increased apoptosis paralleled elevated BIM protein levels (Fig. 7D, E) and increased *Bim* transcription (Fig. 7F) in thymic DP T cells. Anti-apoptotic MCL-1 protein levels were higher in *MYC10*^hom^ than WT DP thymocytes, but not affected by MNT loss (Fig. 7D). BCL-X_L_ levels were comparable in cells from all three genotypes (Supplementary Fig. S7A). The tumour suppressor p53, which can activate apoptosis via transcriptional induction of pro-apoptotic BH3-only proteins PUMA and NOXA [40], was not detectable, by either western blot or qRT-PCR analyses (not shown).

MNT loss also resulted in a deficit of CD4^+^ and CD8^+^ T cells in the spleen of pre-malignant *MYC10*^hom^ mice (Fig. 7G, green bars), and annexin V staining (Fig. 7H) indicated greater predisposition to apoptosis. Pertinently, the high MYC levels did not further increase in the absence of MNT (Supplementary Fig. S8A, B). Staining for CD44 and CD62L indicated that, as expected, the *MYC* transgenic splenic T cells were enlarged and highly activated (Supplementary Fig. S8C, D).

MNT loss also greatly reduced CD19^+^ B lymphoid cells in the spleen and bone marrow of the young *MYC10*^hom^ mice, as reported previously for *Mnt*^*fl/fl*^ *Eμ-Myc/Rag1Cre* mice [15], but myeloid cells (Mac1^+^, Gr1^+^, Mac1^+^Gr1^+^) were unaffected (Fig. 7G, Supplementary Fig. S9C). Indeed, myeloid cell numbers were still normal at this age in both *MYC10*^hom^ genotypes, despite the disseminated myeloid disease that inevitably develops as these mice age.

We conclude that abrogation of thymic T lymphoma development in *Mnt* KO *MYC10*^*hom*^ mice is largely due to the increased apoptosis of the highly proliferative pre-malignant thymic T cells, driven by elevated BIM levels.

### MNT loss prevents γ-radiation-induced T lymphoma development

To investigate the MNT-dependency of T lymphomagenesis in the absence of a *Myc* transgene, we performed serial total body γ-irradiation of C57BL/6 mice. In this well-studied model [26, 41], γ-irradiation decimates leukocytes and the thymus is repeatedly regenerated from bone marrow-derived haemopoietic stem/progenitor cells, some of which have sustained γ-irradiation-induced oncogenic mutation.

**Fig. 8:**
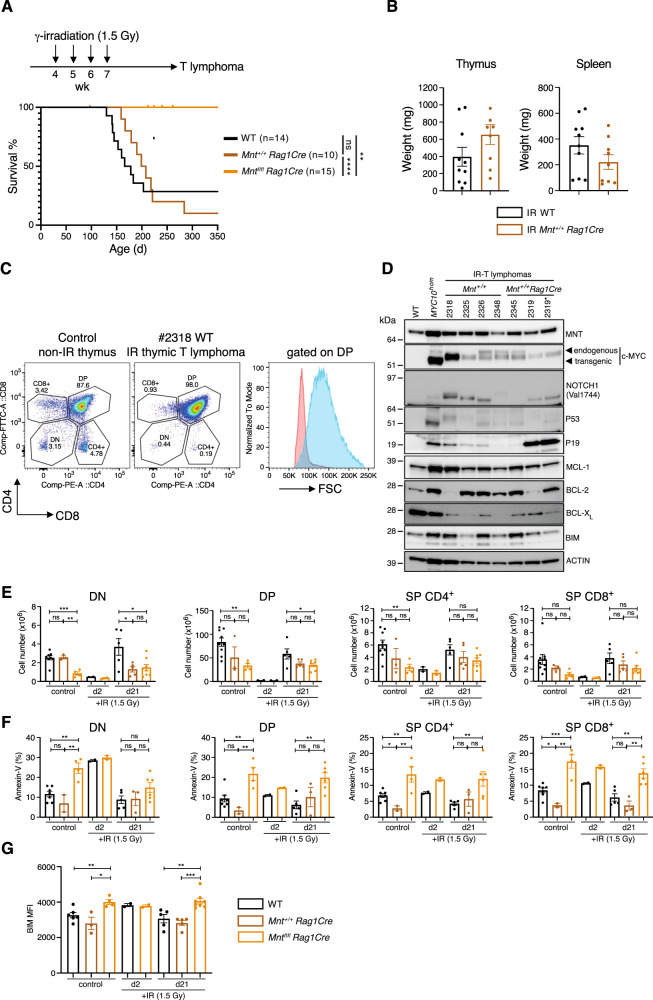
Lymphoid-specific MNT loss prevents T lymphomagenesis induced in C57BL/6 mice by fractionated γ-irradiation. **A** Kaplan–Meier survival curves for γ-irradiated WT (*n* = 14: 11 F, 3 M), *Mnt*^*+/+*^ *Rag1Cre* (*n* = 10: 6 F, 4 M) and *Mnt*^*fl/fl*^ *Rag1Cre* (*n* = 15: 9 F, 6 M) C57BL/6 mice. Starting at 33 days of age, mice of the indicated genotypes received 4 doses of 1.5 Gy γ-irradiation at weekly intervals. In contrast to the control genotypes, all γ-irradiated *Mnt*^*fl/fl*^ *Rag1Cre* remained healthy for at least 250 days of age. **B** The γ-irradiated WT and *Mnt*^*+/+*^ *Rag1Cre* mice developed an enlarged thymus and/or spleen. By comparison, the mean weight of the thymus and spleen from healthy unirradiated 8 wk-old C57BL/6 mice is 68 ± 3.4 mg and 87 ± 4.1 mg respectively. **C** Tumours were dominated by large CD4^+^CD8^+^ and/or CD8^+^ T lymphoid cells (see Supplementary Table 5). Flow cytometric analysis comparing enlarged thymus of a γ-irradiated WT mouse to that of a non-irradiated WT mouse. First two panels display cell surface markers in all major thymic sub-populations; the third panel compares size of DP T cells in tumour (blue) with those in a non-irradiated control mouse (pink). **D** Western blot analysis of T lymphomas that developed in control γ-irradiated *Mnt*^+/+^ and *Mnt*^+/+^ *Rag1Cre* mice. Mouse #2319 had a mixed DP (CD4^+^CD8^+^) (designated 2319) and SP (CD8^+^) (designated 2319*) thymic T lymphoma. Mutations that inactivate p53 result in elevated p19Arf protein, due to the absence of the p53-mediated negative feedback loop [58]. ACTIN levels serve as a protein loading control. **E**–**G** Thymic T cell analysis following a single dose of γ-irradiation at 33 days of age. **E**, **F** Cell number (**E**) and apoptosis (**F**) in major thymic T cell sub-populations, analysed by flow cytometry on d2 (WT *n* = 2 (2 M); *Mnt*^*fl/fl*^ *Rag1Cre* *n* = 2 (1 F, 1 M) and d21 (WT *n* = 5 (3 F, 2 M); *Mnt*^+/+^ *Rag1Cre* *n* = 5 (2 F, 3 M); *Mnt*^*fl/fl*^ *Rag1Cre* *n* = 8 (4 F, 4 M) after irradiation. Unirradiated control mice (WT *n* = 9 (3 F, 6 M); *Mnt*^+/+^ *Rag1Cre* *n* = 3 (2 F, 1 M); *Mnt*^*fl/fl*^ *Rag1Cre* *n* = 4 (2 F, 2 M) were analysed on d21. Apoptosis was determined by staining with Annexin-V. **G** MNT loss increases BIM protein levels in γ-irradiated (and non-irradiated) thymic T cells. BIM levels in CD4^+^CD8^+^ thymic T cells obtained from mice analysed in E, F above were quantified by intracellular staining followed by FACS. Each dot represents data from an individual mouse.

As expected, almost all γ-irradiated WT mice and *Mnt*^*+/+*^*/Rag1Cre* controls developed thymic T lymphomas (median survival 172 and 204 d respectively). These usually presented in the thymus and often also in the spleen, and had either a CD4^+^CD8^+^, CD8^+^ or mixed surface marker expression profile (Fig. 8A–C, Supplementary Table S5). Western blot analysis (Fig. 8D) showed that, with one exception, MYC protein was lower in γ-irradiation-induced T lymphomas than in *MYC10*^hom^ transgenic T lymphomas, but nevertheless still far higher than in a normal thymus. NOTCH1 and p53/p19Arf pathway mutations were frequent, as reported previously [42], and all lymphomas expressed MNT and BIM.

Remarkably, however, none of the γ-irradiated *Mnt*^*fl/fl*^*/Rag1Cre* mice developed lymphomas (Fig. 8A). To understand why, we analysed thymi recovering from the first γ-irradiation dose (Fig. 8E–G). All major T cell populations were greatly reduced on d2 compared to unirradiated controls but had largely recovered by d21, irrespective of genotype (Fig. 8E). However, the *Mnt* KO T cells exhibited greater levels of apoptosis compared to controls (Fig. 8F) and intracellular staining revealed significantly elevated BIM (Fig. 8G). We infer that MNT loss ‘enhances BIM-induced apoptosis of cells repopulating the thymus, including any clones expanding from stem cells carrying irradiation-induced oncogenic mutations. Consequently, lymphoma development is prevented.

## Discussion

Using *Rag1Cre*-mediated deletion of *Mnt* in immature lymphoid progenitor cells in otherwise normal young mice, and competitive bone marrow reconstitution of lethally irradiated mice, we have shown that MNT-deficient lymphoid cells are more vulnerable to apoptosis during their development than those expressing MNT ([15], this paper). MNT loss elevated apoptosis and reduced cellularity in the thymus, bone marrow and spleen. In the T lineage, the phenotype affected all major sub-populations and was apparent as early as the DN4 pro-T cell stage, during which pre-TCR and Notch-1 signalling elevates c-MYC expression [43, 44]. In the B lineage, pro-B, pre-B and B cells were all affected [15].

The T cell phenotype of *Mnt*^*fl/fl*^ *Rag1Cre* mice was milder than that reported previously for *Mnt*^fl/fl^ *LckCre* mice [19], which had severe T cell loss, progressive inflammatory disease and late onset T lymphoma. The phenotypic differences may be due to continuous (*Lck* promoter-driven) *vs* transient (*Rag1* promoter-driven) CRE expression. Continuous CRE exposure greatly perturbs T cell development [45], presumably due to accumulated DNA breaks, and this phenotype may be exacerbated by concomitant loss of MNT.

We showed that MNT loss in T cells provoked upregulation of BIM protein, a major initiator of apoptosis [29–32], and that the enhanced apoptosis and T cell deficit were largely prevented by loss of a single *Bim* allele. Both BIM protein and *Bim* mRNA levels were elevated in T cells of *Mnt*^*fl/fl*^ *Rag1Cre* mice. MNT may directly suppress *Bim* transcription, as MNT binding sites in the *Bim* locus have been identified in mouse B cells (see CUT&RUN data GSE132967 reported by Mathsyaraja et al. [46]). Indirect mechanisms may also be involved, but these do not appear to involve either ERK phosphorylation [47] or down-regulation of microR*17-92* [48, 49] (data not shown).

Our data analysing MNT KO HEK 293 T, HeLa and MEF cell lines (Figure S5) suggest that MNT can also dampen BIM expression in non-lymphoid cell types. Importantly, since expression of exogenous MNT in *Mnt* KO cells reduced the elevated BIM levels, the mechanism is reversible. Furthermore, since the MEFs used in this experiment lacked the apoptosis effectors BAX and BAK, MNT-mediated modulation of BIM levels must occur upstream of mitochondrial outer membrane permeability (MOMP) changes.

To gauge how MNT affects T lymphomagenesis, we first used VavP-*MYC10*^hom^ transgenic mice [24, 36], which are prone to both thymic T lymphomas and disseminated myeloid tumours. Strikingly, lymphoid-specific *Mnt* loss abrogated T lymphoma development in the *MYC10*^*hom*^ transgenic mice. None of the *Mnt*^*fl/fl*^ *MYC10*^hom^*/Rag1Cre* cohort (*n* = 26) developed thymic lymphomas and morbidity was solely due to myeloid tumours.

To explore why T lymphomas failed to develop, we compared phenotypes of young mice, prior to any sign of emerging malignancy. MNT loss halved T cell numbers in both the thymus and spleen, and the *Mnt* KO *MYC10*^hom^ T cells were significantly more susceptible to apoptosis than *Mnt*^+/+^*MYC10*^hom^ T cells at all major stages of development. Indeed, their apoptosis is likely greater than suggested by annexin-V labelling, as apoptotic cells are rapidly engulfed by phagocytes in vivo [50]. These results showing the MNT-dependence of VavP-*MYC* transgene-driven T lymphomagenesis confirm and extend those reported by Hurlin’s group for transgenic mice engineered for T cell-specific (*Lck-Cre*-dependent) expression of a stable mutant MYC^T58A^ protein from within the ROSA 26 locus [20, 51].

Importantly, we also showed that the MNT-dependency of T lymphomagenesis extends beyond a *Myc* transgenic setting. Irradiation-induction of T lymphomas, where NOTCH 1 and p53 mutations are principal oncogenic drivers [42], was totally prevented by *Rag1Cre*-mediated *Mnt* deletion.

Quantification of major apoptosis regulators revealed that pro-apoptotic BIM protein was elevated in *Mnt* KO as compared to *Mnt*^*+/+*^ DP thymocytes in healthy young *MYC10*^hom^ transgenic mice. Similarly, apoptosis and BIM protein levels were elevated in DP thymocytes of regenerating thymi from irradiated *Mnt*^*fl/fl*^ *Rag1Cre* versus irradiated *Mnt*^*+/+*^ *Rag1Cre* or WT C57BL/6 mice. We infer that BIM upregulation after *Mnt* deletion is a major factor triggering apoptosis of proliferating T cell populations. Our data suggest that the mechanism is reversible and takes place upstream of BAX/BAK activation and MOMP. Further investigations of mechanism are planned, using RNA-Seq and CUT&TAG.

As a MYC antagonist, MNT was originally considered a tumour suppressor and this was supported by an early study showing that mice with mammary-specific *Mnt* deletion developed mammary adenocarcinoma [23]. The tumour suppressor role received further support when deletions involving the *MNT* gene locus (usually monoallelic) were noted in ~10% of cancers in The Human Cancer Genome Atlas [4], including certain cases of chronic lymphocytic leukaemia [52], a B cell malignancy, and Sezary syndrome, a cutaneous T-cell lymphoma/leukaemia [53]. However, *MNT* is localised on human chromosome 17p13.3 [12], near the potent tumour suppressor gene *TP53* (17p13.1), making it difficult to ascribe any impact of large deletions solely, if at all, to *MNT* deletion.

While MNT might act as a tumour suppressor in certain settings, genetic studies from ourselves and others ([15, 20, 21] and this paper), using four independent mouse models, demonstrate unequivocally that MNT *facilitates* MYC-driven lymphomagenesis, rather than acting as a tumour suppressor, and that it does so by limiting apoptosis. Importantly, we have shown that MNT suppresses apoptosis by dampening expression of BIM, one of the most important apoptosis triggers for B and T lymphoid cells [54]. Like MYC, MNT binds to E boxes near many genes [1, 46]. Therefore, in addition to suppressing apoptosis, MNT may have other roles in facilitating MYC-driven oncogenesis.

MYC is a major driver for many (perhaps most) lymphoid and myeloid tumours and indeed a variety of solid tumours [3, 4]. However, the protracted search for a clinically effective MYC inhibitor has not yet succeeded [55, 56]. The realisation that MNT suppresses MYC-driven apoptosis opens an entirely new therapeutic approach: inhibition of MNT to amplify MYC’s capacity to drive apoptosis.

## Supplementary information

Supplementary Figures

Legends for Supplementary Figures

Supplementary Materials and Methods

Table S1

Table S2

Table S3

Table S4

Table S5

Pre-AssemblyWestern blots

aj-checklist
