# Supplementary material for: MNT suppresses T cell apoptosis via BIM and is critical for T lymphomagenesis
Source: Cell Death Differ. 2023 Feb 8;30(4):1018–32. doi: 10.1038/s41418-023-01119-y (PMC10070419; doi:10.1038/s41418-023-01119-y)
Supplement: Supplementary file 1 — Supplementary Figures [file 41418_2023_1119_MOESM1_ESM.pdf]

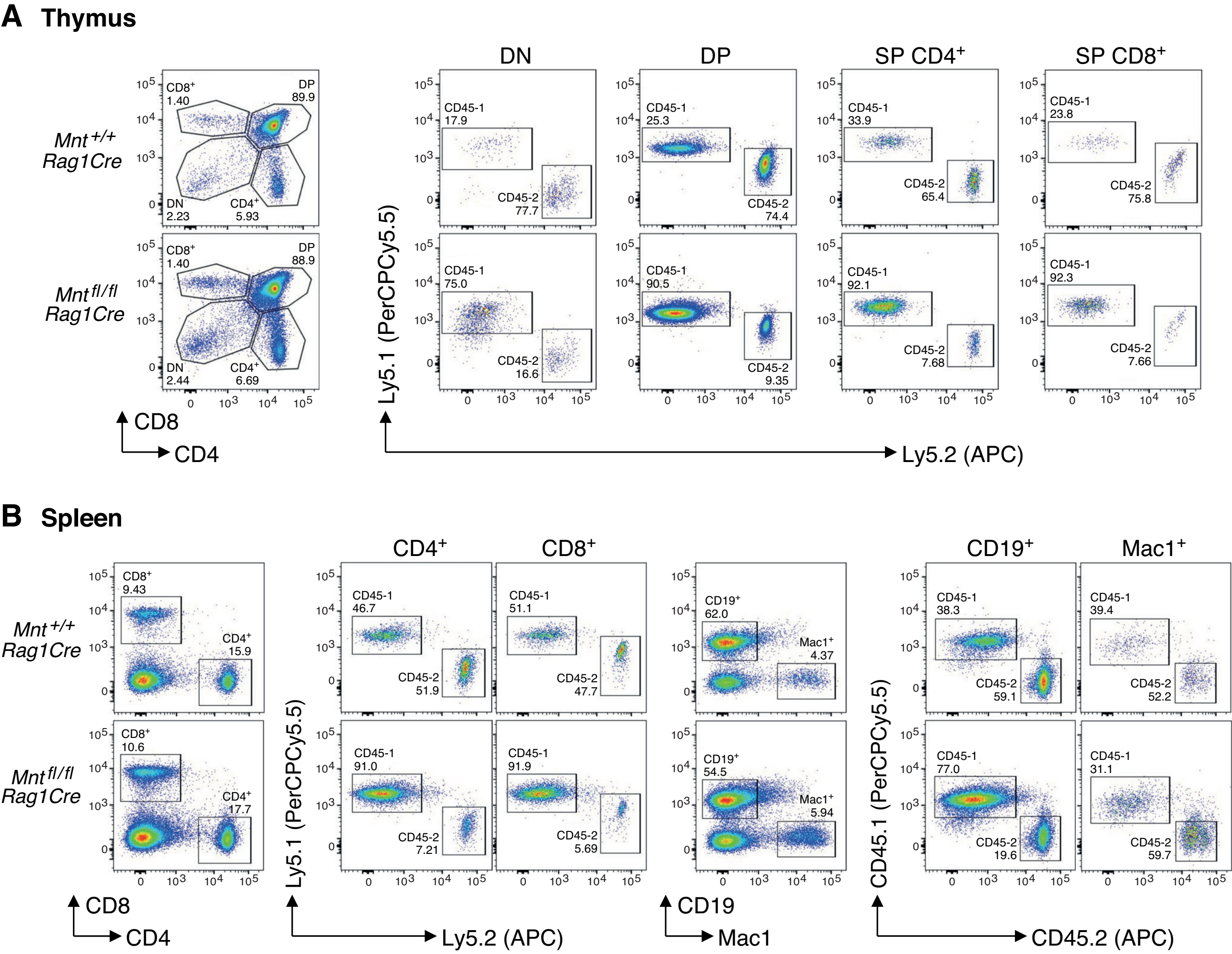

Nguyen et al Figure S1

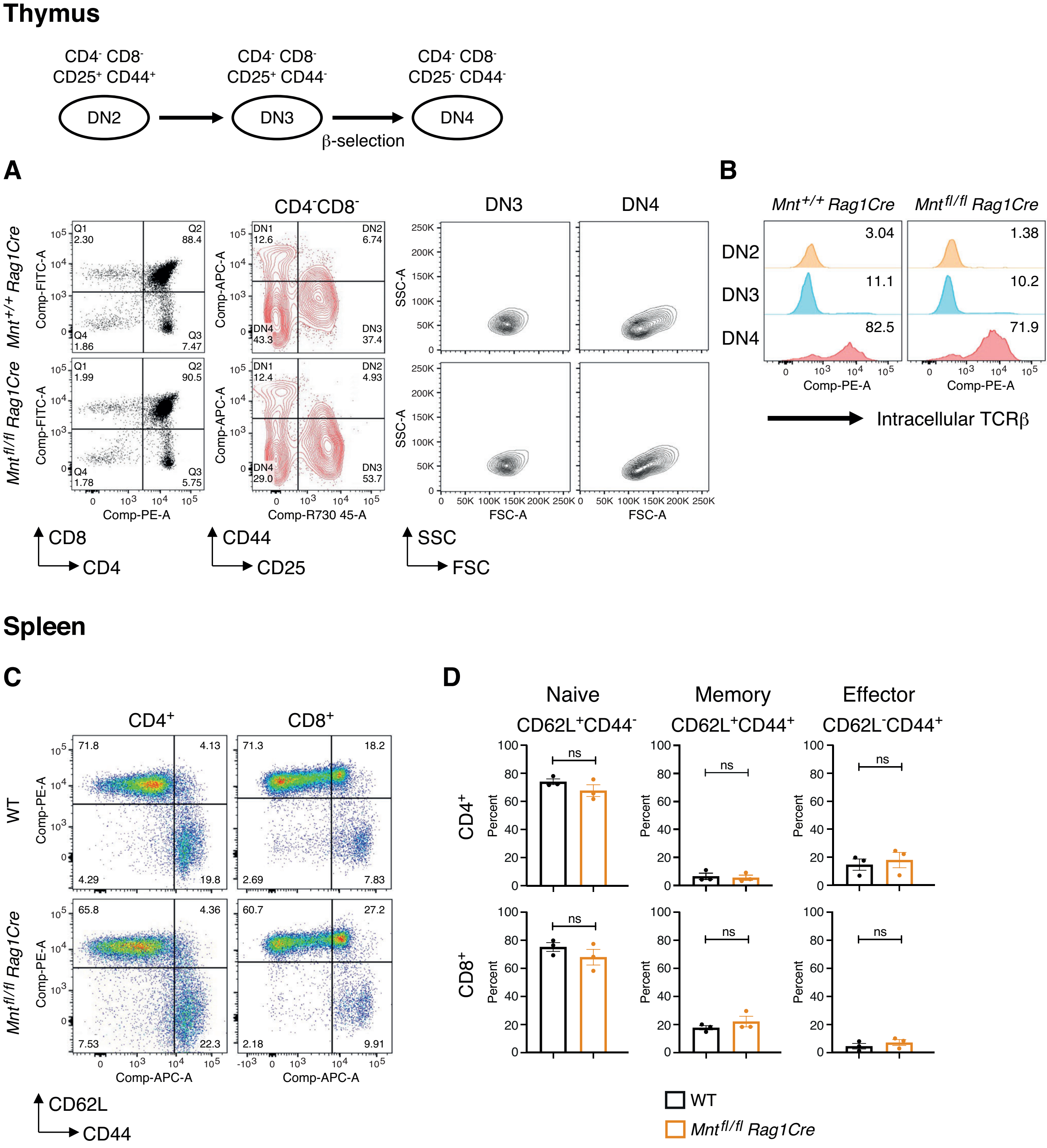

Nguyen et al Figure S2

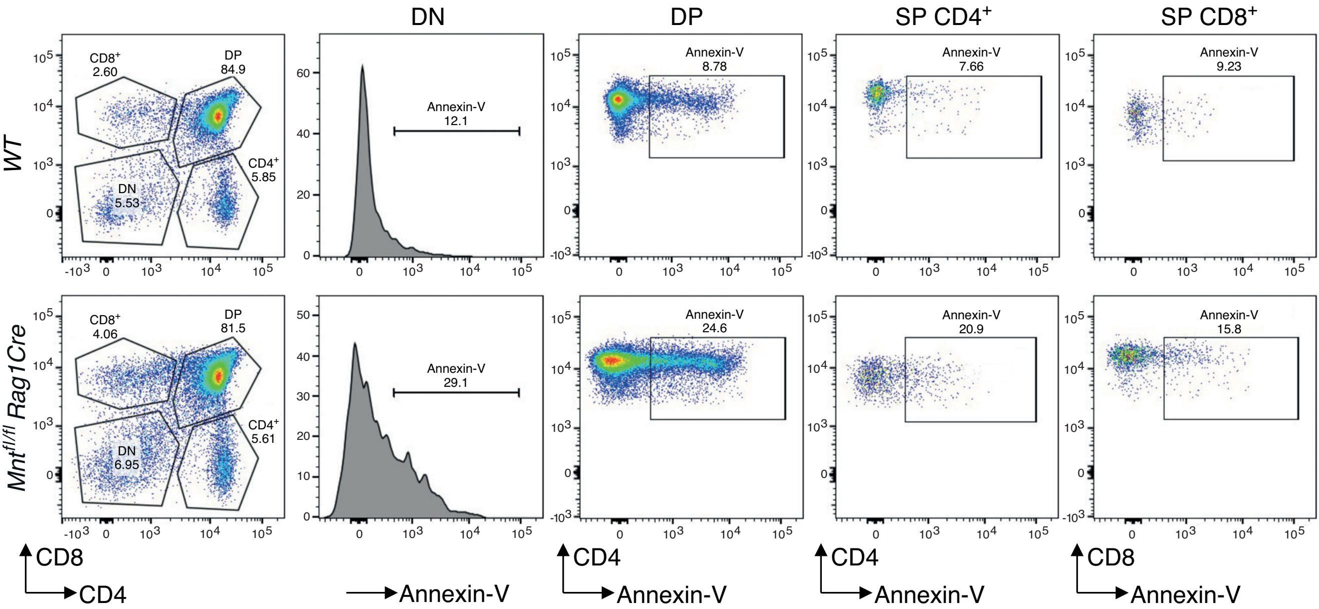

Nguyen et al Figure S3

Cultured DN3 and DN4 thymocytes

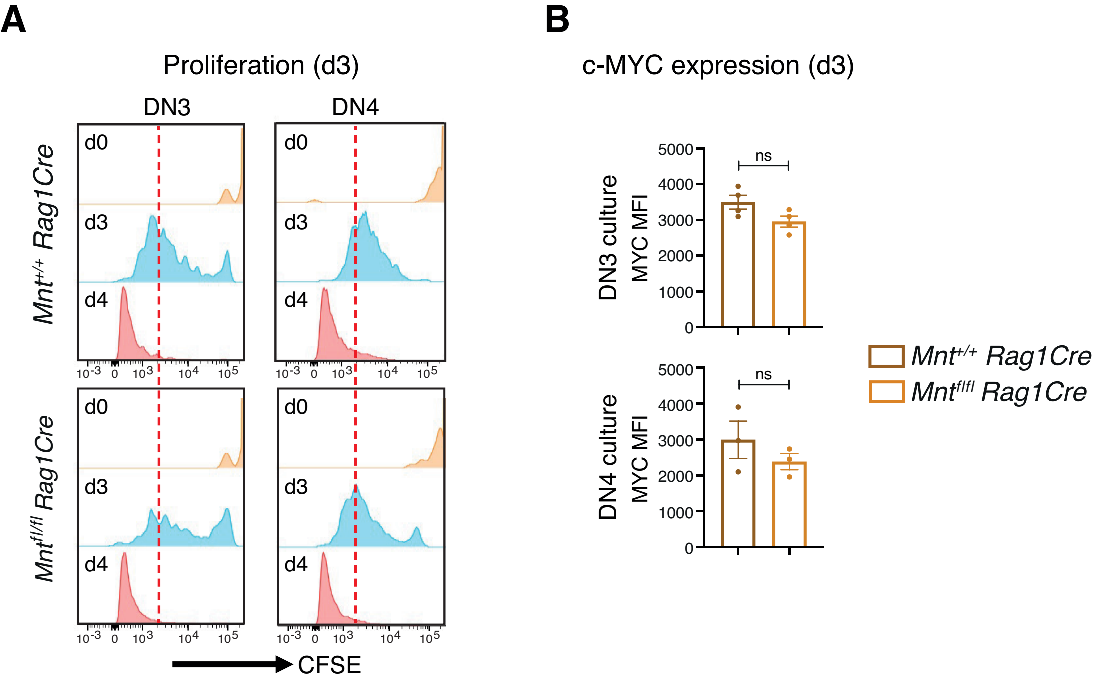

Cultured splenic T cells (PMA + ionomycin, 72 h)

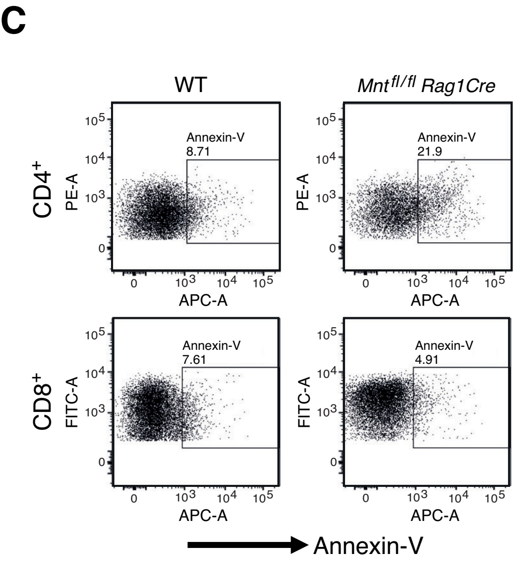

Nguyen et al Figure S4

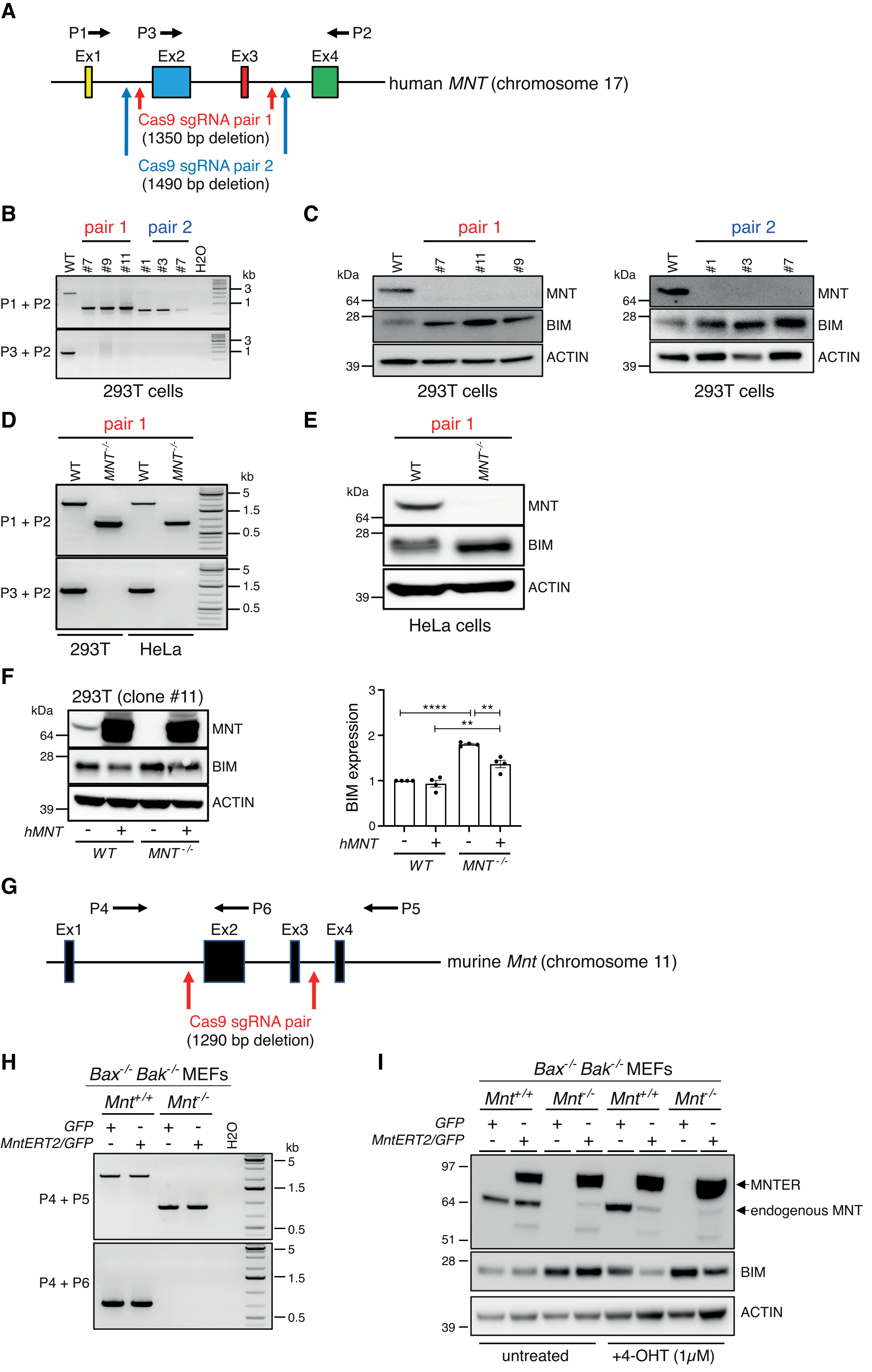

Nguyen et al Figure S5

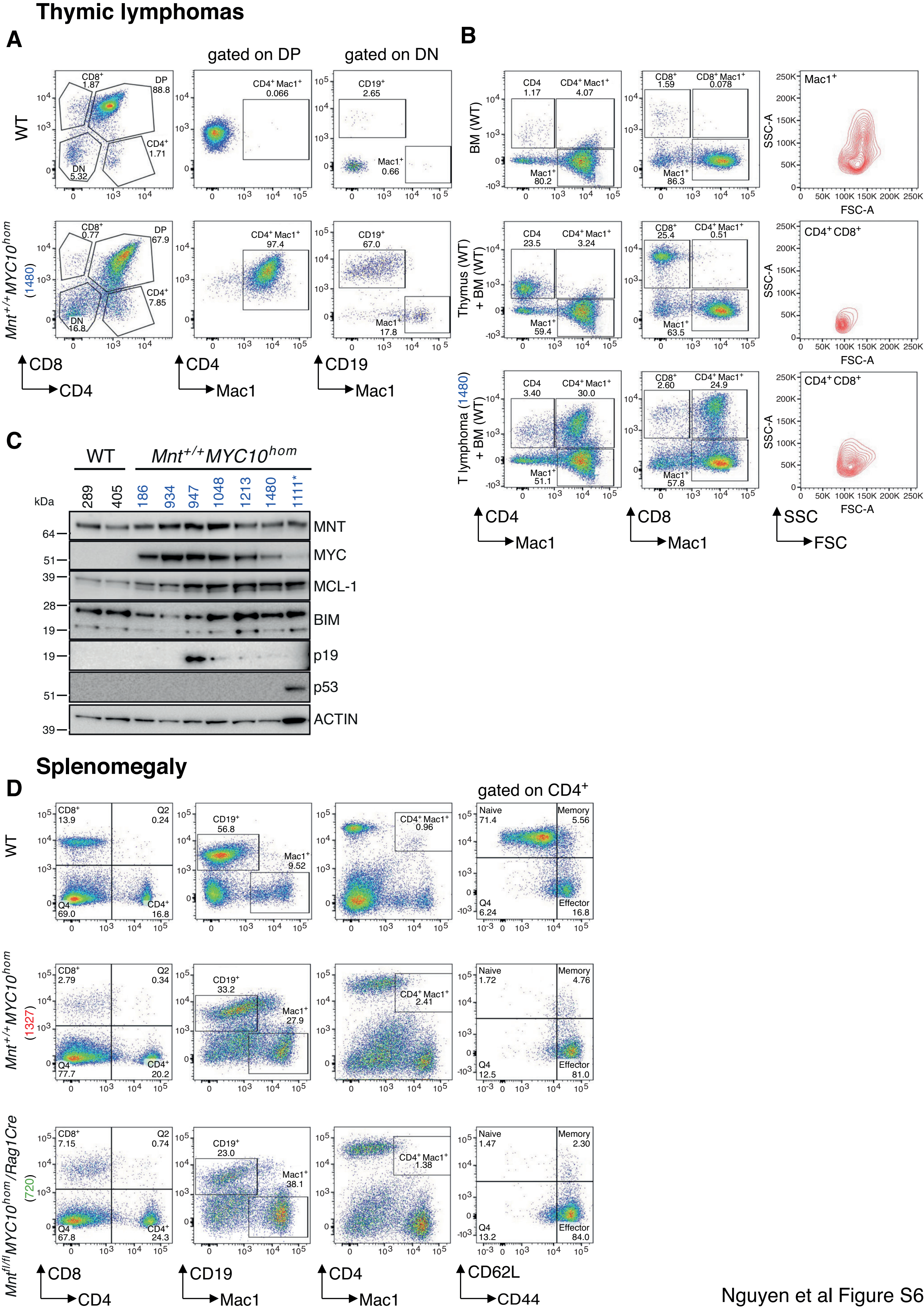

Nguyen et al Figure S6

Thymus

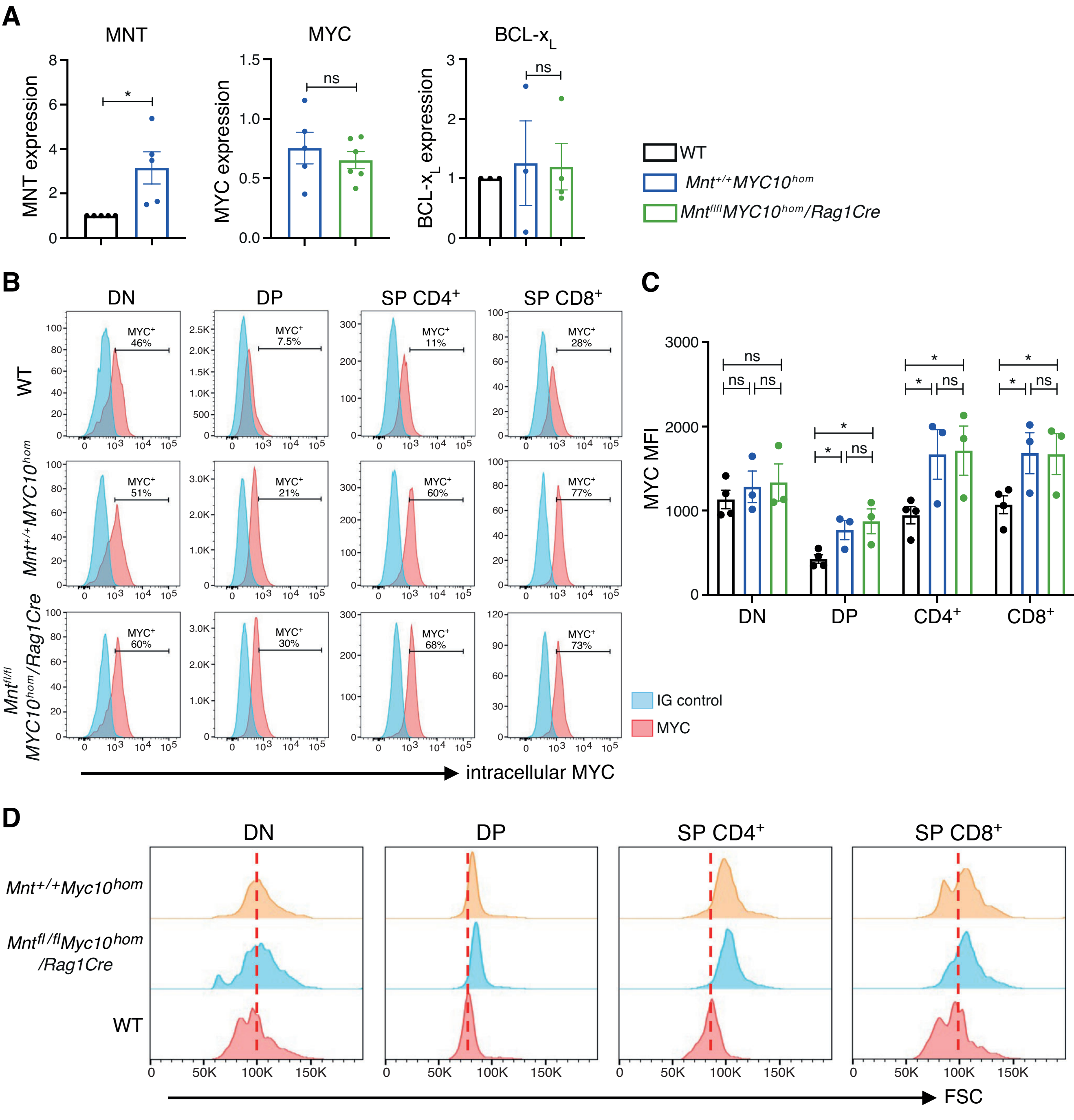

Nguyen et al Figure S7

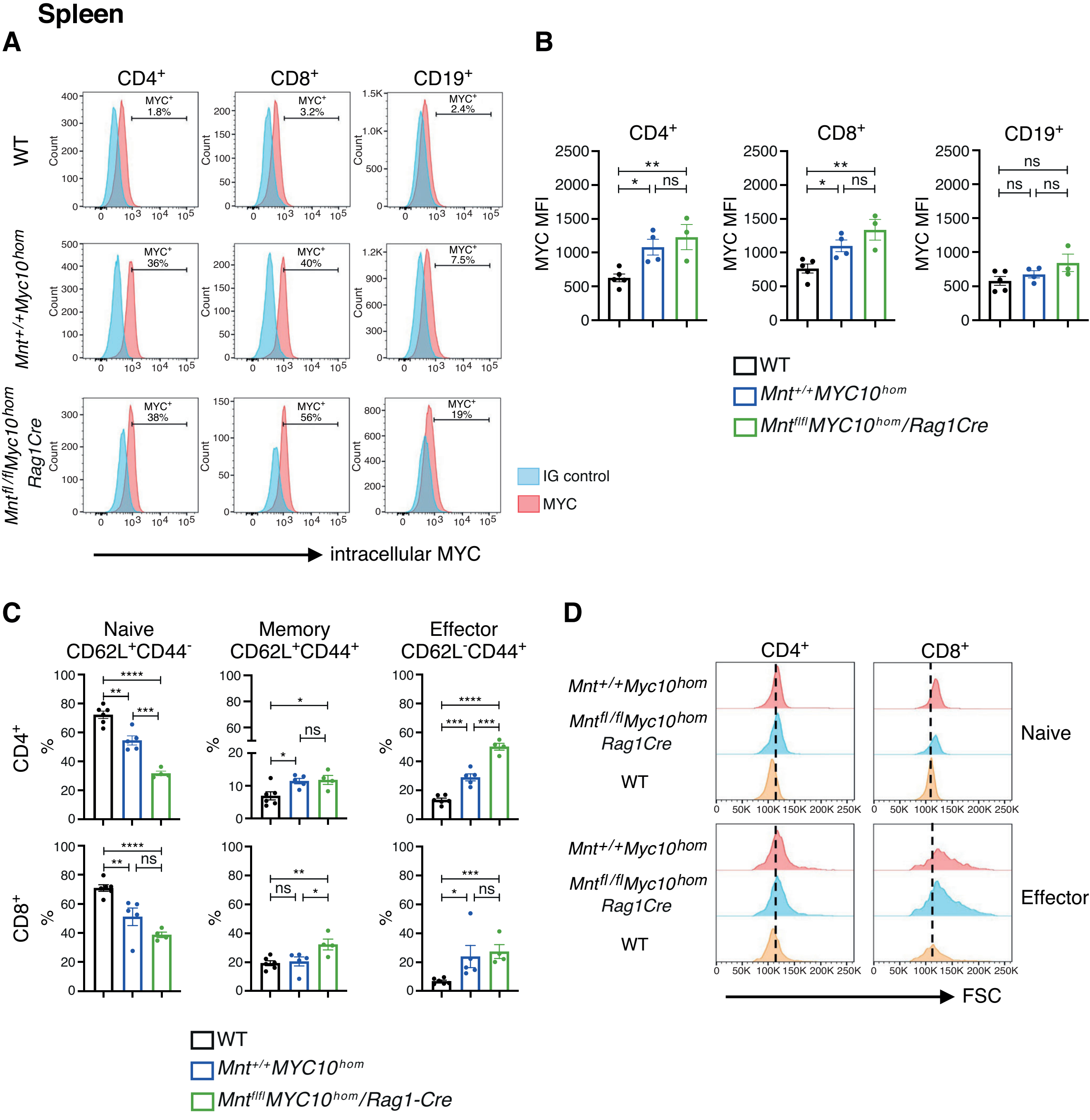

Nguyen et al Figure S8

Bone Marrow

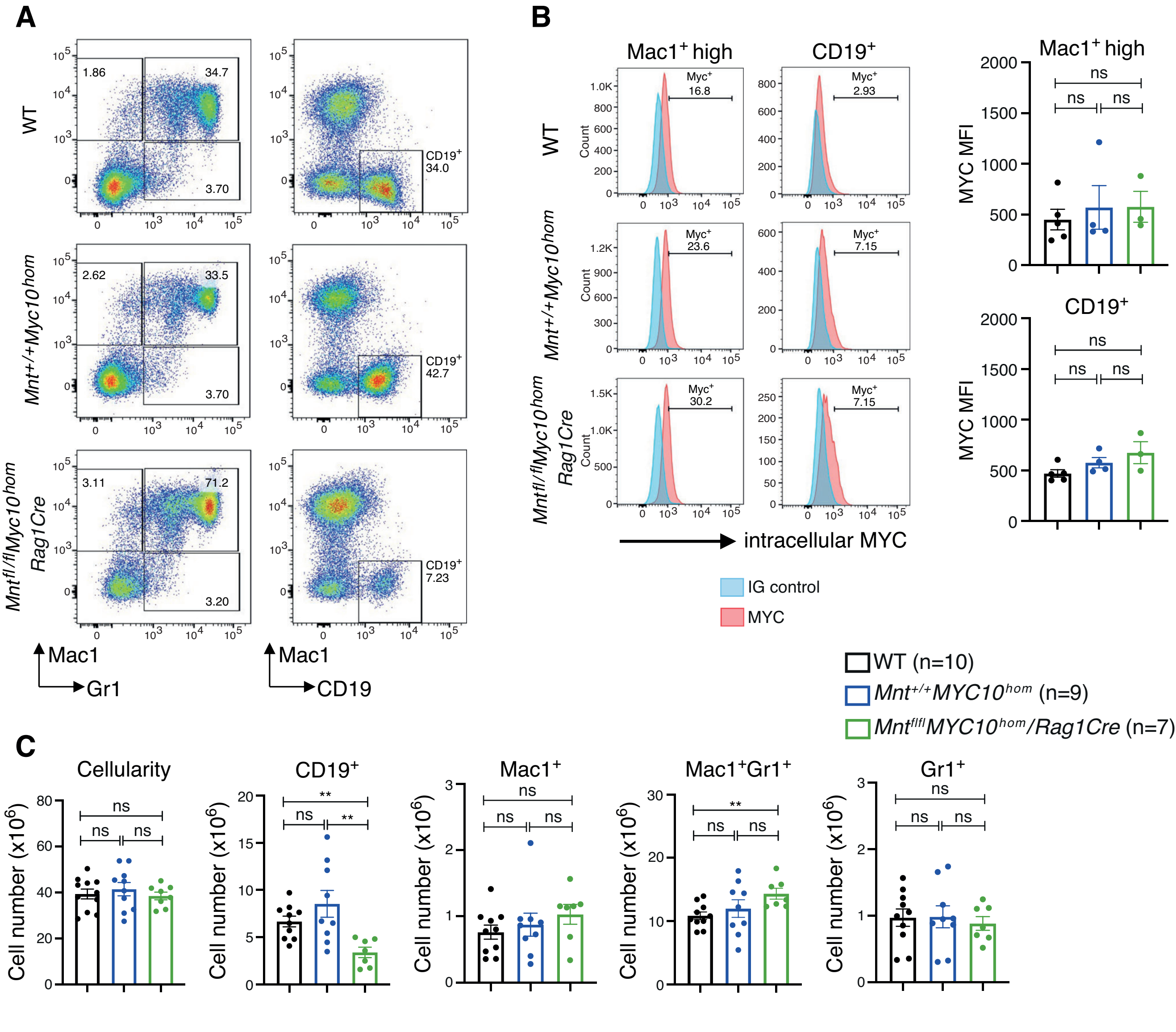

Nguyen et al Figure S9
