# Supplementary material for: MNT suppresses T cell apoptosis via BIM and is critical for T lymphomagenesis
Source: Cell Death Differ. 2023 Feb 8;30(4):1018–32. doi: 10.1038/s41418-023-01119-y (PMC10070419; doi:10.1038/s41418-023-01119-y)
Supplement: Supplementary file 2 — Legends for Supplementary Figures [file 41418_2023_1119_MOESM2_ESM.pdf]

MNT suppresses T cell apoptosis via BIM and is critical for T lymphomagenesis

Hai Vu Nguyen, Cassandra J Vandenberg, Mikara R Robati, Ashley P Ng and Suzanne Cory
The Walter and Eliza Hall Institute of Medical Research, Melbourne, VIC, Australia; and Department of Medical Biology, University of Melbourne, Melbourne, VIC, Australia.

Supplementary Figure Legends

Fig. S1 Competitive haemopoietic reconstitution between WT and *Mnt*-deleted bone marrow cells. **A** Gating strategy for determining the proportions of Ly5.1^{+} and Ly5.2^{+} thymocyte sub-populations in lethally irradiated bone marrow-reconstituted recipient mice. **B** Gating strategy to determine the proportions of the indicated Ly5.1^{+} and Ly5.2^{+} lymphoid and myeloid cell populations in the spleen. WT competitor cells are Ly5.1^{+} and test competitor cells are Ly5.2^{+}, either *Mnt*$^{+/+}$*Rag1-Cre* or *Mnt*$^{fl/f}$*Rag1-Cre*.

Fig. S2 Analysis of T cell populations in *Mnt* KO versus WT mice. **A** Gating strategy to identify DN2, DN3 and DN4 cells in the thymus. SSC= side-scattered light, FSC = forward-scattered light. **B** MNT loss does not prevent *Tcrβ* gene rearrangement. Intracellular FACS staining showing that *Mnt*$^{fl/fl}$*Rag1Cre* and *Mnt*$^{+/+}$*Rag1Cre* DN4 cells express comparable levels of TCRβ protein. **C, D** MNT loss does not alter the proportions of naïve, memory and effector CD4^{+} and CD8^{+} T cells in spleens. **C** shows typical FACS profiles after staining for CD4, CD8, CD62L and CD44; **D** shows percent of total cell numbers ± SEM for cell subsets of each of the indicated immunophenotypes. Mice were 6-7 wks old; genotypes were *Mnt*$^{fl/fl}$*Rag1Cre* (gold) and WT (black); n=3 for each genotype; ns = not significant.

Fig. S3 MNT loss enhances apoptosis of thymic T lymphoid cells. Typical annexin V-staining of the four major sub-populations of thymic T lymphoid cells from 6-7 wk old WT and *Mnt*$^{fl/fl}$*Rag1Cre* mice (see Figure 3A for aggregated results from multiple mice).

Fig. S4 Characterisation of cultured *Mnt*-deleted T lymphoid cells. **A** MNT loss does not impede proliferation of DN3 and DN4 T cells. DN3 and DN4 cells from thymi of *Mnt*$^{fl/fl}$*Rag1Cre* and *Mnt*$^{+/+}$*Rag1Cre* mice were labelled with CFSE before co-culturing with OP9-DL1 stromal cells with added IL-7. Cellular CFSE content was determined by flow cytometric analysis on days 0, 3 and 4. **B** MYC levels are not affected by MNT loss. Non-

adherent cells in d3 cultures of DN3 and DN4 cells from the thymus were permeabilised and stained with an antibody against MYC or an Ig isotype-matched control antibody, and mean intracellular fluorescence (MFI) was determined for intracellular MYC ± SEM. Genotypes analysed included *Mnt*$^{+/+}$*Rag1Cre* (brown) *and Mnt*$^{fl/fl}$*Rag1Cre* (gold). ns = not significant. **C** Exemplar of annexin-V staining of CD4^{+} and CD8^{+} splenic T cells after activation *in vitro* with PMA and ionomycin for 72 h (See Figure 4D for aggregated results from multiple mice).

Fig. S5 BIM is also a critical regulator of apoptosis in non-lymphoid human and mouse cell lines lacking MNT. **A** Human *MNT* locus (not to scale) indicating exons and locations of paired sites (red and blue arrows) used for CRISPR/Cas9-mediated gene deletion. Sites of P1, P2 and P3 oligonucleotides used for genomic PCR analysis are also indicated. The deletion of exons 2 and 3 results in splicing between exon 1 and exon 4 and production of *MNT* mRNA encoding a stop codon early in exon 3, resulting in a 28 amino acid human MNT peptide. **B** PCR analysis showing homozygous *MNT* gene deletions in the indicated (#) clones of 293T HEK cells caused by two independent pairs of Cas9/sgRNAs (see Supplementary Materials and Methods). **C** Western blots of independent clones of *MNT*$^{-/-}$ 293T cells showing absence of MNT protein and elevated BIM protein compared to control parental cells. **D, E** BIM is also elevated in *MNT*$^{-/-}$ HeLa cells. **D** Genomic PCR analysis of HeLa cells cloned after CRISPR/Cas9-mediated *MNT* gene deletion, showing identical sized PCR products as in *MNT*$^{-/-}$ 293T cells. **E** Western blot of *MNT*$^{-/-}$ HeLa cells showing absence of MNT protein and elevated BIM protein. **F** Re-expression of MNT in *MNT*$^{-/-}$ 293T clone #11 significantly reduces the level of BIM. WT and MNT KO 293T cells were transfected with 1 μg pcDNA3 empty vector (-) or pcDNA3-h*MNT* vector (+) (see Supplementary Materials and Methods). The left panel shows a typical Western blot and the right panel quantifies BIM in four independent Western blots, expressed relative to ACTIN (protein loading control) and normalised to value for parental WT cells transfected with the empty vector. **G** The mouse *Mnt* locus (not to scale) indicating exons and sites (red arrows) used for CRISPR/Cas9-mediated deletion in *Bax*$^{-/-}$*Bak*$^{-/-}$ mouse embryo fibroblasts (MEFs). The locations of P4, P5 and P6 oligonucleotides used for genomic PCR analysis are also indicated. **H** PCR analysis confirming *Mnt*$^{-/-}$ genotype of *Bax*$^{-/-}$*Bak*$^{-/-}$ MEFs used for infection with *MntERT2* and control MIG retroviruses. **I** Western blot analysis showing that BIM protein is elevated in *Mnt* $^{-/-}$ *Bax*$^{-/-}$*Bak*$^{-/-}$ MEFs compared to *Mnt* $^{+/+}$ *Bax*$^{-/-}$ *Bak*$^{-/-}$ MEFs and reduced after acute activation of MNTERT2 protein by 4-OHT. MEFs of the indicated genotypes were infected with *MntERT2* or control MIG retroviruses and infected

(GFP^{+}) cells were selected by FACS sorting prior to culture in the presence or absence of 1 μM 4-OHT.

Fig. S6 Characterisation of thymic lymphomas and splenomegaly. **A-C** Thymic lymphomas are CD4^{+}CD8^{+}Mac1^{+} (see also Supplementary Tables S1-S4). (A**)** Immunophenotyping of WT thymus and *Mnt*$^{+/+}$*MYC10*^{hom} thymic lymphoma #1480. (B**)** FACS analysis of normal BM cells (top panels); a mixture of WT BM cells plus WT thymus cells (middle panels); and WT BM cells plus #1480 lymphoma cells, revealing that Mac1^{+} level on thymic lymphoma cells is comparable to that on Mac1^{+} myeloid cells in normal BM. The right panels show enlarged size of lymphoma cells compared to normal CD4^{+}CD8^{+} thymocytes. (C**)** Western blot of thymic lymphomas from individual *Mnt*$^{+/+}$*MYC10*^{hom} mice (blue) and DP thymocytes from individual WT mice (black). Tumours #186, #934, #947, #1048, #1213 and #1480 are all CD4^{+}CD8^{+}Mac1^{+} T cell tumours. Note the lower level of transgene expression in asterisked tumour #1111, which is a rare CD19^{+} B lymphoid tumour (see Table 1). **D** Immunophenotyping of enlarged spleens from *Mnt*$^{+/+}$ *MYC10*^{hom} mouse #1327 and *Mnt*$^{fl/fl}$*MYC10*^{hom}*/Rag1Cre* mouse #720 showing elevated proportions of Mac1^{+} myeloid cells and activated CD4+ T cells compared to WT spleen. Transplantation tests of sorted cells showed the Mac1^{+} cells to be malignant but not the CD4^{+} T cells (Supplementary Table S4).

Fig. S7. Characterisation of thymic T cells in pre-malignant *MYC10*^{hom} mice. **A** Quantitation of MYC, MNT and BCL-X$_{L}$ protein relative to ACTIN (protein loading control) in western blots of sorted DP thymocytes from mice of the indicated genotypes (see legend to Fig. 7D). **B, C** MNT loss does not alter MYC levels in thymocytes of premalignant *MYC10*^{hom} mice. (B) Exemplars of intracellular MYC staining of DN, DP, SP CD4^{+} and SP CD8^{+} thymocytes in WT, *Mnt*$^{+/+}$*MYC10*^{hom} and *Mnt*$^{fl/fl}$*MYC10*^{hom}*/Rag1Cre* mice. Staining with an antibody against MYC (pink) *vs* staining with an Ig isotype matched control antibody (blue). (C**)** Mean Fluorescence Intensity (MFI) $\pm$ SEM for intracellular MYC for each major thymocyte sub-population from 3-4 independent mice (dots) for each genotype: WT (black), *Mnt*$^{+/+}$*MYC10*^{hom} (blue) and *Mnt*$^{fl/fl}$*MYC10*^{hom}*/Rag1Cre* (green). **D** MYC overexpression results in increased size of all four major thymocyte sub-populations in *MYC10*^{hom} mice and this is not affected by MNT loss. Representative exemplars of forward scatter determined by flow cytometric analysis of DN, DP, SP CD4^{+} and SP CD8^{+} thymocytes from 8 wk-old WT, *Mnt*$^{+/+}$*MYC10*^{hom} and *Mnt*$^{fl/fl}$ *MYC10*^{hom}*Rag1Cre* mice.

Fig. S8 Characterisation of splenic T cells in pre-malignant *MYC10*^{hom} mice. **A**, **B** MNT loss does not alter MYC levels in splenic T and B lymphoid cells of 8 wk-old pre-malignant *MYC10*^{hom} mice. **A** Exemplars of intracellular MYC staining of CD4^{+} and CD8^{+} T cells, and CD19^{+} B lymphoid cells from the spleens of WT, *Mnt*$^{+/+}$*MYC10*^{hom} and *Mnt*$^{fl/fl}$*MYC10*^{hom}*/Rag1Cre* mice. Note that expression of transgenic MYC protein is higher in T than B lymphoid cells. **B** Mean Fluorescence Intensity (MFI) ± SEM for intracellular MYC for CD4^{+}, CD8^{+} and CD19^{+} cells from 4-5 independent mice (dots) for each genotype: WT (black), *Mnt*$^{+/+}$*MYC10*^{hom} (blue) and *Mnt*$^{fl/fl}$*MYC10*^{hom}*/Rag1Cre* (green). **C** Effector CD4^{+} and CD8^{+} T cells are increased in the spleens of *MYC10*^{hom} transgenic mice compared to WT mice and MNT loss results in a further increase of effector CD4^{+} T cells. Flow cytometric quantification (see Fig. S1) of the proportions of naïve, memory and effector cells in the CD4^{+} (top) and CD8^{+} (bottom) populations. **D** Representative examples of relative size of naïve and effector CD4^{+} and CD8^{+} splenic T cells of the indicated genotypes, as determined by forward light scatter (FSC). ** $P \leq 0.01$; ***$P \leq 0.001$, **** $P \leq 0.0001$, ns=not significant.

Fig. S9 Characterisation of bone marrow cells in pre-malignant *MYC10*^{hom} mice. **A** Strategy for sorting bone marrow cells. **B** Lymphoid-specific MNT loss does not affect MYC expression in bone marrow B lymphoid cells. Exemplars of representative intracellular MYC staining for myeloid (left panels) and B lymphoid (middle panels). Right panels show MYC mean fluorescence intensity (MFI) for myeloid cells (upper) and B lymphoid (lower) cells from individual WT (black), *Mnt*$^{+/+}$*MYC10*^{hom} (blue) and *Mnt*$^{fl/fl}$*MYC10*^{hom}*/Rag1Cre* (green) mice. **C** Flow cytometric quantification of total cellularity of the indicated sub-populations in bone marrow of 8 wk-old (pre-malignant) WT (black), *Mnt*$^{+/+}$*MYC10*^{hom} (blue) and *Mnt*$^{fl/fl}$*MYC10*^{hom}*/Rag1Cre* (green) mice. Dots indicate individual mice, and columns show mean ± SEM; ** $P \leq 0.01$; ns=not significant.
