# Supplementary material for: MNT suppresses T cell apoptosis via BIM and is critical for T lymphomagenesis
Source: Cell Death Differ. 2023 Feb 8;30(4):1018–32. doi: 10.1038/s41418-023-01119-y (PMC10070419; doi:10.1038/s41418-023-01119-y)
Supplement: Supplementary file 3 — Supplementary Materials and Methods [file 41418_2023_1119_MOESM3_ESM.pdf]

MNT suppresses T cell apoptosis via BIM and is critical for T lymphomagenesis

Hai Vu Nguyen, Cassandra J Vandenberg, Mikara R Robati, Ashley P Ng and Suzanne Cory

The Walter and Eliza Hall Institute of Medical Research, Melbourne, VIC, Australia; and Department of Medical Biology, University of Melbourne, Melbourne, VIC, Australia.

Supplementary Materials and Methods

Mouse breeding

All mice used in this study were on a C57BL/6 background. They were bred, housed and monitored in the Bioservices Facility at the Walter and Eliza Hall Institute (WEHI) under the supervision of trained veterinarians and in accordance with the WEHI animal ethics committee regulations and the Australian Code for the Care and Use of Animals for Scientific purposes. Mice used were VavP-$MYC10^{hom}$, which express human *MYC* cDNA under the control of the *Vav* gene promoter;[1] $Bim^{-/-}$ del339/1; [2] *Rag1Cre*;[3] $Mnt^{fl/+}$ (kind gift of PJ Hurlin), [4] originally on a 129/S6 background and backcrossed for several generations to C57BL/6 mice before sent in 2005 to WEHI, where they were further extensively backcrossed and then maintained on a C57BL/6 background.

Three breeding colonies were established to generate *Mnt*-deficient mice:

(1) ERM colony: $Mnt^{fl/fl}$ mice bred with *Rag1Cre* mice to generate $Mnt^{fl/fl}$ *Rag1Cre* mice.

(2) BERM colony: $Mnt^{fl/fl}$ *Rag1Cre* mice bred with $Bim^{-/-}$ mice to generate $Bim^{+/-}$ $Mnt^{fl/fl}$ *Rag1Cre* mice.

(3) 10RM colony: Mice homozygous for the *VavP-MYC10* locus ($MYC10^{hom}$ mice) were generated by crossing *MYC10* mice. Transgenic offspring were identified by PCR analysis with specific primers detecting the *MYC10* transgene. Retro-orbital bleeds were also collected at 6 weeks and blood platelets measured using the Advia cell counter (Bayer) since $MYC10^{hom}$ mice have diagnostically useful low blood platelets (less than 700).[5] $MYC10^{hom}$ males were bred with $Mnt^{fl/fl}$ *MYC10* females to generate $Mnt^{fl/fl}$ $MYC10^{hom}$ mice. $Mnt^{fl/fl}$ $MYC10^{hom}$ males were crossed with $Mnt^{fl/fl}$ *MYC10/Rag1Cre* females to generate *Mnt*-deficient $MYC10^{hom}$ mice. $MYC10^{hom}$*/Rag1Cre* control mice were generated by breeding $Mnt^{+/+}$ $MYC10^{hom}$ males with *MYC10/Rag1Cre* females.

In our original studies of independent lines of *VavP-MYC* mice, we found that the dominant haemopoietic tumour type varied according to MYC level, with T lymphomas

prevalent only at the highest transgenic MYC levels but malignant myeloid disease apparent by histology in all.[1, 5] We note that, in our current *MYC10*^{hom} colony, thymic T lymphomas develop more slowly and with reduced penetrance than reported previously. [5] We infer that expression of the *MYC10*^{hom} transgenic locus has decreased over time, perhaps due to methylation, a well-described phenomenon for transgenes[6].

Mouse genotype analysis

Mouse genotypes were determined by PCR analysis of tail DNA isolated by standard procedures. 1 µL of tail DNA extract was added to 19 µL GoTaq Green Master Mix (Promega M7123) containing the primers (final concentration 0.5 pmol/µL) PCR program was 94°C for 3 min followed by 30 cycles (94°C for 30 sec, 58°C for 30 sec, 72°C for 40 sec) and finally 72°C for 5 min. PCR products were separated by gel electrophoresis on a 2% DNA grade agarose gel (Bioline BIO-41025) in TAE buffer (40 mM Tris Acetate, 1 mM EDTA pH 8.0) containing ethidium bromide (0.2 µg/mL final concentration from Sigma) and imaged using a Gel DOC™XR+Gel Documentation system (Bio-Rad). PCR oligonucleotide primers are described below:

Myc10: 322 bp
Myc10-F: 5'CCAAGCAGAGGAGCAAAAGCTCATT 3'
Myc10-R: 5' TCGGCTCGCGAGGTTTTAC 3'

FloxMnt*:* wt 178 bp, ko 579 bp [7]
Mnt CKO-2: 5'-GTCTCAAGTCGTGGGCATTG-3'
Hygro R-S: 5'-GATGTAGGAGGGCGTGGATA-3'
Hygro R-R: 5'-GATGTTGGCGACCTCGTATT-3'
Mnt Seq1: 5'-CAGATTCAGTGTCCCCTGCT-3'

DelMnt*:* ko 386 bp wt 147 bp [7]
Mnt ko3: 5'-CAGGTCCTCCAAAAGAGCAG-3'
Mnt ko4: 5'-GGAGCAATGTGGAGAGAAGC-3'
Mnt wt-sense: 5'-CAGTCCCTGAAGAGGAAGGA-3'
Mnt wt-rev2: 5'-CCGGAGCACACGATCTATCT-3'

Rag1Cre: WT 400 bp Rag1Cre 550 bp [3]

Rag1Cre1: 5'-AGGAAAGGATTGGCCATTTT-3'
Rag1Cre2: 5'-CCCCTCTGAGGAATCCTTCT-3'
Rag1Cre3: 5'-CATGTTTAGCTGGCCCAAAT-3'

Bim: WT 500 bp, ko 621 bp [2]

Bim PB19: 5'-CATTGCACTGAGATAGTGGTTGA-3'
Bim PB240: 5'-CCCGTTGCACCACAGATGAA-3'
Bim PB 337: 5'-AAGAATCTAGGTTGACTCTAG-3'

Tumorigenesis

Mice in tumour development cohorts were monitored regularly and euthanised when showing signs of illness (e.g. hunched stance, ruffled coat, increased respiration, loss of mobility and/or overly enlarged lymphoid organs). Sick mice were humanely euthanised, autopsied and tissues collected for histology, immunophenotyping, PCR and western blot analysis. Peripheral blood was analysed using an ADVIA haematology analyser (Bayer) or flow cytometer (LSR1, BD Biosciences) following treatment with a buffer containing 0.168 M ammonium chloride to deplete red blood cells. Thymus, spleen, lymph nodes (axillary, brachial, inguinal) and tumour-affected organs of lymphomatous animals or the corresponding tissues from healthy mice were dissected and weighed. Samples were fixed in 10% formalin for histological analysis. Tissue sections were stained with haematoxylin and eosin and analysed by experienced haematologist APN.

Analyses of pre-leukaemic mice

8 wk-old mice were humanely euthanised. Cells were isolated from bone marrow, thymus and spleen and analysed by immunophenotyping, PCR, western blotting and other procedures, as indicated in Figure legends. A mixture of age-matched male and female mice was used for all experiments.

PCR analysis of *Tcrβ* gene rearrangement

FACS-sorted $CD4^+CD8^+$ (DP) cells from *MYC10*^{hom} thymic T lymphomas or thymi of healthy wild-type mice were lysed in 500 μL lysis buffer (10 mM Tris-HCl pH 8.0, 10 mM EDTA pH 8.0, 0.5% SDS) containing proteinase K (1:500, Sigma Cat #P4850) and incubated at 56°C

overnight. Genomic DNA was precipitated by adding 0.6 mL isopropanol. The DNA pellet was washed with 70% EtOH (x2) before resuspending in TE buffer (10 mM Tris-HCl, 1 mM EDTA pH 8.0). Then, 50 ng genomic DNA was added to 50 µL GoTaq Green Master Mix (Promega M7123) containing the specific primers detecting *Tcrβ1* or *Tcrβ2* gene rearrangements (see below). Then 20 µL of PCR products were separated by gel electrophoresis on a 1.5% DNA grade agarose (Bioline BIO-41025).

(i) *Tcrβ1* gene rearrangement was determined by nested PCR. Extracted DNA was amplified by 10-cycle touchdown PCR (10 sec at 94°C, 30 sec at 68-63°C, 2 min at 72°C) with primers *Dβ1.1*-ext (5'-GAGGAGCAGCTTATCTGGTG-3') and *Jβ1.7*-ext: (5'- AAGGGACGACTCTGTCTTAC-3').

A 1 µL aliquot from the first amplification was subjected to the second PCR for 30 cycles (10 sec at 94°C, 30 sec at 60°C, 2 min at 72°C) with nested primers *Dβ1.1*-int (5'-GGTAGACCTATGGGAGGGC-3') and *Jβ1.7*-int (5'- ACCATGGTCATCCAACACAG-3').

(ii) For analysis of *Tcrβ2* gene rearrangement, two independent PCR reactions were performed using the following primers: *Vβ5-5'* consensus (5'-CCCAGCAGATTCTCAGTCCAACAG-3') or *Vβ8-5'* consensus (5'-GCATGGGCTGAGGCTGATCCATTA-3'), and *Jβ2.7-3'* (5'-TGAGAGCTGTCTCCTACTATGGATT-3'). PCR was performed with a touchdown PCR strategy: 5 min at 94°C, followed by 30 cycles of 30 sec at 94°C, 30 sec at annealing temperature and 1 min at 72°C, and a 10 min extension at 72°C. The annealing temperature was held at 68°C, 65°C and 62°C for 5 cycles each and at 58°C for 15 cycles.

Immunophenotyping

Single cell suspensions prepared from haematopoietic tissues (healthy or invaded by lymphoma cells) were depleted of red blood cells and viable cells counted using a haemocytometer using trypan blue exclusion to identify live cells. To determine cell phenotype, 2x10^6 cells from thymus or spleen or bone marrow were resuspended in 30 µL PBS, to which was added 30 µL of the antibody combination, prepared in FcγR blocking agent (2.4G2 supernatant, WEHI). After 30 min on ice, the cells were washed with 2 mL PBS, then resuspended in PBS containing propidium iodide (PI) to a final concentration of 2 µg/mL. Dead cells (PI$^+$) were excluded from analysis. All analyses were performed on a FACS Fortessa (BD) or LSR-II Cell Analyser (BD) and data were collected from at least 10^5 viable cells. Statistical

significance was determined using Student's *t*-test (unpaired two-tailed, assuming equal variance). See Supplemental Table 5 for antibodies used.

Intracellular staining for flow cytometry

Lymphoid cells (3x10^6) were first stained for cell surface-expressed lineage markers, then fixed and permeabilised in 1 mL PFA buffer (0.5% paraformaldehyde (PFA), 0.2% Tween-20, 0.1% BSA in Phosphate-Buffered Saline (PBS)) overnight at 4°C. Cells were pelleted, washed with 2 mL PBA buffer (PBS containing 0.1% BSA) and resuspended in 50 µL PBA containing antibody (see below) and left on ice for 60 min. The cells were then washed and resuspended in 50 µL PBA containing goat anti-rabbit IgG antibody conjugated to Alexa Fluor 647 (Cat #A21244, Life Technologies; dilution 1:1000) for 30 min on ice. After pelleting at 1500 rpm and washing with 2 mL PBA, the cells were analysed using a Fortessa flow cytometer (BD Biosciences). Specific BIM signal was determined relative to staining of cells from *Bim*$^{-/-}$ mice. Specific c-MYC signal was determined relative to staining with an Ig isotype-matched control antibody. BIM antibody (dilution 1:250, clone C34C5, Cell Signalling Cat#2933). c-MYC antibody (dilution 1:1500, clone D84C12, Cell Signalling Cat#09/2018). Monoclonal rabbit (DA1E) IgG isotype control antibody (dilution 1:1500, Cell Signalling Cat#05/2018). To determine intracellular TCRβ protein, total thymocytes were first stained with antibodies against CD4, CD8, CD44 and CD25 and (see Supplemental Figure 1) and then fixed and permeabilised as described above. Cells were then resuspended in 50 µL PBA containing anti-TCRβ antibody conjugated to Alexa Fluor 647 (WEHI mAb Lab, clone H57-597, dilution 1:200).

Cell viability determination

Thymus, spleen and bone marrow were harvested into PBS containing 5% FCS on ice and cell suspensions prepared using a 100 micron steel sieve. Cells (without depletion of red blood cells) were stained for surface expression of lineage markers for 15 min on ice and then washed with 1 mL Annexin-V Binding buffer (BD Cat#5166121E). Cells were stained with Annexin-V FITC (BD Cat#556419) or Annexin-V APC (BD Cat#550474) as described by the manufacturer, and analysed by flow cytometry.

OP9-DL1 stromal cell co-culture

DN3 (7x10^4) and DN4 (4x10^4) T lymphoid progenitor cells sorted from thymi of *Mnt*$^{+/+}$*Rag1Cre* and *Mnt*$^{fl/fl}$ *Rag1Cre* mice using antibodies against CD4, CD8, CD25 and CD44 (see Supplementary Figure S2A) were cultured at 37°C in 6-well plates containing a confluent monolayer of (unirradiated) OP9 stromal cells expressing Notch ligand Delta-like 1 (OP9-DL1)[8]. Co-cultures were maintained in OP9-DL1 medium in the presence of 5 ng/mL recombinant murine IL-7 (Peprotech Cat#2171710) for 3 or 4 d. OP9-DL1 medium contains MEM alpha + Glutamax (Gibco Cat#32561-037), 10mM HEPES (Gibco Cat#15630-080), 1 mM sodium pyruvate (Gibco Cat#11360-070), 1X MEM non-essential amino acid mix (Gibco Cat#11140-050), 50 μM β-mercaptoethanol (Sigma Cat#3148) and 10% FCS. Cells were collected on d3 or d4 and passed through 40 μM cell strainer to remove the (large) OP9-DL1 cells.

CFSE labelling

Sorted thymic DN3 and DN4 T lymphoid progenitor cells were stained with 2 μM Cell Trace^{TM} CFSE dye (Thermo Fisher Cat#C34570) as described by the manufacturer before culturing in OP9-DL1 medium and analysed by flow cytometry at different time points. Sorted splenic CD4$^+$ and CD8$^+$ cells were stained with 2 μM Cell Trace^{TM} CFSE before stimulation with PMA and ionomycin.

***In vitro* stimulation of T cells with PMA + ionomycin**

3x10^5 CD4$^+$ and CD8$^+$ T cells sorted from wild-type and *Mnt*$^{fl/fl}$ *Rag1Cre* spleens were cultured in 2 mL complete Opti-MEM medium containing 20 ng/mL PMA (Sigma Cat#P8139) and 1 μg/mL ionomycin (Sigma Cat#I9657) for 72 h. Complete OptiMEM medium contains Opti-MEM (Gibco Cat#31985070), 10mM HEPES (Gibco Cat#15630-080), 1mM Sodium pyruvate (Gibco Cat#11360-070), 1X MEM non-essential amino acids (Gibco Cat#11140-050), 50 μM β-mercaptoethanol, 2 mM L-Glutamine (Gibco Cat#25030081), Penicillin-Streptomycin (Gibco Cat#11360070) and 10% FCS. Cells were harvested at 72 h for FACS and western blot analysis.

Western blot analysis

Lysates were prepared from FACS-sorted cells or from cultured cells in modified RIPA buffer (150 mM NaCl, 50 mM Tris-HCl pH 8, 1% NP-40, 0.1% SDS) containing a cocktail of protease inhibitors (Roche Cat#5892970001). Lysates from 5x10^5 viable cells (20 μL) were added to 5

µL 4xNuPAGE LDS sample buffer (Invitrogen Cat#NP0007) supplemented with 10% β-mercaptoethanol (#M3148, Sigma), then denatured at 95°C for 5 min and fractionated by electrophoresis on a 4-12% Bis-Tris SDS-PAGE gel (Invitrogen Cat#NP0335) in MOPS buffer (Invitrogen Cat#NP0001). Proteins were transferred onto nitrocellulose membranes (Invitrogen Cat#IB23001) using an iBlot2 gel transfer device (Invitrogen Cat#IB21001). Membranes were blocked with 10% non-fat dry milk powder dissolved in PBS containing 0.1% Tween 20 (called PBS-T) (Sigma Cat#P1379), then incubated with the primary antibody (see below) in block solution overnight at 4°C. Following washes in PBS-T (3x15 min at room temperature), membranes were incubated with the appropriate HRP-conjugated secondary antibody at room temperature for 60 min, followed by washing (3x15 min). Protein bands were then visualised using Immobilon Forte Western HRP substrate (Millipore Cat#WBLUF0100) and imaged on the ChemiDoc™ Touch Imaging System (Bio-Rad Cat#1708370). Prior to re-probing with another antibody, membranes were stripped of both primary and secondary antibodies by incubating for 30 min at 50°C with stripping buffer (62.5 mM Tris-HCl pH 6.8, 2% SDS, 0.7% β-mercaptoethanol) in a heat-sealed or ANOVA plastic pouch followed by 3x10 min washes in PBS-T. Membranes were probed using primary antibodies against the following proteins: MNT (1:2000, Bethyl Cat#A303-626A), BIM (1:2000, clone C34C5, Cell Signalling Cat#2933), ACTIN (1:5000, clone AC-74, Sigma Cat#A2228, c-MYC (1:2000, clone D84C12, Cell Signalling Cat#5605S), MCL-1 (1:1000, clone 19C4-15, WEHI mAB lab), p53 (1:2000, clone CM5, Novocastra™ Leica Biosystem Cat#NCL-Lp53-CM5p), P19-ARF (1:1000, Rockland Cat#200-501-891) anti-cleaved Notch1 (Val1744) (1:1500, clone D3B8, Cell Signaling). Secondary antibodies used were: anti-rabbit IgG (1:5000, Southern Biotech Cat#4010-05), anti-mouse IgG (1:5000, Jackson ImmunoResearch Cat#115-035-071), anti-rat IgG (1:5000, Southern Biotech Cat#3010-05).

Quantitative RT-PCR analysis

Total RNA was extracted from at least 10^6 sorted $CD4^+CD8^+$ thymic T cells using Trizol Reagent (Invitrogen Cat#15596026) and chloroform/isopropanol protocol. cDNA was synthesized using Oligo (dT)12-18 primer (Invitrogen Cat#18418012) and M-MLV Reverse Transcriptase (Invitrogen Cat#28025013) as described by the manufacturer. Real-time PCR was performed with Taq-man Master Mix using specific probes for murine *Bim/Bcl2l11* (Mn00437796_m1) (Thermo Fisher Cat#4331182) and, as a control, *Gapdh*

(Mm99999915_g1) (Thermo Fisher Cat#4331182). PCR reactions were run on ViiA7 Real-time PCR system (Thermo Fisher).

CRISPR/Cas9-mediated gene deletion

HEK293T: human embryonic kidney cell line expressing SV40 large T antigen (293 T, American Type Culture Collection (ATCC) #CRL-3216); HeLa ATCC CCL-2: human cervical carcinoma cell line; *Bax*$^{-/-}$*Bak*$^{-/-}$ MEFs clone Z8, immortalised using SV40 T antigen, originally a gift from Dr S Korsmeyer [9], obtained from Dr DCS Huang (WEHI). All were maintained in Dulbecco's modified Eagle's medium (DMEM) containing 10% FCS.

To delete the *MNT* gene in human HEK293T and HeLa cells, two independent pairs of sgRNAs (see below) were cloned into the pSpCas9(BB)-2A-Puro vector (PX459) (Addgene Cat#62988). Briefly, 5x10^{5} cells were seeded in DMEM medium +10% FCS in a 6 well plate 24 h before transfection. Cells were transfected using the calcium phosphate protocol (see below) with 2 µg of 5'Cas9 and 3'Cas9 sg RNA vectors, either pair-1 or pair-2 (Supplemental Figure S5A), and incubated at 37°C for 24 h, after which the medium was replaced with fresh medium containing 1 µg/mL puromycin (Sigma Cat#P9620) for a further 48 h. The cells were then distributed into 96 well plates (200 µL/well) and wells containing single cell-derived clones were identified by microscopy. For PCR genome analysis, 9.8 µL of clonal culture was added to 2x lysis buffer cocktail (200 mM Tris-HCl pH8.0, 100 mM EDTA, 0.2% Triton X100, 0.2 µL protein kinase A (PKA); (Sigma Cat#P4850) and incubated at 56°C for 4 h, after which PKA was inactivated by incubation at 95°C for 20 min. PCR analysis to identify *MNT* gene deletion used 2 µL of the cell lysate and primers P1, P2 and P3 (see below and Supplemental Figure S5A). PCR program was 94°C for 3 min followed by 35 cycles (94°C for 30 sec, 60°C for 30 sec, 72°C for 2 min) and finally 72°C for 5 min. PCR products were then separated by gel electrophoresis on 1% DNA grade agarose. The *MNT* germline band amplified by P1 and P2 primers is 2165 bp; the *MNT* germline band amplified by P3 and P2 primers is 1115 bp. *Mnt* gene deletion mediated by Pair 1 sgRNAs is 1350 bp and 1490 bp for Pair 2 sgRNAs.

To delete the *Mnt* gene in immortalised *Bax*$^{-/-}$/*Bak*$^{-/-}$ MEFs (clone Z8), sgRNAs (mCas9-Ex2 and mCas9-Ex3, see Figure S5G) were separately cloned into the PX459 Cas9 vector (Addgene Cat#62988). Briefly, 2x10^{5} cells were seeded in DMEM + 10% FCS in 6 well plates 24 h before transfection. Cells were transfected with 2 µg of 5'Cas9 and 3'Cas9 vectors and incubated at 37°C for 24 h. Transfected cells were selected with 2 µg/mL puromycin for

72 h. Single cell-derived clones were obtained by limited dilution in 96 well plates and screened by PCR (see above). The *Mnt* germline band amplified by P4 and P6 primers is 2151 bp.

pair-1 5'Cas9 5'-CCGGCACTTTCCCCCGTGCC-3'

pair-1 3'Cas9 5'-CGTGCGTGCGCGCGACCC-3'

pair-2 5'Cas9 5'-CCTACTATTCCCTGGTGGCG-3'

pair-2 3'Cas9 5'-AGCGCCGGTCAGCGGTGCCT-3'

P1 5'-AGTCAAGAGTGCTGGGCTGT-3'

P2 5'-GCCCCATACCTGGATGTACC-3'

P3 5'-CCGGACTCAGCATTAAGGAG-3'

mCas9-Ex2 5'- CAGGCTAGGCAGAACGTCGT-3'

mCas9-Ex3 5'- GCCAACGCCTGGGTATATAC-3'

P4 5'- ATCTGTCCCTGTGCACACTTT -3'

P5 5'- TGACAGTCAGTGGAGCAGG -3'

P6 5'- GATAGTAATCCTAAGCACTGCCA -3'

Re-expression of MNT in *MNT KO* cells

Clones of $MNT^{+/+}$ and *MNT KO* HEK293T cells were transfected with 1ug pcDNA3 empty vector or pcDNA3-h*MNT* vector (GenScript Cat#SC1200) using the calcium phosphate protocol, using 2M $CaCl_2$ stock (Sigma Cat#C5080) and 2xHBS solution (0.05 M HEPES buffer, Gibco Cat#15630-080; 1.5 mM Na_2HP04, Sigma Cat#S7907; 0.28 M NaCl, Sigma Cat#S9888). Cells were collected 48 h after transfection and lysed in modified RIPA buffer buffer (see western blot above).

To prepare retrovirus-containing supernatants for infection of $Bax^{-/-}/Bak^{-/-}$ MEFs, HEK293T cells ($0.5x10^6$) were seeded 3 ml of DMEM plus 10% FCS in 6 well plates 24 h before transfection with *pMIG* (4 µg) (Addgene #9044) or p*MIG* m*MntERT2* (4 µg) DNA (*mMntERT2* synthesised by Thermo Fisher, Cat #817003DE and cloned into *pMIG* vector), together with retrovirus packaging plasmids (2 µg of *GAG/POL* Addgene Cat# 14887 and 1 µg of *ENV* Addgene Cat# 8454 vectors) using the calcium phosphate protocol. After cells had been incubated at 37°C for 24 h, the medium was replaced and supernatants were collected 48 h later. Clones of $Mnt^{+/+}$ and $Mnt^{-/-}$ $Bax^{-/-}/Bak^{-/-}$ MEFs ($0.5x10^6$ in 1 mL)) were infected by incubation overnight with retrovirus-containing supernatants (3 mL) in the presence of polybrene (4 µg/mL). The medium was replaced 24 hr after infection and, after a further 24 hr, GFP^+ cells were purified using a FACS Aria™ III cell sorter (BD) and incubated with 1 mM

4OHT (Sigma Cat#H7904) for 48 h, which allows the MNTERT2 protein to move from its sequestration by HSP90 in the cytoplasm into the nucleus. Cells were collected at 48 h and lysed in modified RIPA buffer buffer (see western blot above).

Antibodies used for flow cytometry and immunoblotting

**Antibody specificity**	**Fluorochrome**	**Source**	**Catalogue**	**Dilution**
CD19	perCP-Cy5.5	BioLegend (clone 1D3)	152406	1:300
CD19	PE	WEHI mAb Lab (clone 1D3)		1:400
CD19	FITC	WEHI mAb Lab (clone 1D3)		1:400
CD4	PE	WEHI mAb Lab (clone GK1-5)		1:400
CD4	FITC	WEHI mAb Lab (clone GK1-5)		1:400
CD4	A700	WEHI mAb Lab (clone GK1-5)		1:400
CD4	APC	WEHI mAb Lab (clone GK1-5)		1:400
CD8	PE	WEHI mAb Lab (clone 53-6-7)		1:400
CD8	FITC	WEHI mAb Lab (clone 53-6-7)		1:400
CD44	APC	WEHI mAb Lab (clone IM7.81)		1:400
CD25	A700	WEHI mAb Lab (clone PC61/F7)		1:200
CD25	APC	WEHI mAb Lab (clone PC61/F7)		1:200
CD62L	PE	WEHI mAb Lab (clone MEL14)		1:400
TCRβ	Alexa Fluor 647	WEHI mAb Lab (clone H57-597)		1:200
CD45.1	PerCP-Cy5.5	BioLegend (clone A20)	110728	1:200
CD45.2	APC	WEHI mAb Lab (clone IM7.81)		1:200
Mac1	APC	WEHI mAb Lab (clone M1/70)		1:400
Mac1	PE	WEHI mAb Lab (clone M1/70)		1:400
Gr1	PE	WEHI mAb Lab (clone RB6-8C5)		1:400
MCL-1		WEHI mAb Lab (rat monoclonal; clone 19C4-15)		1:2000
p53		Novocastra (rabbit monoclonal; clone CM5)	CM5	1:2000
c-MYC		Cell Signalling (rabbit monoclonal; clone D84C12)	560-S	1:2000
BIM		Cell Signalling (rabbit monoclonal; clone C34C5	2933S	1:2000
MNT		Bethyl (rabbit polyclonal)	A303-626A	1:2000
p19ARF		Rockland (rat monoclonal; clone 5.C3.1)	200-501-891	1:1000
Actin		Sigma (mouse monoclonal; clone AC-74)	A5316	1:2000
BCL-X_L		WEHI mAb Lab (rat monoclonal clone 9C9.A3.C4)		1:1500
BCL-2		WEHI mAb Lab (hamster 3F11)		1:2000

cleaved NOTCH1 (Val1744)		Cell Signaling (rabbit monoclonal, clone D3B8)	4147	1:1000

REFERENCES

1. Smith DP, Bath ML, Harris AW, Cory S. T-cell lymphomas mask slower developing B-lymphoid and myeloid tumors in transgenic mice with broad hematopoietic expression of MYC. *Oncogene* 2005, **24**(22)**:** 3544-3553.

2. Bouillet P, Cory S, Zhang L-C, Strasser A, Adams JM. Degenerative disorders caused by Bcl-2 deficiency are prevented by loss of its BH3-only antagonist Bim. *Dev Cell* 2001, **1**(5)**:** 645-653.

3. McCormack MP, Forster A, Drynan L, Pannell R, Rabbitts TH. The LMO2 T-cell oncogene is activated via chromosomal translocations or retroviral insertion during gene therapy but has no mandatory role in normal T-cell development. *Mol Cell Biol* 2003, **23**(24)**:** 9003-9013.

4. Toyo-oka K, Bowen TJ, Hirotsune S, Li Z, Jain S, Ota S*, et al.* Mnt-deficient mammary glands exhibit impaired involution and tumors with characteristics of myc overexpression. *Cancer Res* 2006, **66**(11)**:** 5565-5573.

5. Smith DP, Bath ML, Metcalf D, Harris AW, Cory S. MYC levels govern hematopoietic tumor type and latency in transgenic mice. *Blood* 2006, **108**(2)**:** 653-661.

6. Garrick D, Fiering S, Martin DI, Whitelaw E. Repeat-induced gene silencing in mammals. *Nat Genet* 1998, **18**(1)**:** 56-59.

7. Toyo-oka K, Hirotsune S, Gambello MJ, Zhou ZQ, Olson L, Rosenfeld MG*, et al.* Loss of the Max-interacting protein Mnt in mice results in decreased viability, defective embryonic growth and craniofacial defects: relevance to Miller-Dieker syndrome. *Hum Mol Genet* 2004, **13**(10)**:** 1057-1067.

8. Schmitt TM, Zúñiga-Pflücker JC. Induction of T cell development from hematopoietic progenitor cells by Delta-like-1 in vitro. *Immunity* 2002, **17**(6)**:** 749-756.

9. Cheng EH, Wei MC, Weiler S, Flavell RA, Mak TW, Lindsten T*, et al.* BCL-2, BCL-x_L sequester BH3 domain-only molecules preventing BAX- and BAK-mediated mitochondrial apoptosis. *Mol Cell* 2001, **8**(3)**:** 705-711.
