# Supplementary material for: MNT suppresses T cell apoptosis via BIM and is critical for T lymphomagenesis
Source: Cell Death Differ. 2023 Feb 8;30(4):1018–32. doi: 10.1038/s41418-023-01119-y (PMC10070419; doi:10.1038/s41418-023-01119-y)
Supplement: Supplementary file 4 — Table S1 [file 41418_2023_1119_MOESM4_ESM.pdf]

Table S1 Tumours arising in *Mnt*$^{+/+}$ *vavP-MYC10*^{hom} mice.

[1]Mouse	[2]Survival	Autopsy	[3]Immunophenotype	[4]Histology	[4]Cytospin	[5]Diagnosis
910 F	69 d	massive thymus (1200 mg); normal spleen and LNs.	nd	nd	nd	thymic T lymphoma (inferred)
410 F	82 d	massive spleen (810 mg) and LNs (530 mg), MLN (350 mg); enlarged thymus (170 mg).	nd	nd	nd	disseminated myeloid tumour (inferred)
972 F	114 d	massive spleen (930 mg) and LNs (290 mg), MLN (90 mg); massive liver; enlarged thymus (160 mg).	nd	nd	nd	disseminated myeloid tumour (inferred)
1113 F	115 d	massive thymus (860 mg); enlarged spleen (250 mg).	nd	nd	nd	thymic T lymphoma (inferred)
947 M	119 d	massive thymus (1200 mg); enlarged spleen (200 mg).	thymus: 7.7% DN, 78% $CD4^+CD8^+$, 9.3% $CD4^+$,0.5% $CD8^+$, 4.9% $CD19^+$, 69% $Mac1^+$ (*87% of* $CD4^+$ *are* $Mac1^+$) clonal TCR	nd	thymus: immature lymphocytes, lymphoblasts	thymic T lymphoma ($CD4^+CD8^+Mac1^{+)}$
934 F	123 d	massive thymus (1000 mg); enlarged spleen (420 mg).	thymus: 6.2% DN, 76% $CD4^+CD8^+$, 12% $CD4^+$, 0.9% $CD8^+$, 4.3% $CD19^+$, 65% $Mac1^+$ (*85% of* $CD4^+$ *are* $Mac1^+$) clonal TCR	nd	thymus: lymphoblasts	thymic T lymphoma ($CD4^+CD8^+Mac1^+$)
969 F	125 d	massive thymus (1040 mg); enlarged spleen (280 mg).	nd	nd	nd	thymic T lymphoma (inferred)
973 F	128 d	massive thymus (980 mg); enlarged spleen (350 mg)	thymus: DN 5.7%, 76% $CD4^+CD8^+$, 10% $CD4^+$, 5.7% $CD8^+$, 7.8% $CD19^+$, 77% $Mac1^+$	nd	nd	thymic T lymphoma ($CD4^+CD8^+Mac1^+$)
821 F	132 d	massive spleen (1020 mg); massive liver; enlarged thymus (220 mg).	nd	nd	nd	disseminated myeloid tumour (inferred)

859 M	133 d	enlarged spleen	fd	nd	nd	splenic myeloid tumour (inferred)
1034 M	135 d	massive spleen (1350 mg) and LNs (510 mg); MLN (150 mg); enlarged thymus (240 mg).	nd	large, pleiomorphic cells with prominent nuclear heterochromatin invading spleen, liver, LNs, sternum.	nd	disseminated myeloid tumour
884 F	137 d	enlarged spleen (730 mg); pale liver, kidneys; enlarged thymus (130 mg).	nd	nd	nd	disseminated myeloid tumour (inferred)
991 M	139 d	enlarged thymus (420 mg); spleen (280 mg).	thymus: 6.9% DN, 82% $CD4^+CD8^+$, 11% $CD4^+$, 5.5% $CD8^+$, 5.2% $CD19^+$, 62% $Mac1^+$ spleen: 12% $CD4^+$, 6.3% $CD8^+$, 64% $CD19^+$, 12% $Mac1^+$	nd	nd	thymic T lymphoma ($CD4^+CD8^+Mac1^+$)
824 F	141 d	enlarged spleen (730 mg); tumour lump on backside; thymus enlarged (130 mg).	nd	large, pleiomorphic cells with prominent nuclear heterochromatin invading spleen, lymph nodes, sternum.	nd	disseminated myeloid tumour (inferred)
1480 F	142 d	massive thymus (1020 mg); enlarged spleen (400 mg).	thymus: 17% DN, 68% $CD4^+CD8^+$, 7.9% $CD4^+$, 0.8% $CD8^+$, 9.9% $CD19^+$, 78% $Mac1^+$ (97% *$CD4^+$ are $Mac1^+$*) clonal TCR spleen: 23% $CD4^+$, 2% $CD8^+$, 47% $CD19^+$, 9% $Mac1^+$	nd	thymus: lymphocytes with occasional large vacuolated blast	thymic T lymphoma ($CD4^+CD8^+Mac1^+$)
1564 F	143 d	massive spleen (910 mg);enlarged LNs (180 mg) pale enlarged liver; thymus 80 mg	nd	nd	nd	disseminated myeloid tumour (inferred)

1213 F	144 d	massive thymus (1190 mg); enlarged spleen (280 mg).	thymus: 20% DN, 56% $CD4^+CD8^+$, 13% $CD4^+$, 1.9%$CD8^+$, 12% $CD19^+$, 45% $Mac1^+$ (*69% of CD4+ are Mac1+*) biclonal TCR	nd	thymus: mature and immature lymphocytes and lymphoblasts	thymic T lymphoma ($CD4^+CD8^+Mac1^+$)
258 M	145 d	massive thymus (1440 mg); enlarged spleen (280 mg).	nd	nd	nd	thymic T lymphoma (inferred)
971 F	152 d	massive spleen (1540 mg) and enlarged LNs (370 mg); thymus 70 mg.	nd	nd	nd	disseminated myeloid tumour (inferred)
839 F	158 d	enlarged spleen (680 mg) and enlarged LNs; enlarged thymus (190 mg)	fd	nd	nd	disseminated myeloid tumour (inferred)
860 M	158 d	enlarged spleen (450 mg); huge lumbar LNs; ascites; enlarged liver. enlarged thymus (140 mg);	spleen: 11% $CD4^+$,2.7% $CD8^+$, 50% $CD19^+$, 35% $Mac1^+$	nd	nd	disseminated myeloid tumour
1048 M	163 d	massive thymus (820 mg); enlarged spleen (280 mg);	thymus: 19% DN, 68% $CD4^+CD8^+$, 6.9% $CD4^+$, 1.9% $CD8^+$, 16% $CD19^+$, 55% $Mac1^+$ (90% of $CD4^+$ are $Mac1^+$) biclonal TCR	nd	thymus: mature and immature lymphocytes and lymphoblasts	thymic T lymphoma ($CD4^+CD8^+Mac1^+$)
186 F	172 d	massive thymus (1370 mg); enlarged spleen (270 mg).	thymus: 9.3% DN, 71% $CD4^+CD8^+$, 13% $CD4^+$, 1.1% $CD8^{+,}$, 8.2% $CD19^+$, 26% $Mac1^+$ (53% of $CD4^+$ are $Mac1^+$) clonal TCR	thymus: lymphoblasts	nd	thymic T lymphoma ($CD4^+CD8^+Mac1^+$)
361 F	176 d	enlarged spleen (710 mg); enlarged LNs; enlarged liver; enlarged thymus (220 mg).	nd	nd	nd	disseminated myeloid tumour (inferred)
**1111 F**	202 d	enlarged spleen (880 mg) and LNs (240 mg), MLN (220 mg); enlarged thymus (330 mg).	spleen: 31% $CD4^+$, 1.4% $CD8^+$, 59% $CD19^+$, 1.6% $Mac1^+$ $CD19^+$: polyclonal VDJ $CD4^+$: large size, polyclonal TCR	large pleiomorphic cells with prominent nuclear heterochromatin invading lung, spleen, kidneys,	thymus: lymphocytes with occasional large vacuolated blast	disseminated myeloid tumour

			thymus:<br>67% DN, 1.7% $CD4^+CD8^+$,<br>17% $CD4^+$, 4.6% $CD8^+$,<br>56% $CD19^+$, 14% $Mac1^+$	liver, lymph nodes, sternum		
857 F	310 d	massive spleen (1240 mg); enlarged liver, enlarged right uterus;<br>thymus 70 mg.	spleen:<br>15% CD4+, 3.5% CD8+,<br>38% $CD19^+$, 58% $Mac1^+$	nd	nd	disseminated myeloid tumour (inferred)

Abbreviations: nd, not done; fd, found dead; LN, lymph nodes (axillary + brachial + inguinal); MLN, mesenteric LN.

[1]Mouse identification number and sex

[2]Age (in days) when euthanised and autopsied.

[3]Determined by flow cytometry after immunostaining and, in some cases, by PCR analysis of TCRβ and/or VDJ genes. Immunostaining, in parallel, of normal thymi: 5.3% DN, 83% $CD4^+CD8^+$, 6.4% $CD4^+$, 3.8% $CD8^+$ and normal spleens: 17% $CD4^+$, 13% $CD8^+$, 59% $CD19^+$, 5.5% $Mac1^+$.

[4]Determined blinded after staining, by haematologist APN.

[5] Diagnosis of tumour type primarily responsible for morbidity, deduced from autopsy and, where available, immunophenotyping, PCR analysis, and histology of tissues and cytospins. Where no additional data were available, tumour type was inferred by comparison to similar autopsy findings.

26/26 mice in the cohort developed tumours; median survival 140 d (range 69 -310 d). Twelve mice (grey rows) had thymomas (46%); 8/8 immunophenotyped were $CD4^+CD8^+Mac1^+$ T lymphomas. Fourteen mice had enlarged spleens (54%), with myeloid tumour cells infiltrating many tissues.
