# Supplementary material for: MNT suppresses T cell apoptosis via BIM and is critical for T lymphomagenesis
Source: Cell Death Differ. 2023 Feb 8;30(4):1018–32. doi: 10.1038/s41418-023-01119-y (PMC10070419; doi:10.1038/s41418-023-01119-y)
Supplement: Supplementary file 5 — Table S2 [file 41418_2023_1119_MOESM5_ESM.pdf]

Table S2. Tumours arising in *Mnt*$^{+/+}$ *vavP-MYC10*^{hom}/*Rag1Cre* mice.

[1]Mouse	[2]Survival	Autopsy	[3]Immunophenotype	[4]Histology	[4]Cytospin	[5]Diagnosis
919 F	107 d	massive thymus	fd	nd	nd	thymic T lymphoma (inferred)
692 M	110 d	massive thymus (1260 mg); enlarged spleen (230 mg); testicular tumour.	thymus:<br>13% DN, 69% CD4$^+$CD8$^+$, 14% CD4$^+$, 4.4% CD8$^+$, 10% CD19$^+$, 49% Mac1$^+$	nd	nd	thymic T lymphoma (CD4$^+$CD8$^+$Mac1$^+$)
564 M	116 d	massive thymus (1360 mg); enlarged spleen (220 mg).	nd	nd	nd	thymic T lymphoma (inferred)
1128 F	118 d	massive thymus (1040 mg); enlarged spleen (250 mg).	thymus:<br>12% DN, 77% CD4$^+$CD8$^+$, 8.4% CD4$^+$, 2.4% CD8$^{+,}$ 7.2% CD19$^+$, 60% Mac1$^+$<br>*61% CD4$^+$ were Mac1$^+$*<br>spleen:<br>20% CD4$^+$, 3.2% CD8$^+$, 58% CD19$^+$, 12% Mac1$^+$<br>CD4$^+$ cells were CD44$^+$	medium-sized cells with pleiomorphic morphology invading lungs, liver, parotid, lymph nodes, sternum.	**nd**	thymic T lymphoma (CD4$^+$CD8$^+$Mac1$^+$)<br>+<br>disseminated myeloid tumour
454 F	119 d	massive spleen (1000 mg) and LNs (380 mg), MLN (120 mg); enlarged liver;<br>enlarged thymus (120 mg).	nd	nd	nd	disseminated myeloid tumour (inferred)
843 M	128 d	massive spleen (1310 mg) and LNs (130 mg), MLN (130 mg); massive liver<br>enlarged thymus (250 mg).	nd	nd	nd	disseminated myeloid tumour (inferred)
1101 F	129 d	massive thymus (1410 mg); enlarged spleen (220 mg).	thymus:<br>29% DN, 53% CD4$^+$CD8$^+$, 15% CD4$^+$, 3.3% CD8$^+$ 33% CD19$^+$, 2% Mac1$^+$	nd	nd	thymic B lymphoid tumour
1466 M	134 d	massive thymus (1400 mg); spleen 160 mg.	nd	nd	thymus:<br>lymphocytes with vacuolated lymphoblasts	thymic T lymphoma (inferred)

433 M	134 d	massive spleen (1340 mg) and LNs (200 mg), MLN (90 mg);<br>enlarged liver;<br>enlarged thymus (160 mg).	spleen:<br>17% $CD4^+$, 3% $CD8^+$, 30% $CD19^+$, 14% $Mac1^+$	large pleiomorphic cells with prominent nuclear heterochromatin invading lung, spleen, kidneys, liver, LNs.	thymus:<br>vacuolated monocytoid pleiomorphic blasts	disseminated myeloid tumour
1529 M	144 d	massive spleen (1380 mg), enlarged LNs and liver;<br>enlarged thymus (210 mg).	spleen:<br>24% $CD4^+$, 2.5% $CD8^+$ 24% CD19+,17% $Mac1^+$;<br>*Mac1+ cells were transplantable but not CD4+ cells*	nd.	nd	disseminated myeloid tumour
224 M	145 d	massive thymus (1080 mg);<br>massive spleen (1160 mg);<br>pale lungs; enlarged liver	nd	large pleiomorphic cells with prominent nuclear heterochromatin invading lung, spleen, kidneys, liver, LN	nd	thymic T lymphoma<br>+<br>disseminated myeloid tumour
1327 F	146 d	massive spleen (1790 mg) and enlarged liver;<br>thymus 60 mg	spleen:<br>20% $CD4^+$, 2.8% $CD8^+$, 35% $CD19^+$, 22% $Mac1^+$.<br>*Mac1+ cells were transplantable but not CD4+ cells*<br>thymus:<br>DN 40%, 19% $CD4^+CD8^+$, 34% $CD4^+$, 7.2% $CD8^+$, 18% $CD19^+$, 14% $Mac1^+$	nd	nd	disseminated myeloid tumour
943 F	148 d	massive spleen (1500 mg) and liver;<br>enlarged thymus (450 mg)	spleen:<br>39% $CD4^+$, 1.9% $CD8^+$, 18% $CD19^+$, 27% $Mac1^+$	medium-sized blasts invading spleen, kidneys, LN	nd	disseminated myeloid tumour
950 F	148 d	enlarged spleen (360 mg), MLN (170 mg) and pale liver;<br>thymus (100 mg).	spleen:<br>20% $CD4^+$, 3% $CD8^+$, 48% $CD19^+$, 15% $Mac1^+$	nd	nd	disseminated myeloid tumour
1110 F	149 d	massive spleen (990 mg), LNs (390 mg) and MLN (190 mg);<br>enlarged thymus (210 mg).	spleen:<br>14% $CD4^+$, 0.9% $CD8^+$, 8.5% $CD19^+$, 41% $Mac1^+$	large pleiomorphic cells with prominent nuclear heterochromatin invading lung, spleen, liver, lymph nodes, sternum.	Thymus:<br>lymphocytes and small vacuolated blasts	disseminated myeloid tumour

1117 M	149 d	large thymic mass	fd	nd	nd	thymic T lymphoma<br>(inferred)
1006 F	158 d	massive spleen (820 mg) and LNs (150 mg), MLN (80 mg);<br>mottled liver, lungs;<br>enlarged thymus (200 mg).	<u>spleen:</u><br>27% $CD4^+$, 3.2% $CD8^+$,<br>39% $CD19^+$, 13% $Mac1^+$;<br>*Mac1+ cells were transplantable but not CD4+ cells*	large pleiomorphic cells with prominent nuclear heterochromatin invading lung, spleen, kidneys, thymus.	<u>thymus:</u> vacuolated large blasts with pleiomorphic monocytoid morphology and lymphocytes.	disseminated myeloid tumour
377 F	160 d	enlarged spleen, weight loss.	nd	nd	nd	myeloid tumour<br>(inferred)
849 M	166 d	massive spleen (1050 mg), LNs (390 mg), MLN (110 mg);<br>massive liver, pale kidneys;<br>enlarged thymus (300 mg).	<u>spleen:</u><br>13% $CD4^+$, 2% $CD8^+$,<br>33% $CD19^+$, 29% $Mac1^+$	large pleiomorphic cells with prominent nuclear heterochromatin invading lung, spleen, kidneys, liver, lymph nodes.		disseminated myeloid tumour
992 F	167 d	massive spleen (1140 mg), LNs (470 mg), MLN (100 mg);<br>enlarged liver;<br>enlarged thymus (140 mg).	<u>spleen:</u><br>18% $CD4^+$, 2.2% $CD8^+$,<br>44% $CD19^+$, 44% $Mac1^+$<br>*Mac1+ cells were transplantable but not CD4+ cells*	large pleiomorphic cells with prominent nuclear heterochromatin invading lung, spleen, kidneys, liver, parotid, LNs.	<u>thymus:</u><br>lymphocytes and large vacuolated blasts with pleiomorphic monocyte/mΦ morphology.	disseminated myeloid tumour
1108 M	167 d	massive spl (1080 mg);<br>enlarged LNs (130 mg);<br>enlarged pale liver;<br>thymus 80 mg.	nd	Large pleiomorphic cells with prominent nuclear heterochromatin invading liver, LN, sternum.	nd	disseminated myeloid tumour<br>(inferred)
445 F	171 d	spleen (670 mg),<br>LNs (130 mg), MLN (100 mg);<br>enlarged thymus (340 mg).	nd	large pleiomorphic cells with prominent nuclear heterochromatin invading lung, spleen, kidneys, liver, sternum.	<u>thymus:</u><br>lymphocytes, occasional vacuolated blasts.	disseminated myeloid tumour
693 M	177 d	massive mottled spleen (890 mg); enlarged LNs (130 mg), MLN (168 mg);<br>mottled liver;<br>enlarged thymus (170 mg).	<u>spleen:</u><br>14% $CD4^+$, 2% $CD8^+$,<br>17% $CD19^+$, 39% $Mac1^+$	nd	nd	disseminated myeloid tumour
1428 M	178 d	enlarged spleen (400 mg)<br>thymus 40 mg.	nd	nd	nd	myeloid tumour<br>(inferred)

Abbreviations: nd, not done; fd, found dead; LN, lymph nodes (axillary + brachial + inguinal); MLN, mesenteric LN.
[1]Mouse identification number and sex
[2]Age (in d) when euthanised and autopsied.
[3]Determined by flow cytometry after immunostaining and, in some cases, by PCR analysis of TCR and/or VDJ genes. Immunostaining, in parallel, of normal thymi: 5.3% DN, 83% $CD4^+CD8^+$, 6.4% $CD4^+$, 3.8% $CD8^+$ and normal spleens: 17% $CD4^+$, 13% $CD8^+$, 59% $CD19^+$, 5.5% $Mac1^+$.
[4]Determined blinded after staining, by haematologist APN.
[5] Diagnosis of tumour type primarily responsible for morbidity, deduced from autopsy and, where available, immunophenotyping, and histology of tissues and cytospins. 24/24 mice developed tumours (median 147 d, range 107 to 178 d); 2 mice had a thymoma plus disseminated myeloid tumour cells ie 26 tumours in total. 7/26 tumours (27%) were thymomas; 3 were immunophenotyped and 2 were $CD4^+CD8^+Mac1^+$ T lymphomas and 1 was a B lymphoma.18/26 mice (69%) had greatly enlarged spleens; 11/11 immunophenotyped had an increased proportion of myeloid cells, and myeloid tumour cells infiltrated many tissues.
11/26 mice had somewhat enlarged thymi (>100 mg up to 250 mg) and 3 others had thymi of up to 450g as well as splenomegaly.
