# Supplementary material for: MNT suppresses T cell apoptosis via BIM and is critical for T lymphomagenesis
Source: Cell Death Differ. 2023 Feb 8;30(4):1018–32. doi: 10.1038/s41418-023-01119-y (PMC10070419; doi:10.1038/s41418-023-01119-y)
Supplement: Supplementary file 6 — Table S3 [file 41418_2023_1119_MOESM6_ESM.pdf]

Table S3. Tumours arising in *Mnt$^{fl/fl}$ vavP-MYC10^{hom}/Rag1Cre* mice

[1]Mouse	[2]Survival	Autopsy	[3]Immunophenotype	[4]Histology	[4]Cytospin	[5]Diagnosis
344 F	92 d	massive spleen (990 mg);<br>enlarged LNs (130 mg),<br>MLN (290 mg);<br>thymus 90 mg.	Spleen:<br>7.8% CD19$^+$ 32% Mac1$^+$	nd	nd	disseminated myeloid tumour
570 M	109 d	enlarged spleen (320 mg);<br>enlarged LNs near heart and lumbar LN;<br>enlarged pale liver;<br>thymus 60 mg	nd	nd	nd	disseminated myeloid tumour (inferred)
1293 F	117 d	massive spleen (1090 mg);<br>enlarged LNs (130 mg);<br>enlarged liver;<br>thymus 70 mg.	spleen:<br>15% CD4$^+$, 2.7% CD8$^+$,<br>13% CD19$^+$, 23% Mac1$^+$<br>thymus:<br>54% DN, 15% CD4$^+$CD8$^+$,<br>8.2% CD8$^+$, 23% CD4$^+$<br>1.7% CD19$^+$, 53% Mac1$^+$	nd	spleen:<br>large vacuolated blasts among mature myeloid and lymphoid cells.	disseminated myeloid tumour
1142 F	132 d	massive spleen (1080 mg);<br>enlarged LNs (90 mg),<br>MLN (80 mg);<br>pale enlarged liver;<br>thymus 140 mg.	spleen:<br>8.7% CD4$^{+,}$ 5% CD8$^+$,<br>8.4% CD19$^+$, 68% Mac1$^+$,<br>thymus:<br>33% DN, 36% CD4$^+$CD8$^+$,<br>13% CD8$^+$, 18% CD4$^+$,<br>15% CD19$^+$, 12% Mac1$^+$	large pleiomorphic cells with prominent nuclear heterochromatin invading lung, spleen, kidneys, liver, parotid, LNs.	spleen:<br>large vacuolated blasts among mature myeloid and lymphoid cells.	disseminated myeloid tumour
1313 F	136 d	massive spleen (860 mg);<br>enlarged LNs (160 mg);<br>enlarged liver;<br>thymus 40 mg.	spleen:<br>32% CD4$^+$, 4.3% CD8$^+$;<br>32% CD19$^+$, 7.2% Mac1$^+$<br>CD19$^+$ polyclonal VDJ<br>CD4$^+$ polyclonal TCR with one more dominant band.	nd	spleen:<br>large vacuolated blasts among mature myeloid and lymphoid cells<br>thymus:<br>pleiomorphic, large vacuolated blasts among lymphocytes.	disseminated myeloid tumour

743 F	144 d	massive spleen (840 mg);<br>enlarged LNs (140 mg), MLN (90 mg);<br>massive liver;<br>abnormal lungs;<br>enlarged thymus (160 mg).	spleen:<br>28% $CD4^+$, 6.1% $CD8^+$, 26% $CD19^+$, 19% $Mac1^+$	large pleiomorphic cells invading lung, kidneys, liver, LNs, sternum.		disseminated myeloid tumour
838 M	144 d	enlarged spleen (700mg);<br>enlarged LNs (440mg), MLN (80mg);<br>hard subcutaneous lump;<br>massive pale spotted liver;<br>enlarged thymus (120 mg).	spleen:<br>17% $CD4^+$, 5.4% $CD8^+$, 18% $CD19^+$, 36% $Mac1^+$	nd	spleen:<br>large vacuolated blasts demonstrating erythrophagocytosis among mature myeloid and lymphoid cells.	disseminated myeloid tumour
1316 M	146 d	enlarged spleen (560 mg)<br>thymus 20 mg.	spleen:<br>18% $CD4^+$, 1.8% $CD8^+$<br>34% $CD19^+$, 16% $Mac1^+$		thymus:<br>lymphocytes and occasional large vacuolated blasts.	disseminated myeloid tumour
450 M	147 d	massive spleen (890 mg);<br>enlarged thymus (220 mg)	nd	large pleiomorphic cells with prominent nuclear heterochromatin invading lung, spleen, LNs, sternum.	spleen:<br>large vacuolated blasts among mature myeloid cells and lymphoid cells.	disseminated myeloid tumour (inferred)
420 F	151 d	enlarged spleen (560 mg) and LNs (160 mg)<br>enlarged thymus (110 mg).	nd	large pleiomorphic cells with prominent nuclear heterochromatin morphology invading lung, spleen, kidneys, liver, LNs, sternum	spleen:<br>large vacuolated blasts among mature myeloid cells and lymphoid cells.	disseminated myeloid tumour (inferred)
798 M	154 d	enlarged spleen (300 mg);<br>hard tumour on front limb;<br>thymus 70 mg.	spleen:<br>23% $CD4^+$, 6.2% $CD8^+$, 47% $CD19^+$, 16% $Mac1^+$	large blasts diffusely invading spleen.	nd	disseminated myeloid tumour (inferred)
701 F	155 d	massive spleen	fd	nd	nd	myeloid tumour (inferred)

834 M	158 d	massive spleen (880 mg);<br>enlarged LNs (210 mg),<br>MLN (170 mg);<br>massive pale liver;<br>lump on right front paw.<br>enlarged thymus (110 mg)	<u>spleen:</u><br>8.5% $CD4^+$, 5% $CD8^+$,<br>7.8% $CD19^+$, 32% $Mac1^+$	nd	nd	disseminated myeloid tumour
1276 M	165 d	massive spleen (810 mg);<br>normal size LNs;<br>thymus 30 mg.	<u>spleen:</u><br>20% $CD4^+$, 6.1% $CD8^+$,<br>34% $CD19^+$, 24% $Mac1^+$,	nd	nd	splenic myeloid tumour
720 M	166 d	massive spleen (1030 mg);<br>tumour on neck;<br>thymus 80mg	<u>spleen:</u><br>24% $CD4^+$, 7.2% $CD8^+$,<br>19% $CD19^+$, 35% $Mac1^+$	nd	nd	disseminated myeloid tumour
465 F	171 d	enlarged spleen (710 mg)<br>and LNs (230 mg);<br>thymus 50 mg.	nd	large pleiomorphic cells with prominent nuclear heterochromatin invading lung, spleen, kidneys, liver, sternum.	nd	disseminated myeloid tumour
1394 F	176 d	enlarged spleen (400 mg);<br>enlarged, pale liver with white spots;<br>ascites;<br>thymus 20 mg	<u>spleen:</u><br>17% $CD4^+$, 2.4% $CD8^+$<br>51% $CD19^+$, 5% $Mac1^+$<br>CD19+ VDJ polyclonal<br>CD4+ TCR polyclonal with 1 more dominant band	nd	<u>spleen:</u><br>large vacuolated blasts demonstrating erythrophagocytosis among mature myeloid and lymphoid cells	disseminated myeloid tumour
831 F	177 d	massive spleen (800 mg);<br>massive LNs (190 mg),<br>MLN (280 mg);<br>enlarged mottled liver;<br>pale kidneys;<br>ascites;<br>enlarged thymus (130 mg).	nd	large pleiomorphic cells with prominent nuclear heterochromatin invading spleen, lung, kidneys, liver, LNs, sternum.	nd	disseminated myeloid tumour

262 F	178 d	enlarged spleen (540 mg) and LNs (130 mg); enlarged liver; pale lungs; enlarged thymus (110 mg).	nd	large pleiomorphic cells with prominent nuclear heterochromatin invading lung, kidneys, liver, LN, sternum.	spleen: occasional large vacuolated blasts among mature myeloid and lymphoid cells	disseminated myeloid tumour
698 M	183 d	massive spleen (890 mg); enlarged LNs (100 mg), MLN (90mg); enlarged pale liver; thymus 90 mg.	spleen: 18 % $CD4^+$, 5.2% $CD8^+$ 17% $CD19^+$, 21% $Mac1^+$	nd	nd	disseminated myeloid tumour
1358 F	183 d	massive spleen (900 mg) and LNs (330 mg), MLN (190 mg); enlarged liver enlarged thymus 130 mg.	spleen: 18% $CD4^+$, 2% $CD8^+$, 7.6% $CD19^+$, 18% $Mac1^+$	nd	thymus: occasional pleiomorphic medium to large vacuolated blasts among lymphocytes	disseminated myeloid tumour
443 M	189 d	enlarged spleen (230 mg) and LNs (140 mg); thymus 30 mg.	nd	large cells with prominent nuclear heterochromatin invading lung, spleen, kidneys, liver, LN, sternum.	nd	disseminated myeloid tumour
529 F	205 d	massive spleen (980 mg) and LNs (270 mg), MLN (140 mg); thymus 70 mg.	nd	large cells with prominent nuclear heterochromatin invading lung, spleen, liver, LNs.	nd	disseminated myeloid tumour
543 F	213 d	massive spleen (940 mg); enlarged LNs (110 mg); enlarged pale liver; white spots on lung enlarged thymus (230 mg).	nd	large cells with prominent nuclear heterochromatin invading lung, spleen, kidneys, liver, LN, sternum.	nd	disseminated myeloid tumour
457 F	270 d	massive spleen (1280 mg); enlarged MLN (210 mg);	nd	nd	nd	disseminated myeloid tumour (inferred)

		pale kidneys and lungs;<br>enlarged pale liver;<br>thymus 90 mg				
474 F	330 d	massive mottled lumpy spleen (840 mg);<br>pale lumpy lung;<br>pale rough liver;<br>fluid-filled cyst at bottom of right kidney;<br>enlarged thymus (250 mg)	nd	nd	nd	disseminated myeloid tumour (inferred)

Abbreviations: nd, not done; fd, found dead; LN, lymph nodes (axillary + brachial + inguinal); MLN, mesenteric LN.
[1]Mouse identification number and sex
[2]Age (in days) when euthanised and autopsied.
[3]Determined by flow cytometry after immunostaining and, in some cases, by PCR analysis of TCR and/or VDJ genes.
Immunostaining, in parallel, of normal thymi: 5.3% DN, 83% $CD4^+CD8^+$, 6.4% $CD4^+$, 3.8% $CD8^+$ and normal spleens: 17% $CD4^+$, 13% $CD8^+$, 59% $CD19^+$, 5.5% $Mac1^+$.
[4]Determined blinded after staining, by haematologist APN.
[5] Diagnosis of tumour type primarily responsible for morbidity, deduced from autopsy and, where available, immunophenotyping, PCR analysis, and histology of tissues and cytospins. If no additional data available, tumour type was inferred by comparison to similar autopsy findings.
26/26 mice in cohort developed tumours (median 162 d, range 92 to 330 d). 0/26 developed thymomas; 26/26 had tumours that enlarged the spleen and infiltrated many other tissues.13/26 splenic tumours were immunophenotyped; 13/14 had an increased proportion of $Mac1^+$ cells and a decreased proportion of $CD19^+$ B cells; in mouse #1394, the enlarged spleen had normal composition but the liver was enlarged and pale, with white spots, consistent with infiltration by myeloid tumour cells. 11/26 mice had somewhat enlarged thymi (>100 mg up to 250 mg); 2/2 immunophenotyped (#1293, #1142) were found to be infiltrated by $Mac1^+$ cells.
