# Supplementary material for: MNT suppresses T cell apoptosis via BIM and is critical for T lymphomagenesis
Source: Cell Death Differ. 2023 Feb 8;30(4):1018–32. doi: 10.1038/s41418-023-01119-y (PMC10070419; doi:10.1038/s41418-023-01119-y)
Supplement: Supplementary file 7 — Table S4 [file 41418_2023_1119_MOESM7_ESM.pdf]

**Table S4. Transplantation of cells from enlarged spleens of *Mnt*$^{+/+}$ *MYC10*$^{hom}$*/Rag1Cre* mice**[1]

| Donor splenic tumour (Ly5.2$^+$) Weight | Injected spleen cells | Survival | Pathology | Immunophenotype of splenic tumour in recipient mice |
|---|---|---|---|---|
| #992 F<br>1140 mg | Mac1$^+$ | 22, 24d | enlarged spleen; enlarged pale liver. | Ly5.2$^+$Mac1$^+$ |
| | CD4$^+$ | 32d | splenomegaly | Ly5.2$^+$Mac1$^+$ |
| #1006 F<br>820 mg | Mac1$^+$ | 22d | enlarged spleen, liver, ascites | Ly5.2$^+$Mac1$^+$ |
| | CD4$^+$ | 49d | enlarged spleen, liver. | Ly5.2$^+$Mac1$^+$ |
| #1529 M<br>1380 mg | Mac1$^+$ | 44d, 44d | enlarged spleen, liver. | Ly5.2$^+$Mac1$^+$ |
| | CD4$^+$ | 84d,<br>> 182d | enlarged spleen | Ly5.2$^+$Mac1$^+$ |
| #1327 F<br>1790 mg | Mac1$^+$ | 22d, 22d | enlarged spleen, liver. | Ly5.2$^+$Mac1$^+$ |
| | CD4$^+$ | 22d, 32d | enlarged spleen, liver. | Ly5.2$^+$Mac1$^+$ |

[1]Mac1$^+$ and CD4$^+$ cells were isolated by flow cytometry from enlarged spleens of indicated sick Ly5.2$^+$*Mnt*$^{+/+}$ *MYC10*$^{hom}$*/Rag1Cre* mice and injected into non-irradiated Ly5.1$^+$ C57BL/6 recipient mice (5x10$^5$ cells/recipient) of the same sex, which were monitored for tumour development. Spleen cells from sick recipient mice were stained with fluor-labelled antibodies to CD19, Mac1, CD4, CD8, Ly5.1 and Ly5.2 to determine the immunophenotype of the transplantable Ly5.2$^+$ cells. Note that although some mice transplanted with CD4$^+$ cells developed tumours, these were myeloid rather than T lymphoid and presumably arose from Mac1$^+$ cells contaminating the sorted CD4$^+$ cells.
