# Supplementary material for: MNT suppresses T cell apoptosis via BIM and is critical for T lymphomagenesis
Source: Cell Death Differ. 2023 Feb 8;30(4):1018–32. doi: 10.1038/s41418-023-01119-y (PMC10070419; doi:10.1038/s41418-023-01119-y)
Supplement: Supplementary file 8 — Table S5 [file 41418_2023_1119_MOESM8_ESM.pdf]

Table S5 Tumours arising in irradiated C57BL/6 mice

Mouse	Survival	Autopsy	Immunophenotype	Inferred tumour type
WT				
#2318 F	153d	massive thymus (970 mg)<br>ascites	thymus:<br>0.5% DN, 98% DP, 0.2% $CD4^+$, 1% $CD8^+$	$CD4^+CD8^+$ thymic T lymphoma
#2325 F	130d	massive thymus (365mg)<br>enlarged spleen (612 mg)	thymus:<br>5.5% DN, 13% DP, 0.1% $CD4^+$, 81% $CD8^+$<br>spleen:<br>0.4% $CD4^+$, 80% $CD8^+$, 0.5% $CD19^+$, 2.8% $Mac1^+$	$CD8^+$ T lymphoma in thymus and spleen
#2326 F	141d	enlarged thymus (240 mg)<br>enlarged spleen (288 mg)	thymus:<br>12% DN, 17% DP, 0.2% $CD4^+$, 62% $CD8^+$<br>spleen:<br>3.3% $CD4^+$, 59% $CD8^+$, 17% $CD19^+$, 4.7% $Mac1^+$	$CD8^+$ T lymphoma in thymus and spleen
#2254 M	178d	massive thymus (660 mg)<br>enlarged spleen (480 mg)	thymus:<br>2.9% DN, 85% DP, 0.9% $CD4^+$, 11% $CD8^+$<br>spleen:<br>3% $CD4^+$, 7.3% $CD8^+$, 40% DP, 35% $CD19^+$, 12% $Mac1^+$	$CD4^+CD8^+$ T lymphoma in thymus and spleen
#2256 M	160d	massive thymus (330 mg)<br>enlarged spleen (430 mg)<br>enlarged liver, ascites	thymus:<br>16% DN, 15% DP, 4.4% $CD4^+$, 65% $CD8^+$<br>spleen:<br>16% $CD4^+$, 13% $CD8^+$, 37% $CD19^+$, 23% Mac1+	$CD8^+$ thymic T lymphoma
#2268 F	142d	massive thymus (950 mg)	thymus:<br>2% DN, 97% DP, 0.8% $CD4^+$, 0.4% $CD8^+$<br>spleen:<br>18% $CD4^+$, 12% $CD8^+$, 49% $CD19^+$, 11% $Mac1^+$	$CD4^+CD8^+$ thymic T lymphoma
#2344 F	183d	~normal thymus (90 mg)<br>enlarged spleen (476 mg)	thymus:<br>2.9% DN, 86% DP, 11% $CD4^+$, 0.7% $CD8^+$	$CD4^+CD8^+$ splenic T lymphoma

			<u>spleen:</u><br>0.4% $CD4^+$, 3.3% $CD8^+$, 74% DP, 7.6% $CD19^+$, 2.5% $Mac1^+$	
#2348 F	203d	~ normal thymus (103 mg)<br>enlarged spleen (346 mg)<br>enlarged liver	<u>thymus:</u><br>15 % DN, 51 % DP, 5.5 % $CD4^+$, 29 % $CD8^+$, 13% $CD19^+$, 0.7% $Mac1^+$<br><u>spleen:</u><br>0.9 % $CD4^+$, 18 % $CD8^+$, 30 % DP, 8.5 % $CD19^+$, 2.3 % $Mac1^+$	mixed $CD4^+CD8^+$ and $CD8^+$ T lymphoma in spleen
#2358 F	166d	hunched, lethargic, laboured breathing, enlarged spleen	nd	lymphoma in thymus and spleen
#2408 F	145d	enlarged thymus (224 mg) and spleen (634 mg)<br>enlarged LNs<br>pale enlarged liver	<u>thymus:</u><br>11% DN, 35% DP, 4.6% $CD4^+$, 50% $CD8^+$<br><u>spleen:</u><br>0.04 % $CD4^+$, 71 % $CD8^+$, 0.2% DP, 5.9 % $CD19^+$, 1.1 % $Mac1^+$	$CD8^+$ T lymphoma in thymus and spleen
Mnt $^{+/+}$ ***Rag1*** $^{Cre/+}$				
# 2257 F	218d	massive thymus (960 mg)	<u>thymus:</u><br>28% DN, 55% DP, 13% $CD4^+$, 4.2 % $CD8^+$, 18% $Mac1^+$, 2.1% $CD19^+$	mixed lymphoid/myeloid tumour in thymus
#2269M	159d	massive thymus (640 mg)<br>enlarged spleen (420 mg)	nd	lymphoma in thymus and spleen
#2270 M	179d	massive thymus (740mg)	nd	lymphoma in thymus
#2319 F	208d	massive thymus (820 mg)	<u>thymus:</u><br>3.5 % DN, 65% DP, 2.8 % $CD4^+$, 28% $CD8^+$	mixed $CD4^+CD8^+$ and $CD8^+$ T lymphoma in thymus
#2345 M	190d	massive thymus (376 mg)<br>enlarged spleen (200 mg)<br>pale enlarged liver	<u>thymus:</u><br>5.0 % DN, 55% DP, 3.2 % $CD4^+$, 37 % $CD8^+$<br><u>spleen:</u>	mixed $CD4^+CD8^+$ and $CD8^+$ T lymphoma in thymus and spleen

			3.9 % $CD4^+$, 21 % $CD8^+$, 45% DP, 19% $CD19^+$, 2.9 % $Mac1^+$	
#2346 M	284d	enlarged thymus (144 mg)<br>enlarged spleen (505 mg)<br>pale liver<br>tumours in both kidneys	nd	disseminated lymphoma
# 2349 F	199d	enlarged thymus (241 mg)<br>enlarged spleen (319 mg)<br>pale enlarged liver,<br>enlarged LNs	<u>thymus:</u><br>2.2% DN, 91% DP, 4.7% $CD4^+$, 2.3 % $CD8^+$<br><u>spleen:</u><br>3.2 % $CD4^+$, 31 % $CD8^+$, 5.1% DP, 9.4 % $CD19^+$, 0.8 % $Mac1^+$	$CD4^+CD8^+$ T lymphoma in thymus and $CD8^+$ T lymphoma in spleen
# 2360 F	221d	massive thymus (743 mg)	2.4 % DN, 95% DP, 1.3 % $CD4^+$, 1.8 % $CD8^+$	$CD4^+CD8^+$ T lymphoma in thymus
# 2409 F	167d	massive thymus (1226 mg)<br>pale liver	4.9 % DN, 80 % DP, 3.1 % $CD4^+$, 12 % $CD8^+$	$CD4^+CD8^+$ T lymphoma in thymus

All mice were on a C57BL/6 background. Mouse identification number and sex is indicated.
10/14 mice in irradiated *WT* cohort developed terminal tumours. Three lived > 350d (#2267, #2343, #2347) and 1(#2420) is still healthy at 211d.
9/10 mice in irradiated *Mnt*$^{+/+}$ *Rag1*$^{Cre/+}$ cohort developed terminal tumours and 1/10 lived >350d (#2359).
0/15 *Mnt*$^{fl/fl}$ *Rag1Cre* mice have developed tumours thus far. # 2252 died at 97d but had no tumour; 9 lived >350d without tumours and 5 are still healthy, aged 206 to 255d when Figure 8A was prepared.
