# Supplementary material for: MNT suppresses T cell apoptosis via BIM and is critical for T lymphomagenesis
Source: Cell Death Differ. 2023 Feb 8;30(4):1018–32. doi: 10.1038/s41418-023-01119-y (PMC10070419; doi:10.1038/s41418-023-01119-y)

Fig. 1A Nguyen *et al*

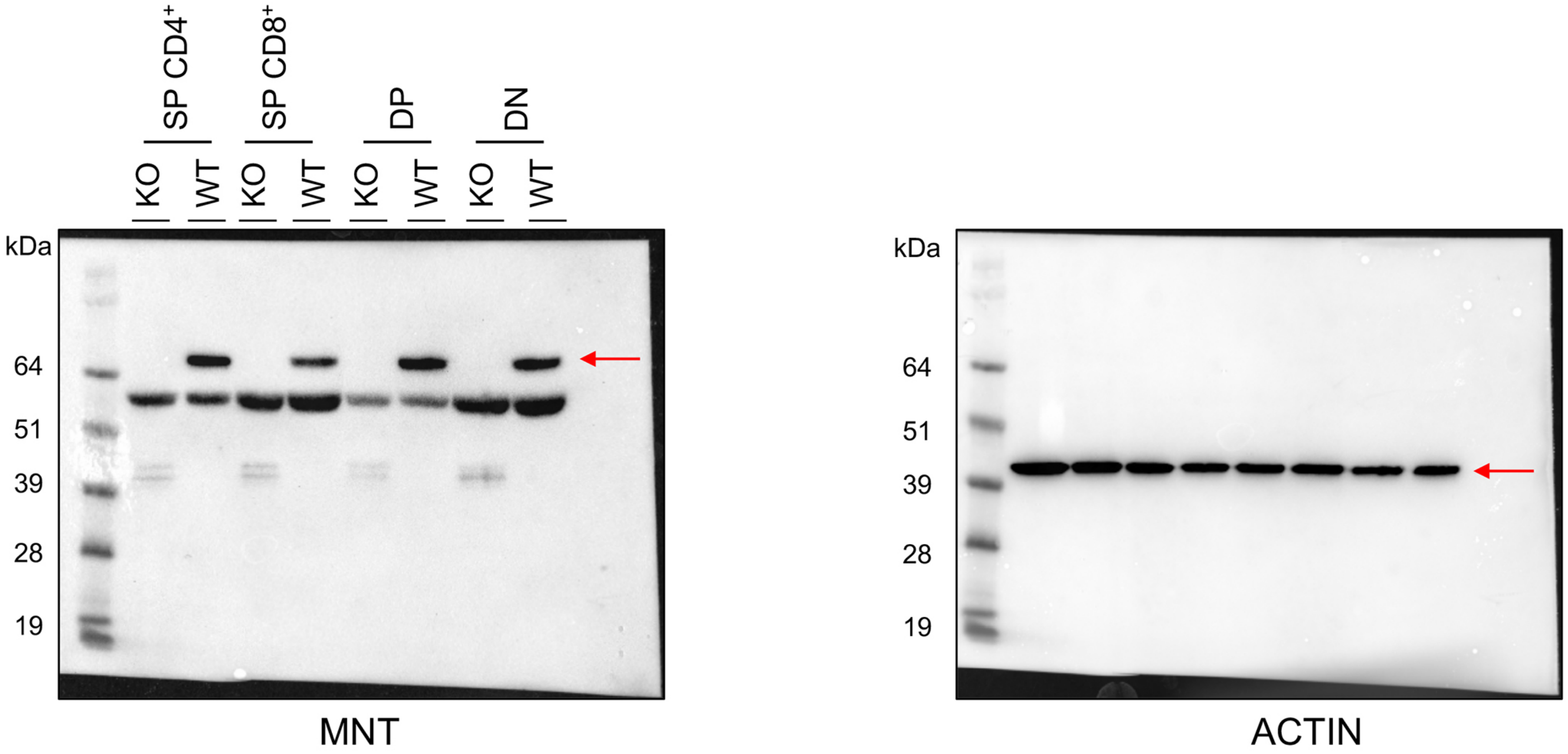

→ specific band

Fig. 3D Nguyen *et al*

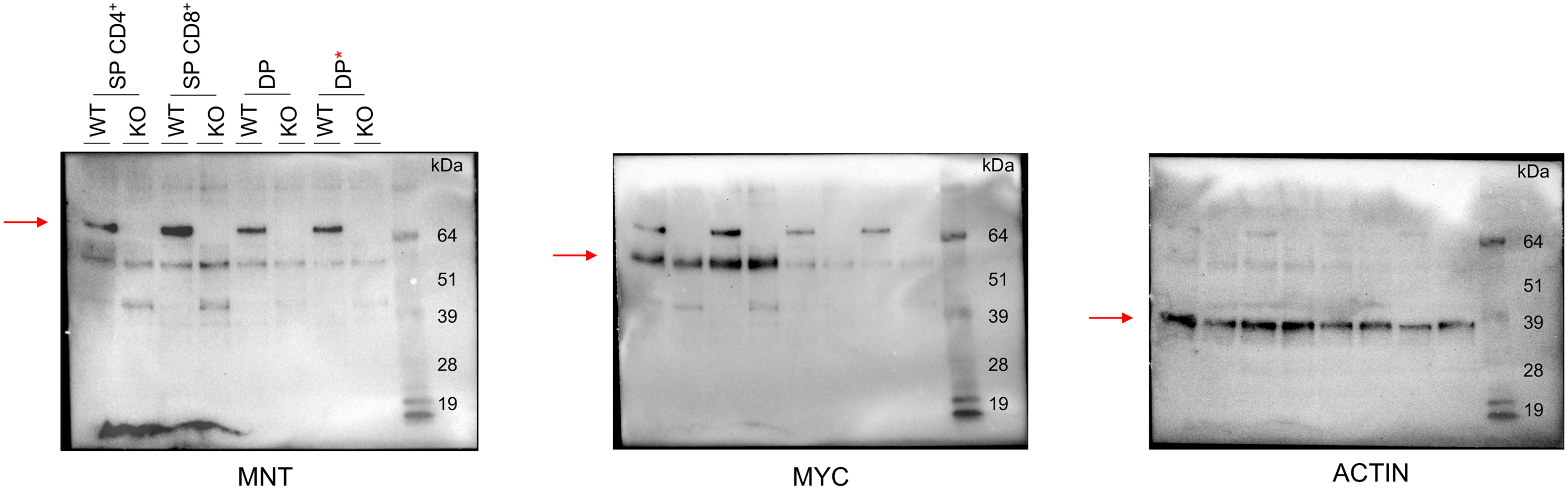

→ specific band

* sorted DP T cells from different mice; not included

Fig. 4E Nguyen *et al*

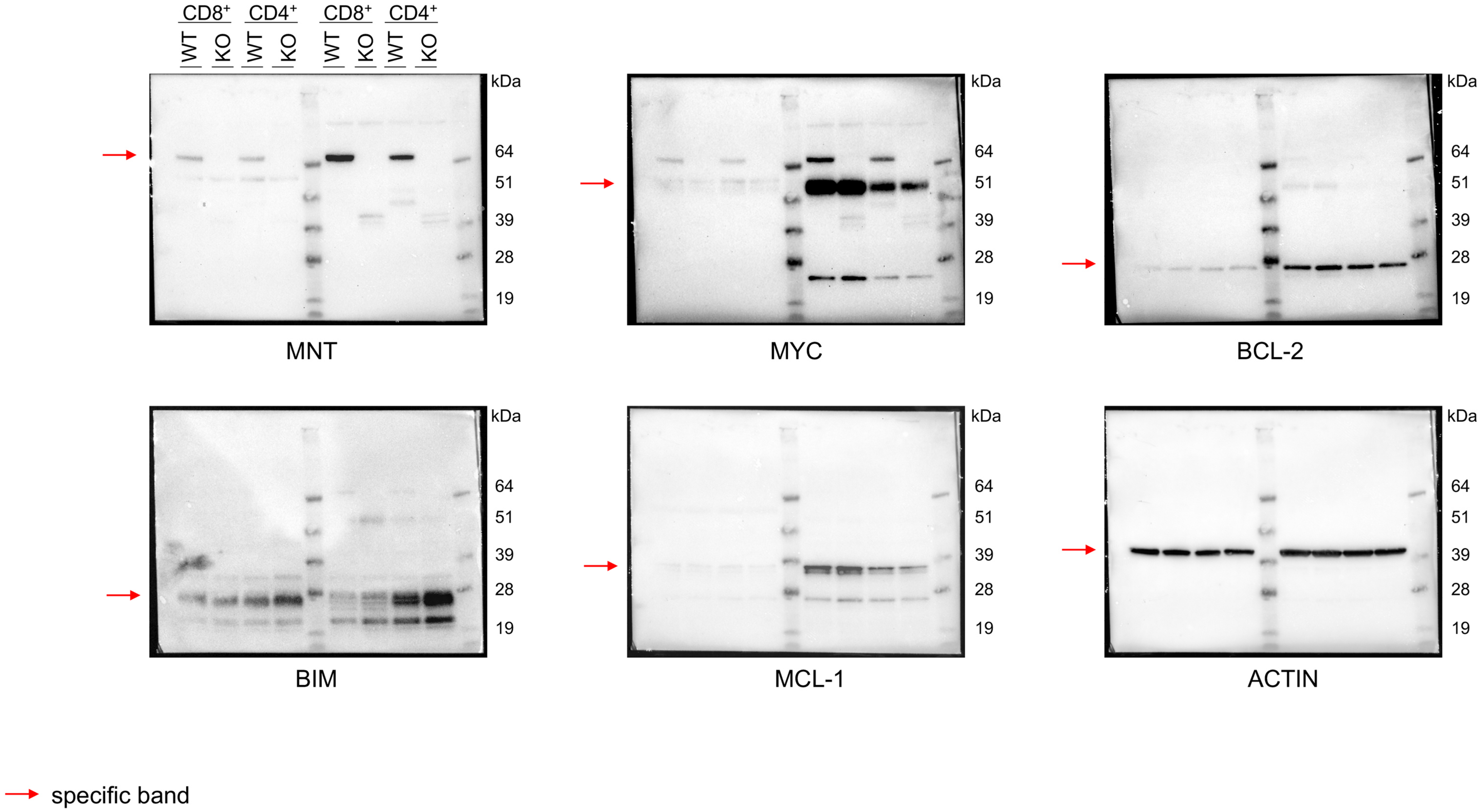

Fig. 5A Nguyen *et al*

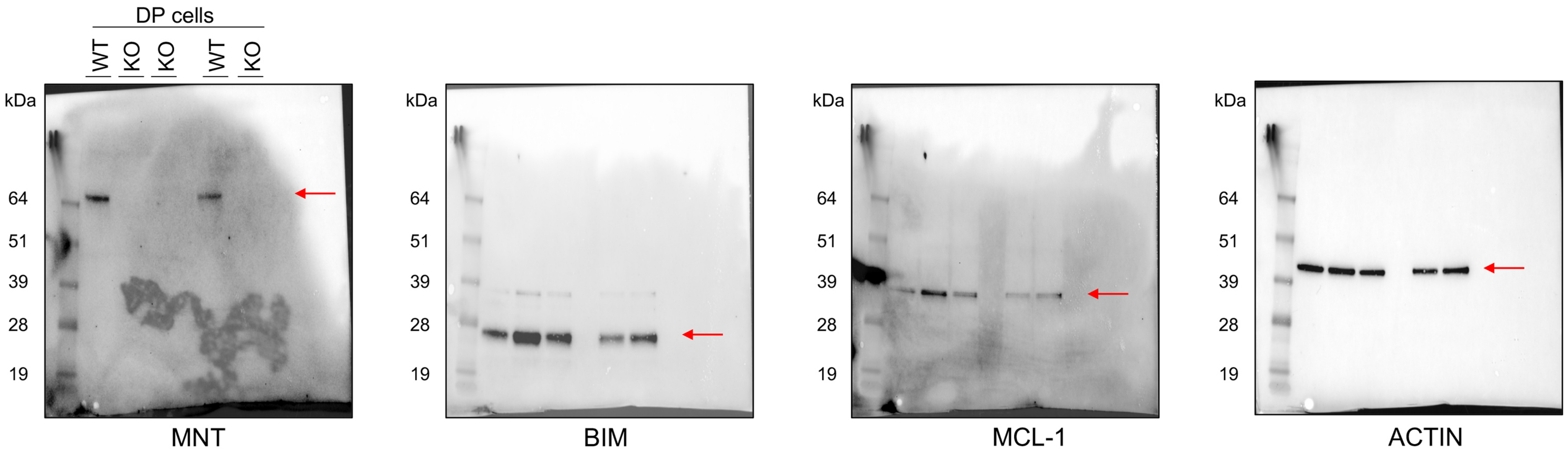

→ specific band

Fig. 7D Nguyen *et al*

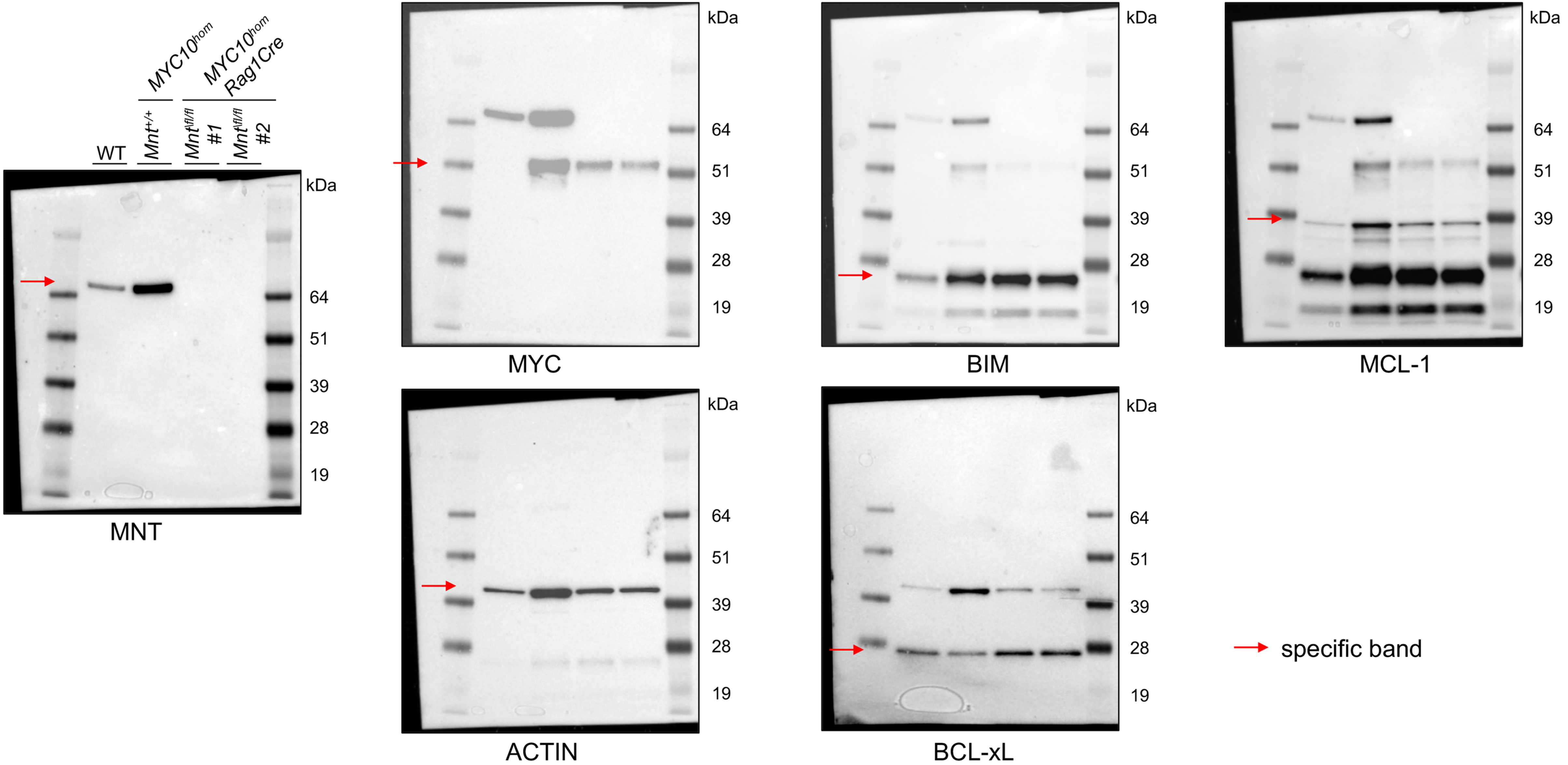

Figure 8 D pre-assembly

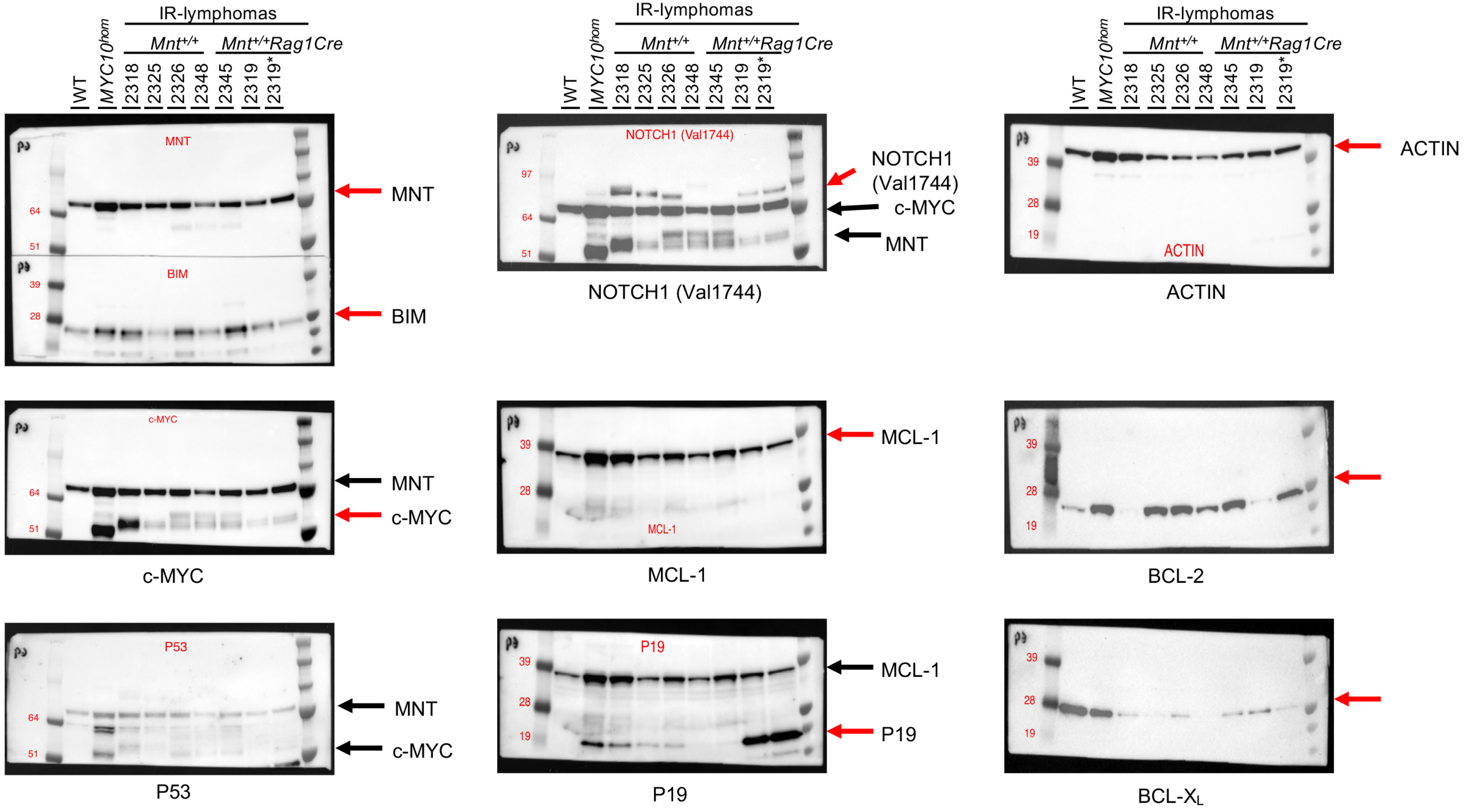

Fig. S5C Nguyen *et al*

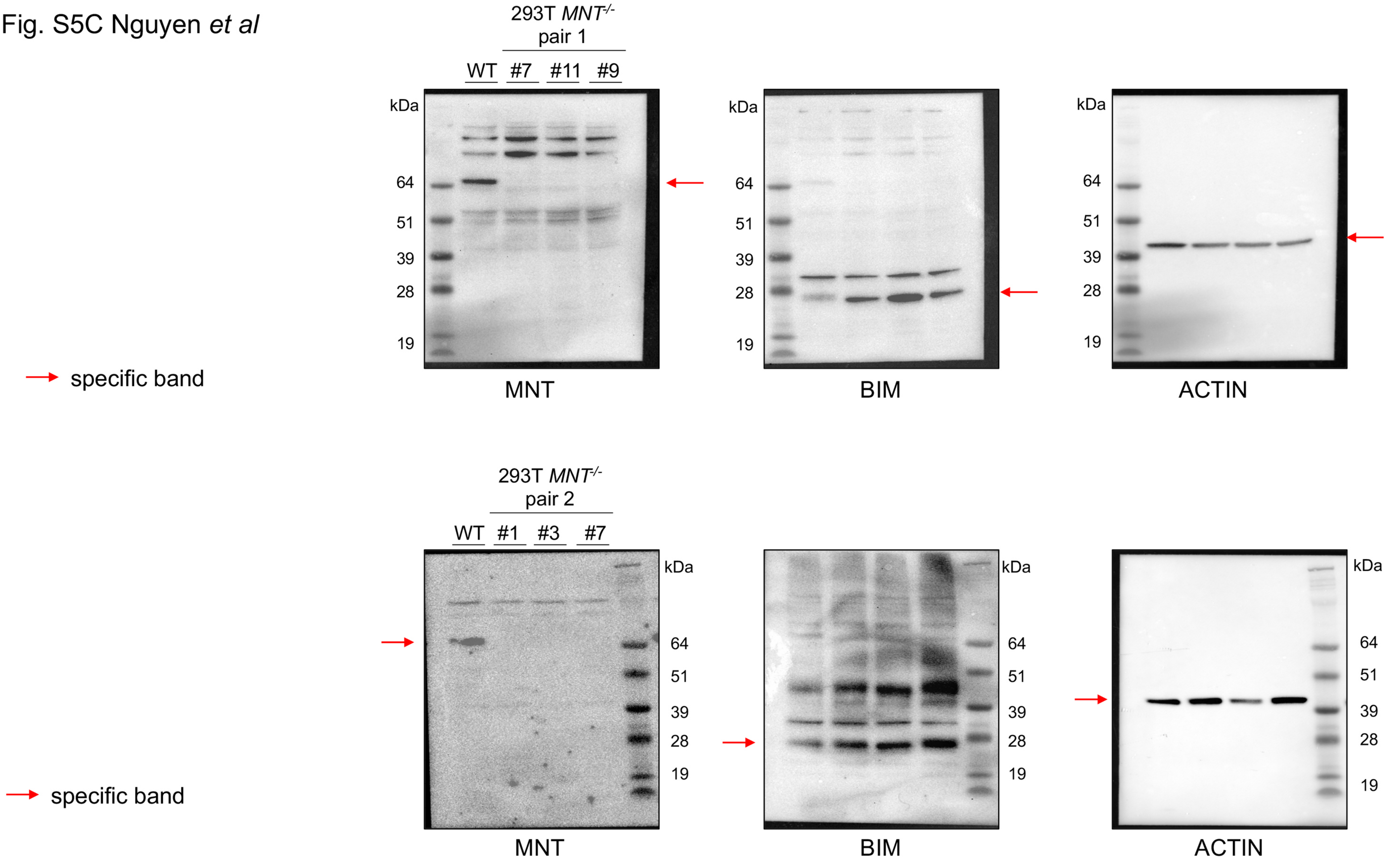

Fig. S5E Nguyen *et al*

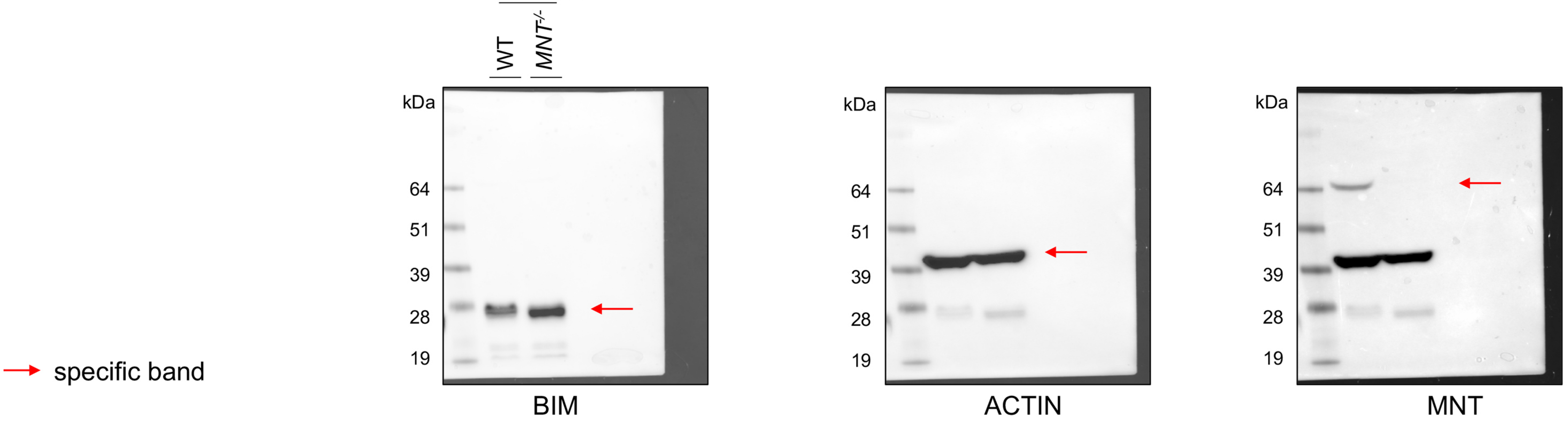

Fig. S5F Nguyen *et al*

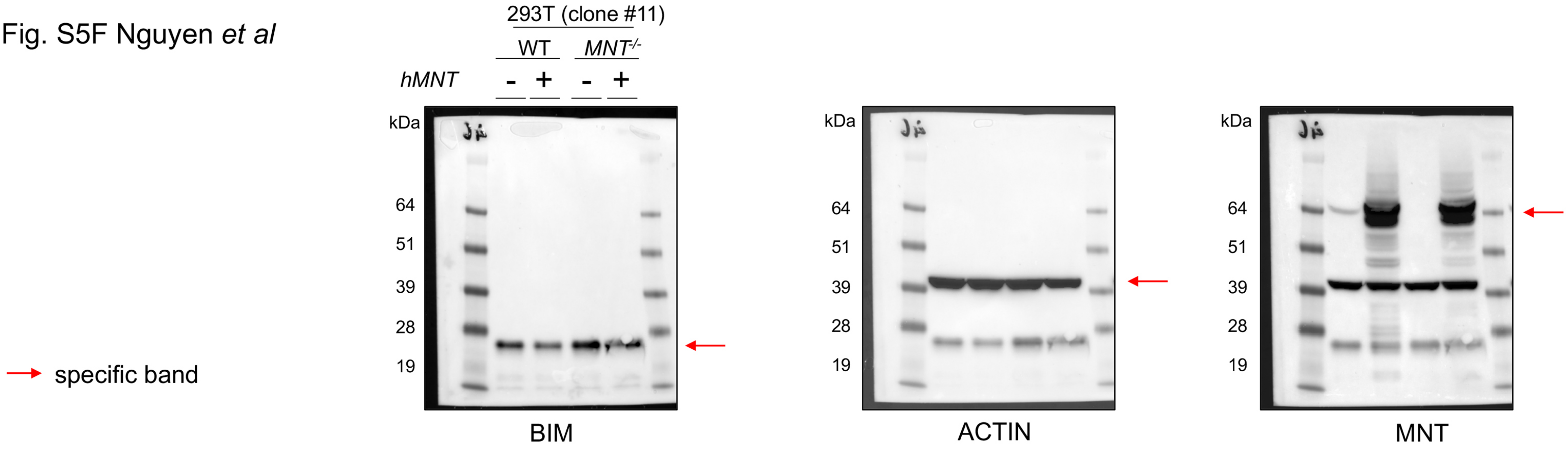

Fig. S5G Nguyen *et al*

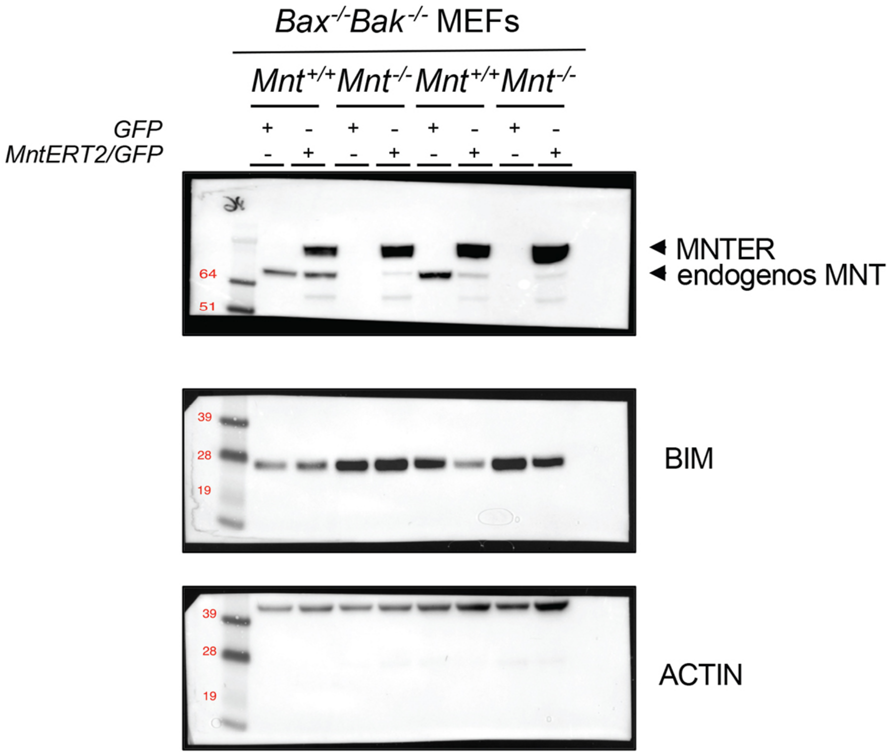

Fig. S6C Nguyen *et al*

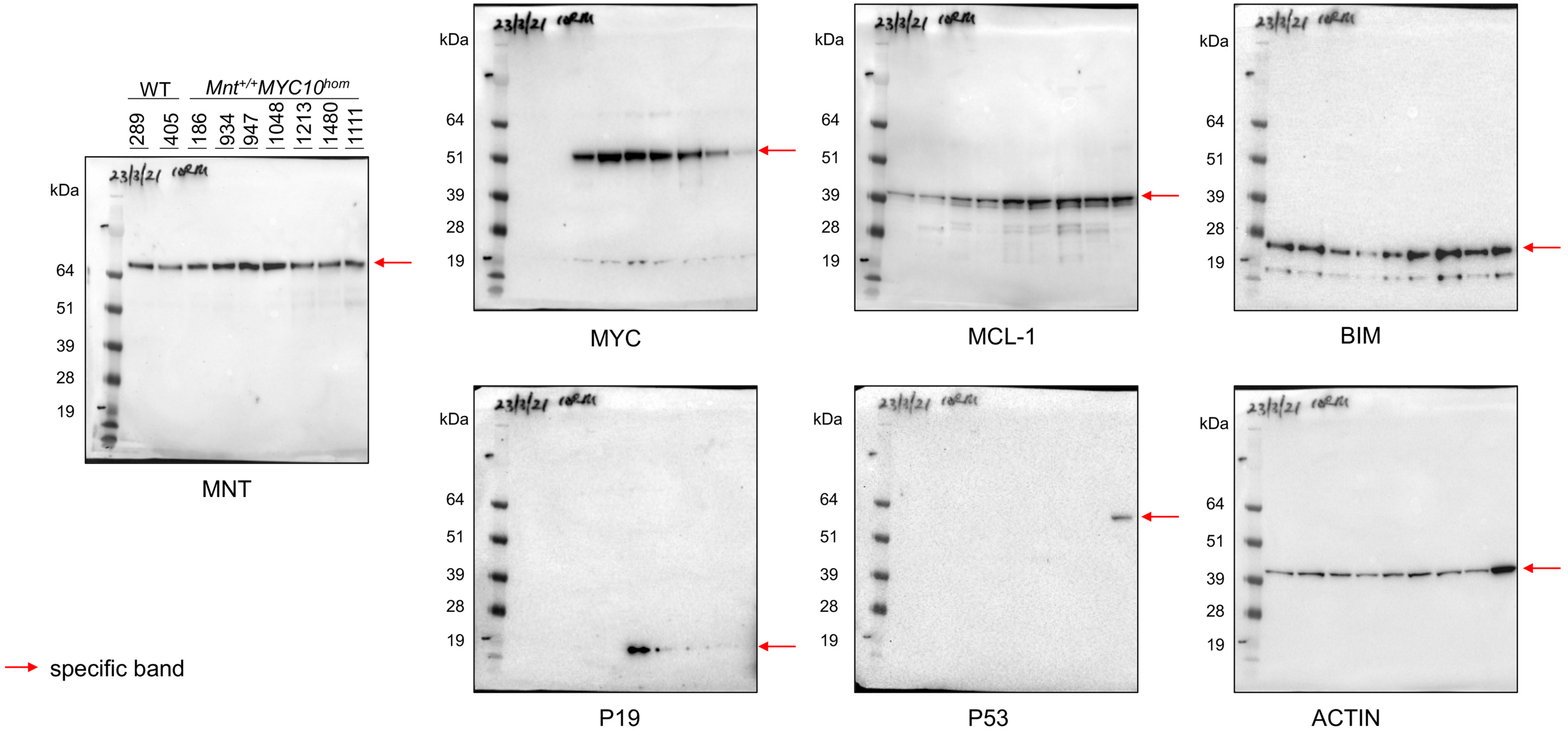

Supplement: Supplementary file 9 — Pre-AssemblyWestern blots [file 41418_2023_1119_MOESM9_ESM.pdf]
